# Isolation and In Silico Anti-SARS-CoV-2 Papain-Like Protease Potentialities of Two Rare 2-Phenoxychromone Derivatives from *Artemisia* spp.

**DOI:** 10.3390/molecules27041216

**Published:** 2022-02-11

**Authors:** Yerlan M. Suleimen, Rani A. Jose, Raigul N. Suleimen, Christoph Arenz, Margarita Ishmuratova, Suzanne Toppet, Wim Dehaen, Aisha A. Alsfouk, Eslam B. Elkaeed, Ibrahim H. Eissa, Ahmed M. Metwaly

**Affiliations:** 1 The International Centre for Interdisciplinary Solutions on Antibiotics and Secondary Metabolites, Republican Collection of Microorganisms, Nur-Sultan 010000, Kazakhstan; syerlan75@yandex.kz; 2 The Laboratory of Engineering Profile of NMR Spectroscopy, Sh. Ualikhanov Kokshetau University, Kokshetau 020000, Kazakhstan; 3 Molecular Design & Synthesis, Department of Chemistry, Catholic University of Leuven, B-3001 Heverlee, Belgium; alphmanie@gmail.com (R.A.J.); tsuzanne@kuleuven.be (S.T.); wim.dehaen@kuleuven.be (W.D.); 4 Department of Natural Science, Faculty of Technical Physics, L.N. Gumilyov Eurasian National University, Nur-Sultan 010010, Kazakhstan; 5 Institut für Chemie der Humboldt, Universität zu Berlin, D-12489 Berlin, Germany; arenzchr@hu-berlin.de; 6 Department of Botany, E.A. Buketov Karaganda University, Karaganda 100024, Kazakhstan; margarita.ishmur@mail.ru; 7 Department of Pharmaceutical Sciences, College of Pharmacy, Princess Nourah Bint Abdulrahman University, P.O. Box 84428, Riyadh 11671, Saudi Arabia; aaalsfouk@pnu.edu.sa; 8 Department of Pharmaceutical Sciences, College of Pharmacy, AlMaarefa University, Ad Diriyah, Riyadh 13713, Saudi Arabia; ikaeed@mcst.edu.sa; 9 Pharmaceutical Medicinal Chemistry & Drug Design Department, Faculty of Pharmacy (Boys), Al-Azhar University, Cairo 11884, Egypt; ibrahimeissa@azhar.edu.eg; 10 Pharmacognosy and Medicinal Plants Department, Faculty of Pharmacy (Boys), Al-Azhar University, Cairo 11884, Egypt; 11 Biopharmaceutical Products Research Department, Genetic Engineering and Biotechnology Research Institute, City of Scientific Research and Technological Applications (SRTA-City), Alexandria 21934, Egypt

**Keywords:** *Artemisia commutate*, 2-phenoxychromones, SARS-CoV-2, COVID-19 Papain-Like Protease, molecular docking, molecular fingerprints, ADMET, MD simulations

## Abstract

Two rare 2-phenoxychromone derivatives, 6-demethoxy-4`-O-capillarsine (**1**) and tenuflorin C (**2**), were isolated from the areal parts of *Artemisia commutata* and *A. glauca,* respectively, for the first time. Being rare in nature, the inhibition potentialities of **1** and **2** against SARS-CoV-2 was investigated using multistage in silico techniques. At first, molecular similarity and fingerprint studies were conducted for **1** and **2** against co-crystallized ligands of eight different COVID-19 enzymes. The carried-out studies indicated the similarity of **1** and **2** with **TTT**, the co-crystallized ligand of COVID-19 Papain-Like Protease (PLP), (PDB ID: 3E9S). Therefore, molecular docking studies of **1** and **2** against the PLP were carried out and revealed correct binding inside the active site exhibiting binding energies of −18.86 and −18.37 Kcal/mol, respectively. Further, in silico ADMET in addition to toxicity evaluation of **1** and **2** against seven models indicated the general safety and the likeness of **1** and **2** to be drugs. Lastly, to authenticate the binding and to investigate the thermodynamic characters, molecular dynamics (MD) simulation studies were conducted on **1** and PLP.

## 1. Introduction

Natural products have been utilized in different folklores to treat many illnesses since ancient times [1,2,3]. The research in the field of natural product chemistry identified various bioactive metabolites that are engaged with the bioactivities such as diterpenes [4], phenolics [5], sesquiterpenes [6,7], steroids [8], flavonoids [9,10], alkaloids [11], saponins [12,13,14], isochromenes [15], benzofuran glucosides [16], and α-pyrones [17,18]. 

The chemical composition as well as the biological activities of plants that belong to the genus *Artemisia* L. (family Asteraceae) drew the attention of scientists to explore and investigate. For example, anhydroaustricin, epiashantin, and matricarin are biologically active compounds that were isolated from *A. albida* [19], *A. sieversiana* [20], and *A. austriaca* [21], respectively. Besides, cirsineol is an active lactone that was reported in *A. umbrosa* [22]. Also, the presence of sesquiterpene lactones and flavonoids were reported in *A. tschernieviana* [23], *A. albida* [24], and *A. santolinifolia* [25], respectively. On the other hand, the bioactivities and phyto analysis of essential oils were studied for *A. kasakorum* [26], *A. lercheana* and *A. sieversiana* [27], *A. umbrosa* [28], *A. gurganica* [29], *A. proceriformis* [30], *A. terrae-albae* [31], *A. keiskeana* [32], *A. littoricola*, *A. mandshurica* [33], *A. commutata* [34], in addition to five plants belonging to the *Artemisia* species [35].

*A. commutata* [36] and *A. glauca* [37] are perennial herbs that are cultivated in northern and eastern Kazakhstan [38]. The lack of scientific records about the phytochemistry and the biological effects of these two species encouraged us to examine their phytochemical properties aiming at the isolation of promising secondary metabolites.

The WHO declared on December 28, 2021, that the confirmed cases of COVID-19 infections on a global base are 280,119,931. Sadly, 5,403,662 of them are dead [39]. In regard to these bad numbers, massive work is demanded from scientists all over the world to find a cure.

The field of computer-aided/based (in silico or computational) drug design and discovery is a fast-growing approach that has shown several successes and advancements during the last decade [40]. The structural informatics explosion allowed the identification of the exact chemical and physical properties of molecules. Consequently, the ability to compare different structures and predict a specific biological activity became much easier [41]. Also, the huge advancement in the field of proteomic enabled scientists to expect the biding and hence the activity of a molecule and target enzyme [42]. Our teamwork reported the utilization of the in silico methods to suggest a treatment that could be effective against COVID-19 in many reports [43,44,45,46,47].

We here in this manuscript, report the isolation and structure elucidation of two rare 2-phenoxychromone derivatives, 6-demethoxy-4`-O-capillarsine (**1**) and tenuflorin C (**2**) from the areal parts of *A. commutata* and *A. glauca*, respectively, for the first time. Because of the lack of research on the isolated compounds and the severe need to find a cure against COVID-19, the potential inhibitory effects of **1** and **2** against COVID-19 were investigated depending on a multiphase in silico screening method. The potentialities of **1** and **2** against COVID-19 Papain-Like Protease were confirmed according to molecular similarity, structure fingerprint, molecular docking, and molecular dynamics simulations studies. Additionally, ADMET and toxicity properties of **1** and **2** were studied to predict their likeness to be used as drugs.

## 2. Results

### 2.1. Isolation of Compounds

**Figure 1:**
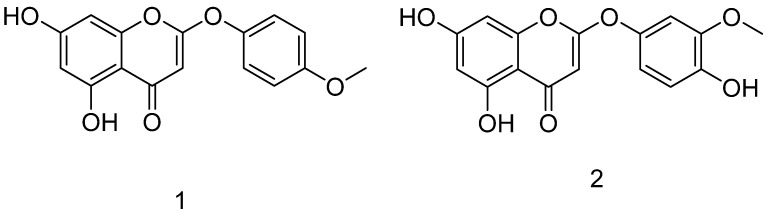
The chemical structures 6-demethoxy-4`-O-capillarsine (**1**) and tenuflorin C (**2**).

The areal parts of *A. commutata*, (940 g) and *A. glauca* (1.02 kg) were collected from the western part of the Altai Mountains. The dried plants were extracted with chloroform. The chloroform extract of *A. commutata* was subjected to chromatographic purifications steps to afford the amorphous powder of **1**. Compound **1** was identified to be 6-demethoxy-4`-O-capillarsine (Figure 1). Identification of **1** was done depending on ^1^H and ^13^C NMR (Table S1, in the Supplementary Materials), comparing the published data. Compound **1** was previously isolated from the leaves of *Mimosa tenuiflora* [48], *A. capillaris* [49], and *A. rupestris* [50]. The chloroform extract of *A. glauca* was subjected to chromatographic purification steps to give the amorphous powder of **2**. Compound **2** was identified to be 5,7,4′-trihydroxy, 3′-methoxy-2-phenoxychromone (Figure 1). Compound **2** was identified depending on ^1^H and ^13^C NMR (Table S2, in the Supplementary Materials) compared to the published data. Compound **2** was previously isolated from *Mimosa tenuiflora* [48].

### 2.2. Molecular Similarity Studies

To understand the principle of molecular similarity, we have to understand that the bioactivity of any compound is a result of some well-known interactions with a specific protein target. These protein–ligand interactions depend on specific physical and chemical interactions such as hydrogen bonding and hydrophobic interactions. Consequently, the similarity in the chemical structures of two compounds will give a similarity in the number and position of hydrogen bond donors, acceptors, and hydrophobic centers in addition to the steric configuration. This similarity could cause a noticeable similarity in the bioactivity too [51].

**Figure 2:**
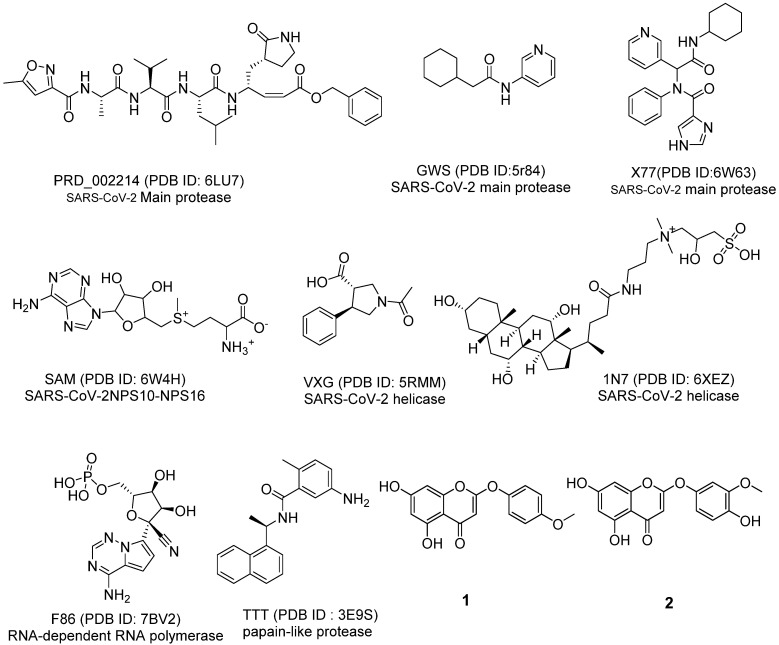
The co-crystallized ligands of coronavirus proteins and compounds **1** and **2**.

**Figure 3:**
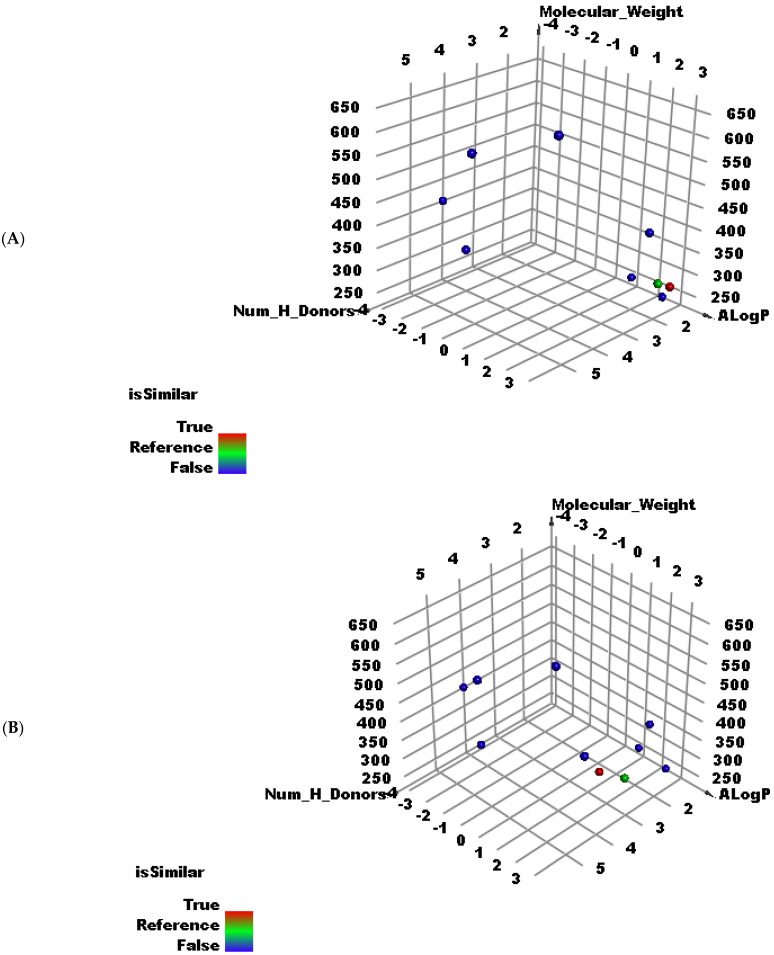
The results of similarity analysis of compound **1** (**A**), compound **2** (**B**).

To evaluate the expected anti-COVID-19 potentialities of compounds **1** and **2**, the chemical structures of them were compared with the chemical structures of eight different co-crystallized ligands of eight crucial COVID-19 proteins (Figure 2) in a structural similarity experiment. Utilizing Discovery Studio software, the following molecular properties were measured to 6-demethoxy-4`-O-capillarsine (**1**) and tenuflorin C (**2**) as well as the eight ligands; partition coefficient (ALog p) [52], molecular weight (M. W) [53], H- bond donors (HBD) [54] and acceptors (HBA) [55], rotatable bonds [56], aromatic rings [57], heterocyclic rings [58], along with the molecular fractional polar surface area (MFPSA) [59]. The results revealed the great similarity between compounds **1** and **2** and the co-crystallized ligands (**TTT**) of SARS-CoV-PLP (PDB ID: 3E9S) (Table 1, Figure 3A,B).

### 2.3. Fingerprints Studies

The molecular fingerprint is an in silico method that analyzes the similarities of two molecules or more. Molecular fingerprint explores the property profiles of a certain compound, the technique computes these properties in the forms of bits vectors examining the existence, absence, and frequencies it in the reference and target compounds.

The fingerprint approach examined the existence or absence of the these parameters: charge [60], hybridization [61], H-bond acceptor and donor [62], negative and positive ionizables [63], halogen [64], aromatic [65], and ALogP [66]. The experiment was achieved depending on Discovery Studio. The results verified the similarity of 6-demethoxy-4`-O-capillarsine (**1**), tenuflorin C (**2**), and **TTT** (Table 2).

### 2.4. Pharmacophoric Features and Flexible Alignment Studies

**Figure 4:**
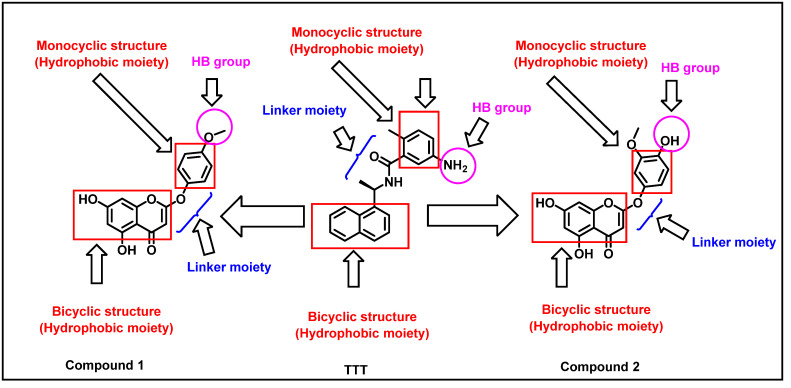
Compounds 1 and 2 have the same pharmacophoric features of the co-crystallized ligand of SARS CoV-2 PLP(TTT).

The reported main pharmacophoric features of SARS-CoV-2 Papain-Like protease inhibitors (PLPIs) are two hydrophobic systems separated be a linker [67]. As shown in Figure 4, the co-crystallized ligand (TTT) has naphthyl and phenyl groups as hydrophobic centers. The two hydrophobic groups are separated by hydrophilic linker (amide group). In addition, the terminal phenyl group is substituted by amino group as a hydrogen bonding group.

Similarly, compounds **1** and **2** have coumarin moiety as the first hydrophobic center in addition to phenyl group as the second hydrophobic moiety. In each compound, the two hydrophobic centers were separated by oxygen atom as a hydrophilic linker. This linker facilitates these compounds to have the same configuration of TTT. Additionally, the two phenyl rings were substituted by hydrophilic groups as which can form hydrogen bonds with the target receptors as TTT. From medicinal chemistry point of view, this investigation revealed that there is a great similarity between the two tested molecules and the co-crystallized ligand (TTT).

**Figure 5:**
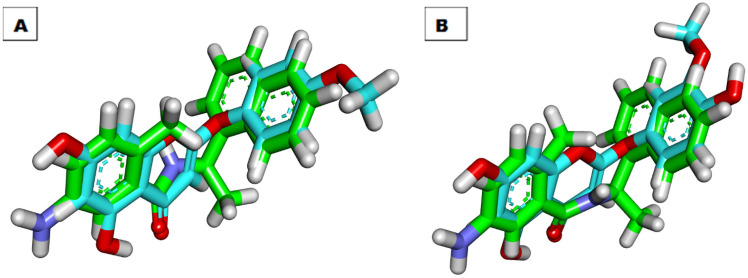
(**A**) Flexible alignment of compounds **1** (turquoise) TTT (green). (**B**) Flexible alignment of compounds **2** (turquoise) with TTT (green).

3D- Flexible alignment of the compounds **1** and **2** with TTT was presented in Figure 5. From the figure, it is possible to observe that, in general, the structure of compounds **1** and **2** have a good overlap with the reference molecule (TTT). In addition, the two tested compounds showed the same spatial orientation of TTT due to the presence of the flexible linker at the center of these compounds.

### 2.5. Docking Studies

To examine the obtained results from structural similarity and fingerprint studies, molecular docking experiments were achieved for 6-demethoxy-4`-O-capillarsine (**1**) and tenuflorin C (**2**) against PLP (PDB ID: 3E9S) using **TTT**, the co-crystallized ligand, as a reference. We considered the binding free energies (∆G) besides the binding modes as the bases of evaluation.

**Figure 6:**
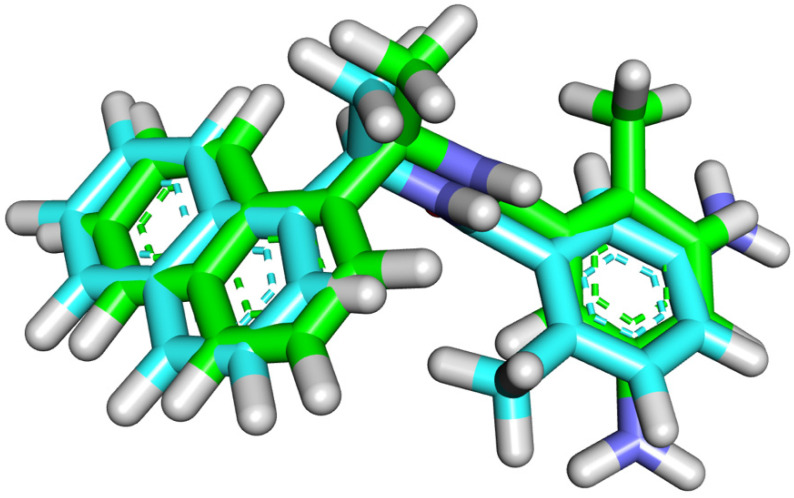
Superimposition of the co-crystallized molecule (Turquoise) and the docking pose (dark green) of the same molecule.

Firstly, a validation process has proceeded via the re-docking of **TTT** against PLP. The obtained RMSD value between the two poses was 2.1 ^ᵒ^A confirming the validity of the docking method (Figure 6).

**Figure 7:**
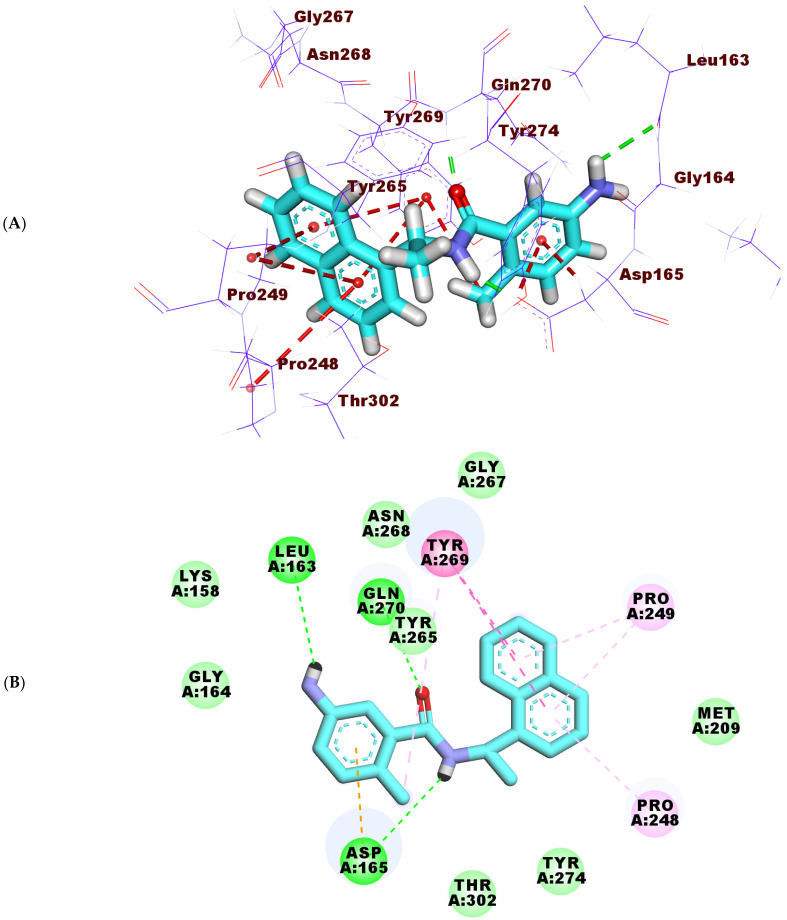

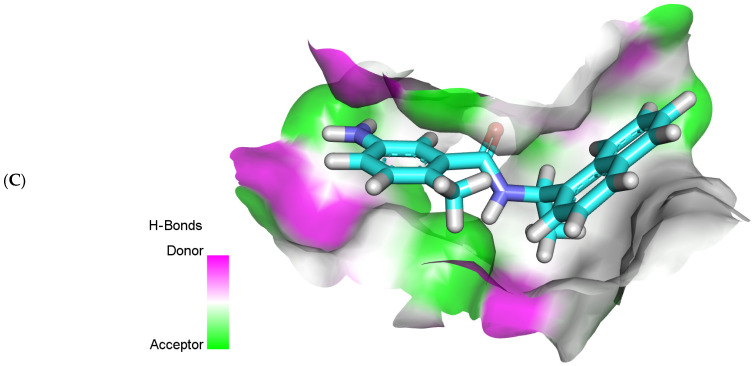
(**A**) 3D, (**B**) 2D and (**C**) Surface mapping of **TTT** inside the active site of PLP.

The binding mode of **TTT** showed a free energy of −20.32 kcal/mol. The p-toluidine part occupied the first pocket of PLP forming one H- bond with the amino acid, Leu163. The same moiety was engaged hydrophobically in two interactions with the amino acids Asp165 and Tyr269. In addition, the amide linker formed two H- bonds with the amino acids Asp165 and Gln270. The naphthalene moiety was buried in the second pocket making five hydrophobic interactions with Pro249, Pro248, and Tyr269 (Figure 7A–C).

**Figure 8:**
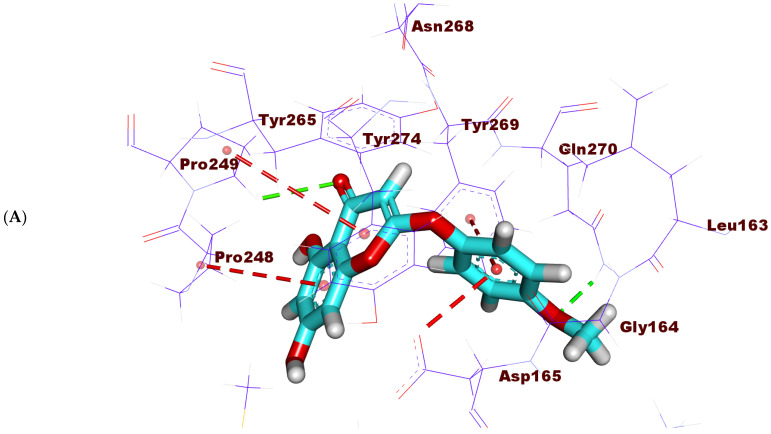

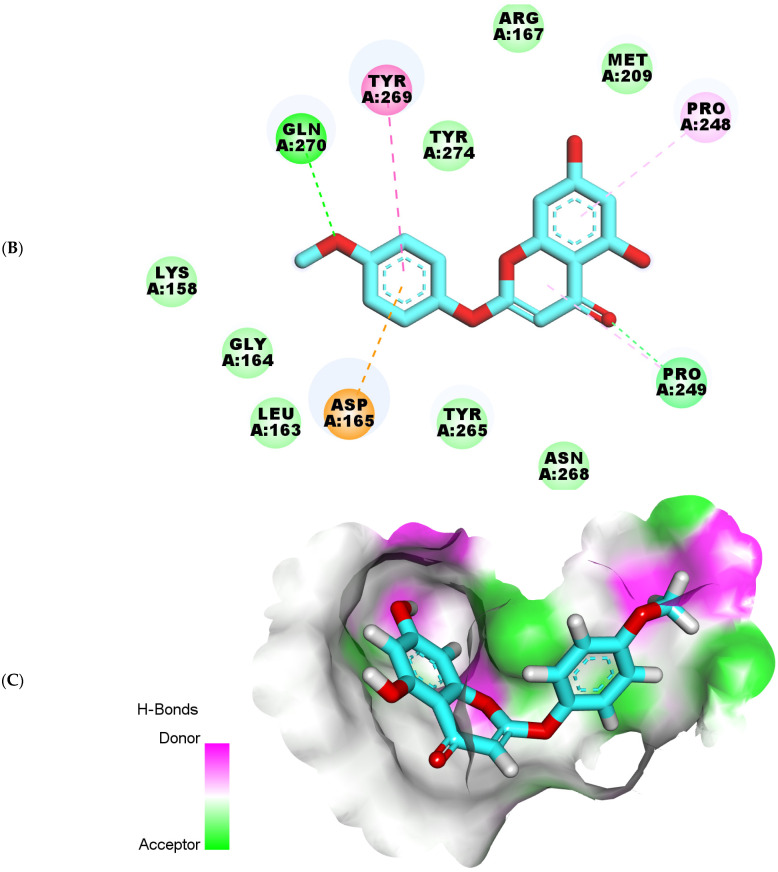
(**A**) 3D, (**B)** 2D and (**C**) Surface mapping of 6-demethoxy-4`-O-capillarsine (**1**) inside the active site of PLP.

The binding mode of 6-demethoxy-4`-O-capillarsine (**1**) exhibited a binding free energy of -18.86 kcal/mol. The anisole moiety was directed into the first pocket of PLP forming one H- bond with the amino acid, Gln270, in addition to two hydrophobic interactions with the amino acids, Tyr269 and Asp165. The 5,7-dihydroxy-4H-chromen-4-one part occupied the second pocket of PLP forming a H- bond with the amino acid Pro249 besides two hydrophobic bonds with the amino acids Pro248 and Pro249 (Figure 8A–C).

**Figure 9:**
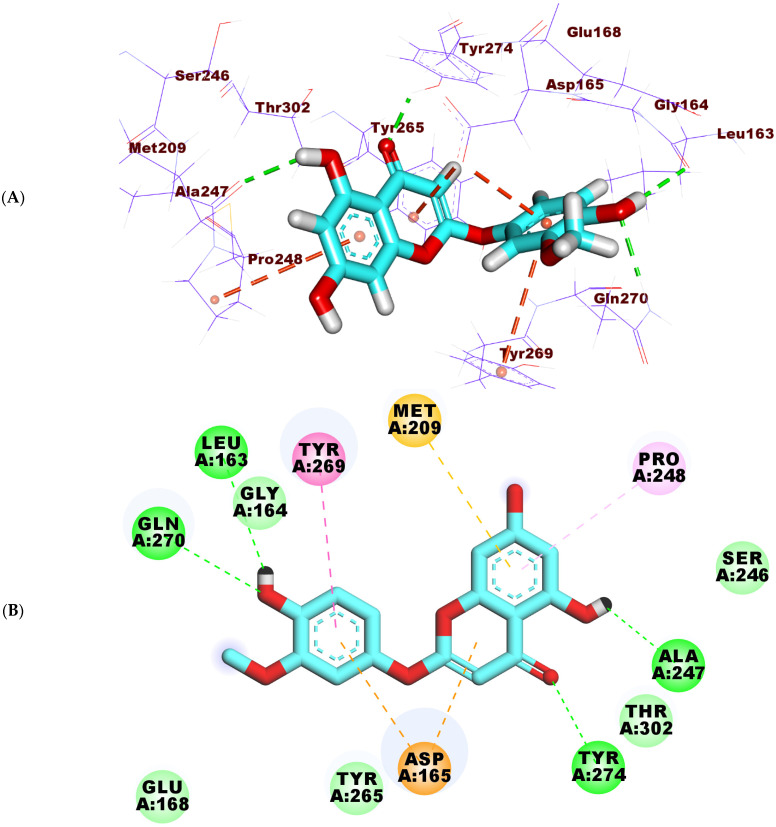

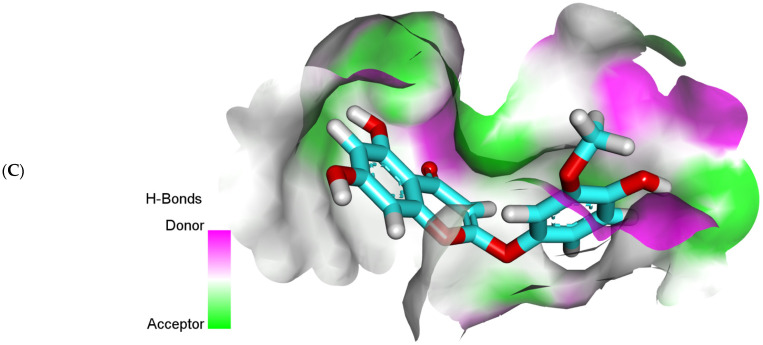
(**A**) 3D, (**B**) 2D, and (**C**) surface mapping of tenuflorin C (**2**) inside the active site of PLP.

Tenuflorin C (**2**) bonded to PLP exhibiting a binding energy of −18.37 kcal/mol. The 2-methoxyphenol part was directed into the first pocket of PLP with two H-bonds with the amino acids Gln270 and Leu163. Additionally, the same moiety was engaged hydrophobically in two interactions with the amino acids Tyr269 and Asp165. Further, the 5,7-dihydroxy-4H-chromen-4-one part occupied the second pocket of PLP forming two H- bonds with Tyr274 and Ala247. Finally, it was incorporated hydrophobically in three bonds with the amino acids Pro248, Asp165 and Met209 (Figure 9A–C).

### 2.6. In Silico ADMET Study

There is always a need to examine the ADMET properties of new molecules especially in the stage of designing to decrease the possibility of late-stage attrition. Also, the identification of ADMET properties provides good information regarding the amount and frequency of dosing as well as the toxicity [68].

**Figure 10:**
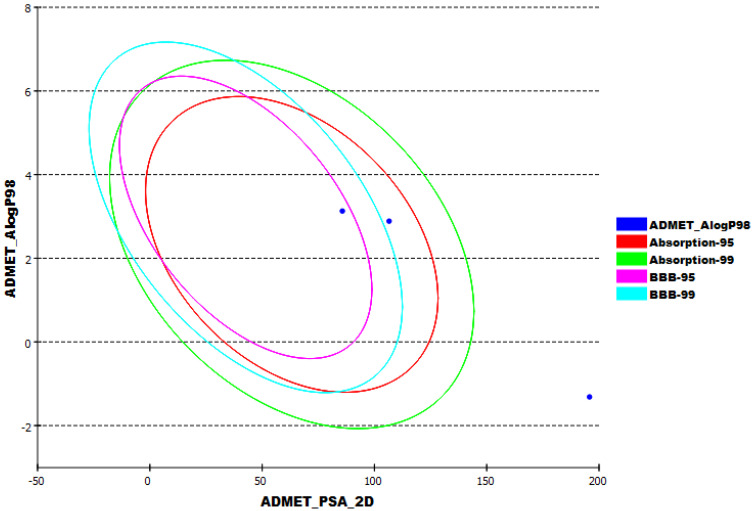
The expected ADMET study.

The in silico ADMET parameters were determined for 6-demethoxy-4`-O-capillarsine (**1**) and tenuflorin C (**2**) using Discovery Studio software and utilizing Remdesivir as a reference. The results were illustrated in Figure 10 and Table 3.

The results revealed that 6-demethoxy-4`-O-capillarsine (**1**) and tenuflorin C (**2**) have low and very low chance to penetrate BBB, respectively. Further, 6-demethoxy-4`-O-capillarsine (**1**) and tenuflorin C (**2**) exhibited good aqueous solubility and intestinal absorption. Finally, 6-demethoxy-4`-O-capillarsine (**1**) and tenuflorin C (**2**) were predicted to be a CYP2D6 non-inhibitor and to be able to bind plasma protein by a ratio of more than 90%.

### 2.7. In Silico Toxicity Studies

The predicted toxicity levels of 6-demethoxy-4`-O-capillarsine (**1**) and tenuflorin C (**2**) were investigated in silico using Discovery Studio software against seven toxicity models. Remdesivir was used as a reference. As shown in Table 4, 6-demethoxy-4`-O-capillarsine (**1**) and tenuflorin C (**2**) were predicted to be non-carcinogenic and have high TD_50_ values of 144.939 and 113.277 mg/kg/day, respectively. The values were more than 12-fold that of remdesivir (9.2458 mg/kg/day). Further, 6-demethoxy-4`-O-capillarsine (**1**) and tenuflorin C (**2**) showed high rat maximum tolerated dose (MTD) values of 0.289735 and 0.44318 g/kg, respectively, compared to remdesivir (0.234965 g/kg). The rat oral LD_50_ values for **1** and **2** were 0.363122 and 0.549081 g/kg, respectively, and regarding the rat chronic LOAEL model, their values were 0.0221904 and 0.0362493, respectively. Finally, 6-demethoxy-4`-O-capillarsine (**1**) and tenuflorin C (**2**) were predicted to have mild ocular irritancy and to have no irritancy against the skin model.

### 2.8. Molecular Dynamics (MD) Simulation Studies

The main advantage of molecular dynamics (MD) simulation is its ability to recognize the flexibility of the protein–ligand complex. This advantage enables the MD studies to estimate accurately the thermodynamics and kinetics that take place during the drug–enzyme binding [69].

MD simulations can explore the dynamic structural information of the protein–ligand complex and provide plentiful information on the energetic changes that resulted from the interactions between protein and ligand. Such information could be very useful to understand the structure–function relationship of the protein–ligand complex interactions [70]. In order to authenticate the binding and to investigate the thermodynamic characters of the isolated compounds, molecular dynamics (MD) simulation studies were conducted to 6-demethoxy-4`-O-capillarsine, **1**, as it showed a bitter binding energy, against PLP.

**Figure 11:**
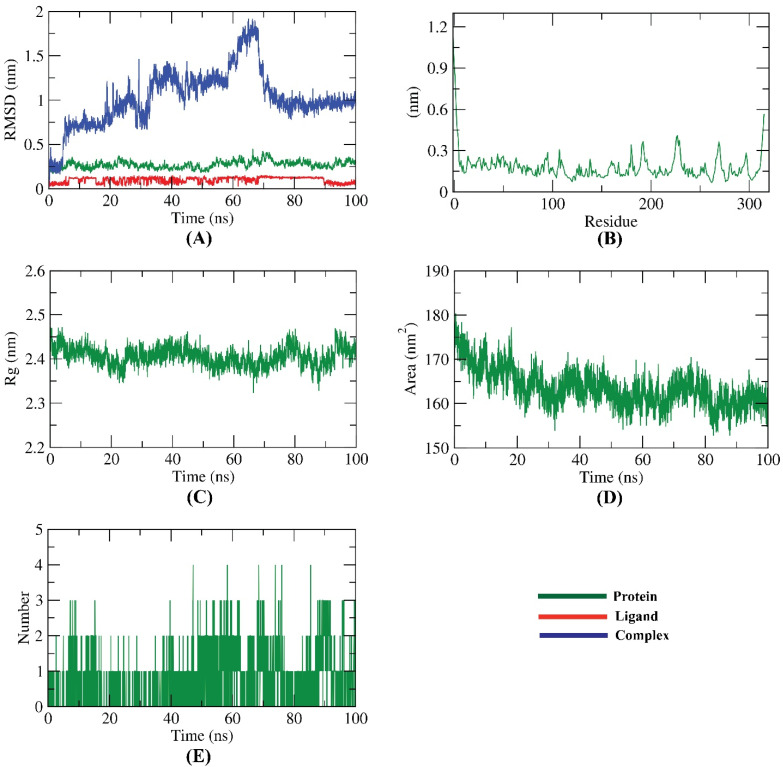
Molecular dynamics simulations results; (**A**) RMSD values of 6-demethoxy-4`-O-capillarsine, PLP and 6-demethoxy-4`-O-capillarsine -PLP complex during MD runs. (**B**) RMSF for PLP in the MD run. (**C**) Radius of gyration of PLP in the MD run. (**D**) SASA of PLP in the MD run. (**E**) H- bonding between 6-demethoxy-4`-O-capillarsine -PLP complex in the MD run.

The atomical dynamic movements and conformational variations of backbone atoms of the 6-demethoxy-4`-O-capillarsine -PLP complex were calculated by RMSD to detect their stability upon ligand bonded and apo states. It was observed that the protein and ligand exhibit lower RMSD with no major fluctuations indicating their greater stability. The 6-demethoxy-4`-O-capillarsine -PLP complex was slightly fluctuating till 70 ns~ and stabled later (Figure 11A). The flexibility of PLP was evaluated in terms of RMSF (Figure 11B) to get more information about the protein regions that had been fluctuating during the simulation. It can be understood that the binding of 6-demethoxy-4`-O-capillarsine does not make PLP very flexible. The compactness of the 6-demethoxy-4`-O-capillarsine -PLP complex was evaluated through the examination of the radius of gyration (Rg) (Figure 11C). Decreasing fluctuation during the simulation study is an indication of the higher compactness of the system. The Rg of the 6-demethoxy-4`-O-capillarsine -PLP complex was found to be similar throughout the simulation period. Interaction between 6-demethoxy-4`-O-capillarsine -PLP complexes and solvents was evaluated through the calculation of solvent accessible surface area (SASA) over the simulation period. So, SASA of the 6-demethoxy-4`-O-capillarsine -PLP complex was calculated to analyze the extent of the conformational changes that occurred during the interaction. Interestingly, PLP featured a reduction of the surface area showing a relatively lower SASA value than the starting period (Figure 11D). Hydrogen bonding in the 6-demethoxy-4`-O-capillarsine -PLP complex is essential to stabilize the structure. It was noticed that the highest number of conformations of the PLP formed up to three H- bonds with the 6-demethoxy-4`-O-capillarsine (Figure 11E).

## 3. Experimental

### 3.1. Isolation and Structure Elucidation of Compounds

A total of 0.94 kg of *Artemisia commutata* and 1.04 kg of *Artemisia glauca* areal parts were extracted with chloroform. Successive chromatographic methods led to the isolation of compounds (**1** and **2**). Detailed description in the method part in Supplementary Materials.

### 3.2. Molecular Similarity

Molecular Similarity of 6-demethoxy-4`-O-capillarsine (**1**), tenuflorin C (**2**) against the co-crystallized ligands of SARS-CoV-2 was carried out calculated using Discovery Studio 4.0 (See the method part in Supplementary Materials).

### 3.3. Fingerprint Study

A fingerprint study of 6-demethoxy-4`-O-capillarsine (**1**), tenuflorin C (**2**) against the eight co-crystallized ligands of SARS-CoV-2 was carried out calculated using Discovery Studio 4.0. (See Supplementary Materials)

### 3.4. DFT

The DFT parameters were calculated for 6-demethoxy-4`-O-capillarsine (**1**), tenuflorin C (**2**) using Discovery Studio software [71]. (See the method part in Supplementary Materials).

### 3.5. Docking Studies

The docking investigation was carried out for 6-demethoxy-4`-O-capillarsine (**1**), tenuflorin C (**2**) using MOE2014 software and Discovery Studio 4.0 [72,73,74] (See the method part in Supplementary Materials).

### 3.6. ADMET

ADMET descriptors of 6-demethoxy-4`-O-capillarsine (**1**), tenuflorin C (**2**) were determined using Discovery Studio 4.0. [75,76] (See the method part in Supplementary Materials).

### 3.7. Toxicity Studies

Seven toxicity parameters of 6-demethoxy-4`-O-capillarsine (**1**), tenuflorin C (**2**) were calculated using Discovery Studio 4.0 [77,78,79] (See the method part in Supplementary Materials).

### 3.8. Molecular Dynamics Simulations

The system was prepared using the web-based CHARMM-GUI [80,81,82] interface utilizing CHARMM36 force field [83] and NAMD 2.13 [84] package. The TIP3P explicit solvation model was used (See the method part in Supplementary Materials).

## Figures and Tables

**Table 1:** Structural properties of compounds **1**, **2** and **TTT**.

Compound	ALog p	M. W	HBA	HBD	Rotatable Bonds	Rings	Aromatic Rings	MFPSA	Minimum Distance
**1**	3.129	300.263	6	2	3	3	2	0.302	0
**2**	2.887	316.262	7	3	3	3	2	0.359	0
**TTT**	3.647	304.386	2	2	3	3	3	0.171	0.723

**Table 2:** Fingerprint similarity between compounds **1**, **2** and **TTT**.

Compound	Similarity	SA	SB	SC
**1**	1	299	0	0
**2**	1	289	0	0
**TTT**	0.579	276	178	23

SA: The number bits present in both the isolated 2-phenoxychromone derivative and **TTT.** SB: The number of bits in the isolated 2-phenoxychromone derivative but not **TTT.** SC: The number of bits in **TTT** but not the isolated 2-phenoxychromone derivative.

**Table 3:** Binding free energies (∆G in Kcal/mol) of the 6-demethoxy-4`-O-capillarsine (**1**), tenuflorin C (**2**) and **TTT** against PLP.

Compound	∆G [Kcal/mol]
**1**	−18.86
**2**	−18.37
**TTT**	−20.32

**Table 4:** Toxicity properties of compounds.

Compound	FDA Rodent Carcinogenicity	Carcinogenicity TD_50_^a^	Rat MTD ^b^	Rat Oral LD_50_^b^	LOAEL ^b^	Ocular Irritancy ^c^	Skin Irritancy ^c^
**1**	Not a carcinogen	144.939	0.289735	0.363122	0.0221904	+	-
**2**	Not a carcinogen	113.277	0.44318	0.549081	0.0362493	+	-
remdesivir	Not a carcinogen	9.2458	0.234965	0.308859	0.0037911	+	+

^a^ Unit: mg·kg^−1^·day^−1.^, ^b^ Unit: g·kg^−1.^, ^c^ - = nonirritant, + = mild.

## Data Availability

Data is contained within the article.

## Supplementary Materials

The following are available online.
