# Supplementary material for: Isolation and In Silico Anti-SARS-CoV-2 Papain-Like Protease Potentialities of Two Rare 2-Phenoxychromone Derivatives from Artemisia spp
Source: Molecules. 2022 Feb 11;27(4):1216. doi: 10.3390/molecules27041216 (PMC8879996; doi:10.3390/molecules27041216)
Supplement: Supplementary file 1 [file molecules-27-01216-s001.zip › molecules-1565726 - supplementary revised.pdf]

Supporting Data
Isolation and *in silico* anti-SARS-Cov-2 Papain like Protease potentialities of two rare 2-phenoxychromone derivatives from *Artemisia* spp.

Yerlan M. Suleimen[1,2], Rani A. Jose[3], Raigul N. Suleimen[4*], Christoph Arenz[5], Margarita Yu. Ishmuratova[6], Toppet Suzanne[3], Wim Dehaen[3], Aisha A. Alsfouk[7], Eslam B. Elkaeed[8] Ibrahim. H. Eissa[9], Ahmed M. Metwaly[10,11*]

[1]The International Centre for Interdisciplinary Solutions on Antibiotics and Secondary Metabolites, Republican collection of microorganisms, Nur-Sultan, Republic of Kazakhstan

[2]The laboratory of Engineering Profile of NMR Spectroscopy, Sh. Ualikhanov Kokshetau University, Kokshetau, Republic of Kazakhstan

[3]Catholic University of Leuven, Leuven, Belgium

[4]L.N. Gumilyov Eurasian National University, 010010, Nur-Sultan, Kazakstan

[5]Institut für Chemie der Humboldt-Universität zu Berlin, Germany

[6]Department of Botany, E.A. Buketov Karaganda University, Karaganda, Republic of Kazakhstan

[7]Department of Pharmaceutical Sciences, College of Pharmacy, Princess Nourah bint Abdulrahman University, P.O. Box 84428, Riyadh 11671, Saudi Arabia

[8]Department of Pharmaceutical Sciences, College of Pharmacy, AlMaarefa University, Ad Diriyah 13713, Riyadh, Saudi Arabia

[9]Pharmaceutical Medicinal Chemistry & Drug Design Department, Faculty of Pharmacy (Boys), Al-Azhar University, Cairo11884, Egypt

[10]Pharmacognosy and Medicinal Plants Department, Faculty of Pharmacy (Boys), Al-Azhar University, Cairo, Egypt

[11]Biopharmaceutical Product Research Department, Genetic Engineering and Biotechnology Research Institute, City of Scientific Research and Technological Applications, Alexandria, Egypt

Content

Table S.1 - ^{1}H and ^{13}C spectral data of compound **1** (DMSO, δ)
Table S.2 - ^{1}H and ^{13}C spectral data of **2** (DMSO, δ)
^{1}H and ^{13}C spectral data of compound **1** (DMSO, δ)
^{1}H and ^{13}C, DEPT, HMQC and HMBC spectral data of compound **2** (DMSO, δ)
Method
Toxicity report

Table S.1 - ^{1}H and ^{13}C spectral data **1** (DMSO, δ)

Position	δH (*J*=Hz)	δ C	Position	δH (*J*=Hz)	δ C
2		167.77	10		101.97
3	5.03 (s)	86.77	1`		144.36
4		183.09	2`, 6`	7.31 (d, 10.0)	121.63
5		161.43	3`, 5`	7.07 (d, 10.0)	115.09
6	6.34 (d, 2.2)	99.74	4`		157.80
7		163.93	4`-OMe	3.79 (s)	55.73
8	6.20 (d, 2.2)	93.63	5-OH	12.78 (s)	
9		154.97	7-OH	10.88 (s)	

Table S.2 - ^{1}H and ^{13}C spectral data **2** (DMSO, δ)

Position	δH (*J*=Hz)	δ C	Position	δH (*J*=Hz)	δ C
2	-	167.94	2’	6.87 d (3.2)	105.28
3	5.06 s	86.93	3’	-	148.53
4	-	183.06	4’	-	145.32
5	-	154.89	5’	7.02 d (8.5)	115.19
6	6.35 d (2)	93.52	6’	6.76 dd (8.5, 3.2)	112.09
7	-	101.92	4’-OH	9.37 s	
8	6.19 d (2)	98.89	5-OH	12.82 s	
9	-	161.36	7-OH	10.87 s	
10	-	163.86	2’-OMe	3.78 s	55.92

Table. S.3. Predicted ADMET for the designed compounds and reference drug

Comp.	BBB level [a]	Solubility level [b]	Absorption level [c]	CYP2D6 prediction [d]	PPB prediction [e]
Flavonoid-1	3	3	0	false	true
Flavonoid-2	4	3	0	false	true
remdesivir	4	3	3	false	false

[a] BBB level, blood brain barrier level, 0 = very high, 1 = high, 2 = medium, 3 = low, 4 = very low.

[b] Solubility level, 1 = very low, 2 = low, 3 = good, 4 = optimal.

[c] Absorption level, 0 = good, 1 = moderate, 2 = poor, 3 = very poor.

[d] CYP2D6, cytochrome P2D6, TRUE = inhibitor, FALSE = non inhibitor.

[E] PBB, plasma protein binding, FALSE means less than 90%, TRUE means more than 90%

^{1}H and ^{13}C spectral data of compound **1** (DMSO)

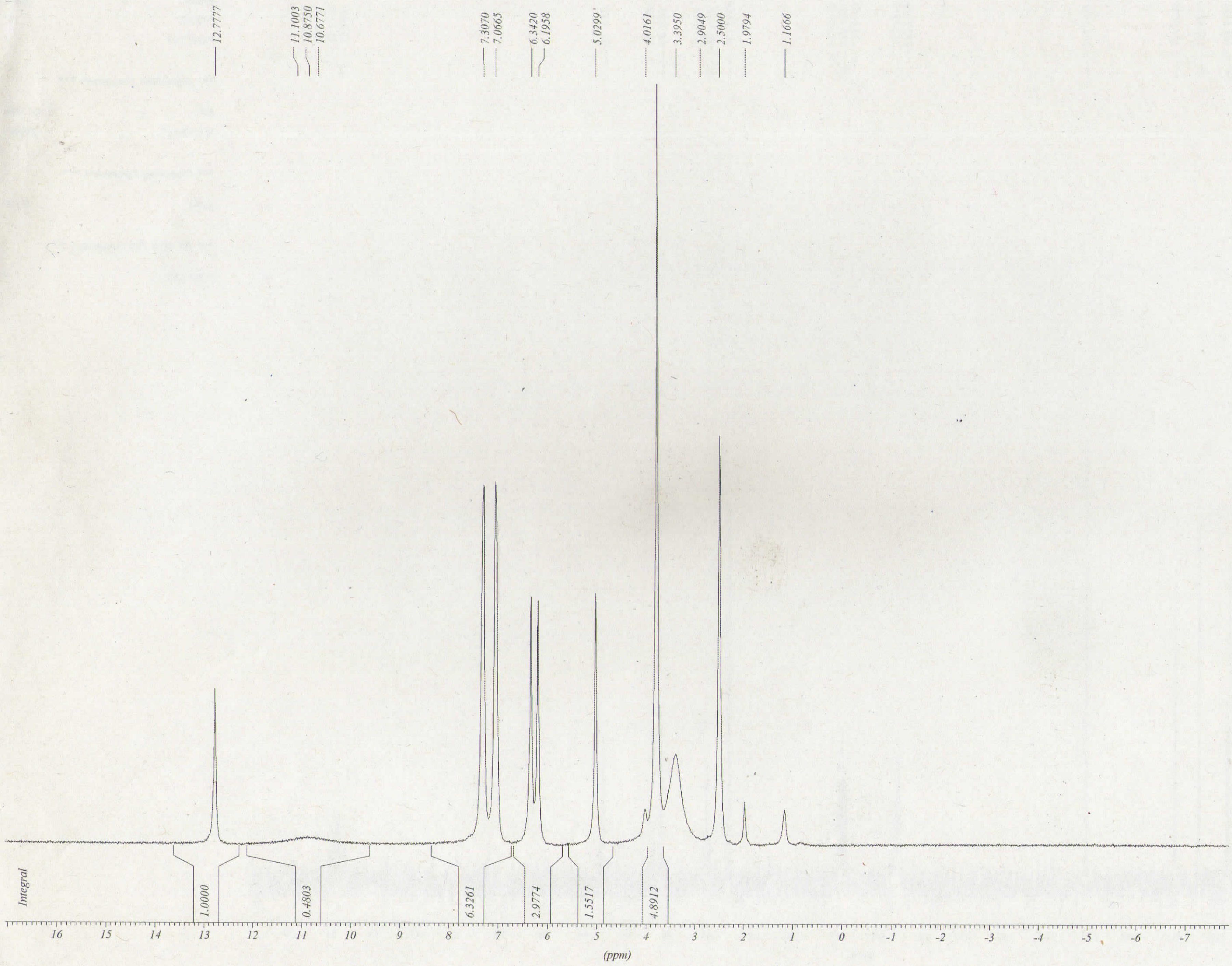

*** Current Data Parameters ***

NAME	:	acb-2
EXPNO	:	10
PROCNO	:	1

*** Acquisition Parameters ***

BF1	:	300.1300000
SOLVENT	:	DMSO

*** Processing Parameters ***

AZFE	:	0.100

*** 1D NMR Plot Parameters ***

SOLVENT	:	?

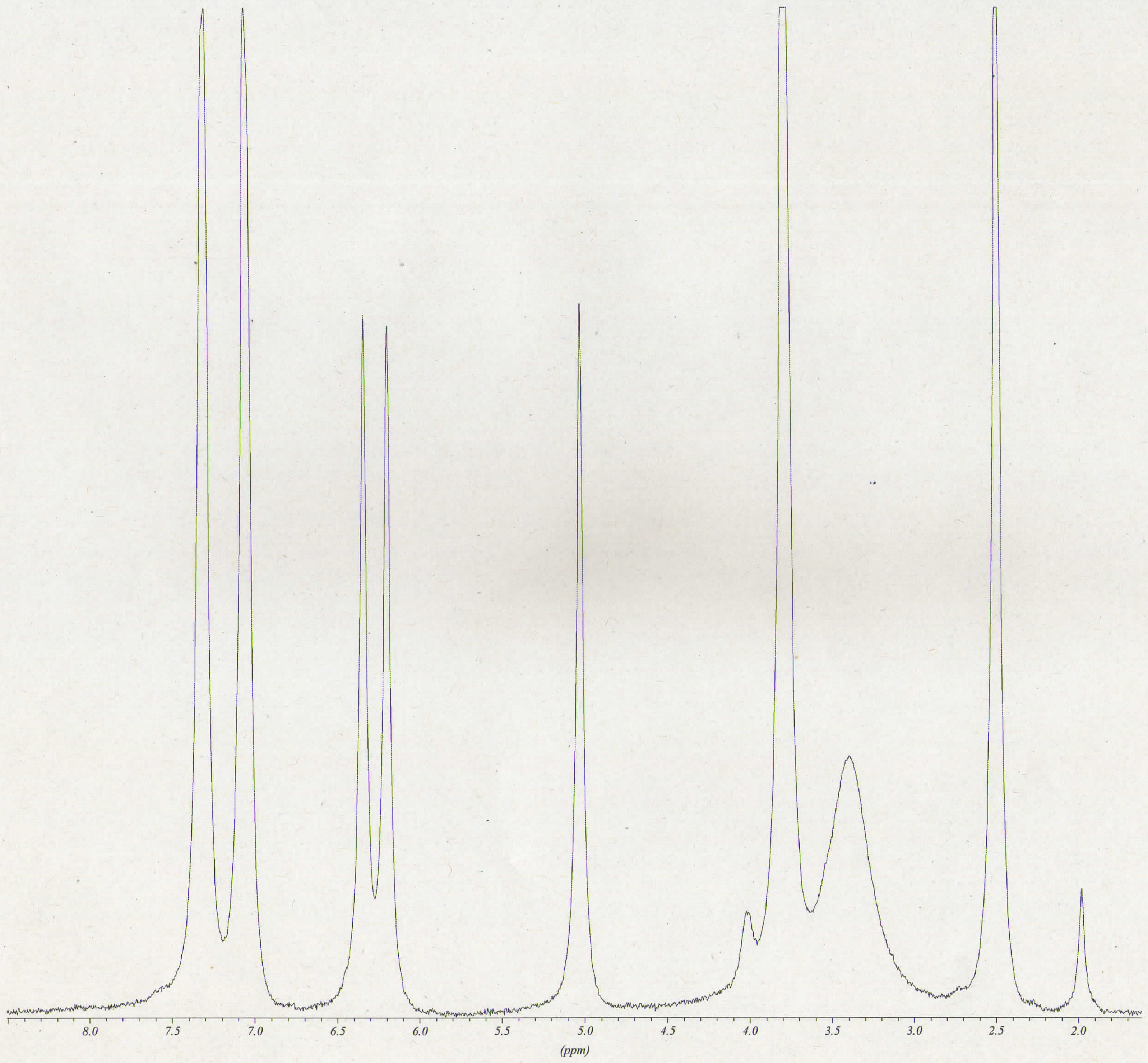

*** Current Data Parameters ***

NAME	:	acb-2
EXPNO	:	10
PROCNO	:	1

*** Acquisition Parameters ***

BF1	:	300.1300000 MH
SOLVENT	:	DMSO

*** Processing Parameters ***

AZFE	:	0.100 ppn

*** 1D NMR Plot Parameters ***

SOLVENT	:	?

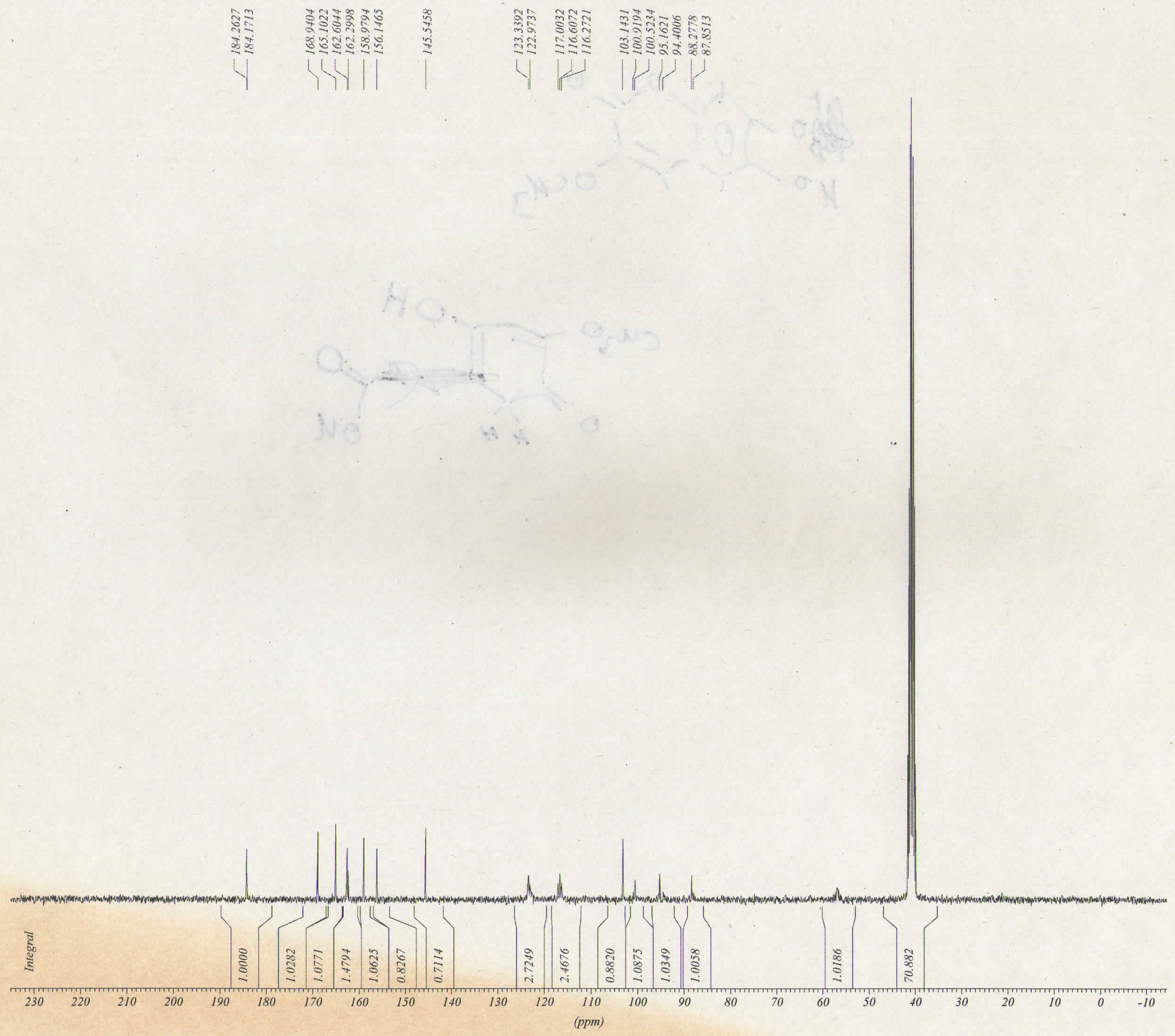

*** Current Data Parameters ***

NAME	:	acb-2
EXPNO	:	11
PROCNO	:	1

*** Acquisition Parameters ***

BF1	:	75.4677190 MH
SOLVENT	:	DMSO

*** Processing Parameters ***

AZFE	:	0.100 ppn

*** 1D NMR Plot Parameters ***

SOLVENT	:	?

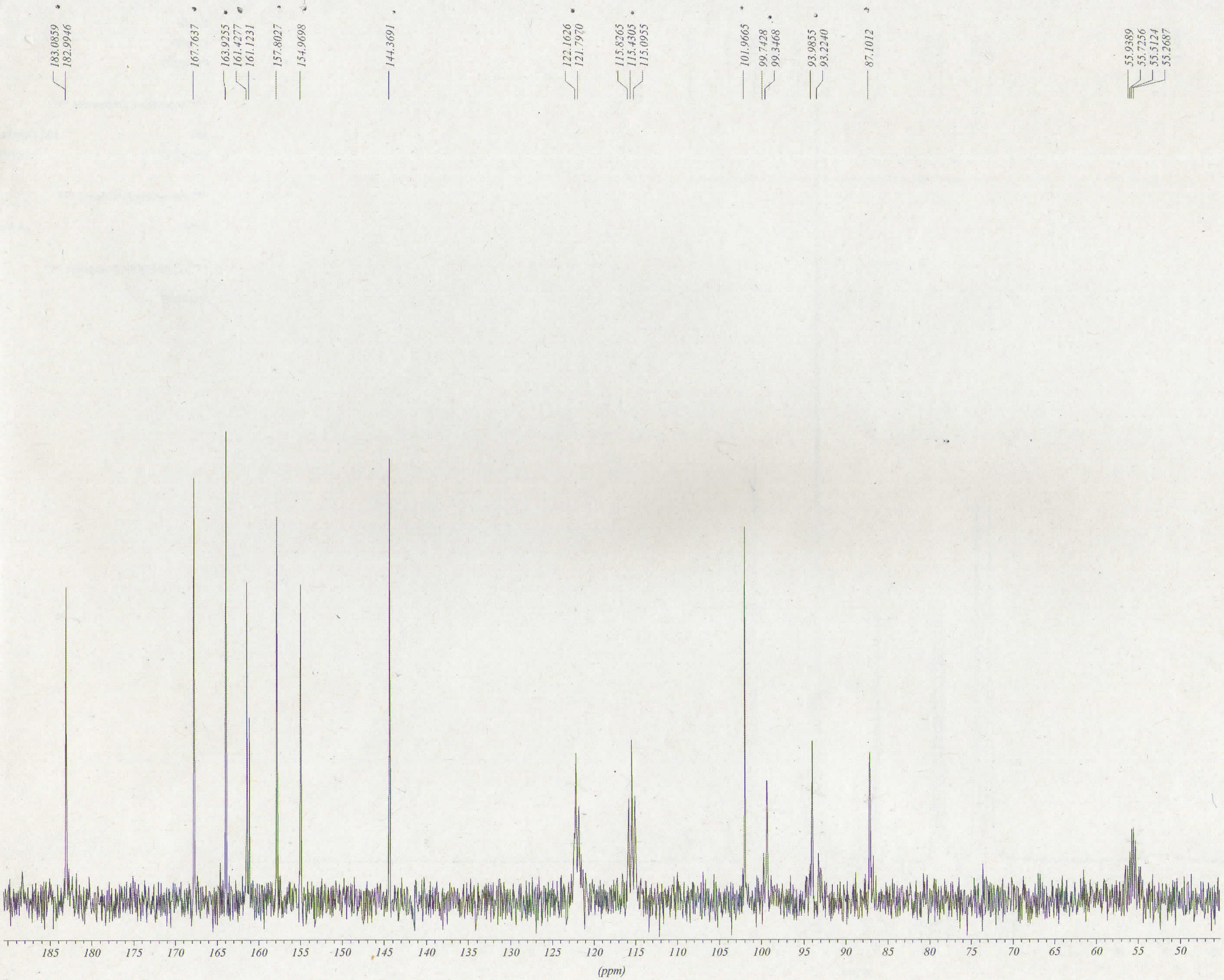

*** Current Data Parameters ***

NAME	:	acb-2
EXPNO	:	11
PROCNO	:	1

*** Acquisition Parameters ***

BF1	:	75.4677190
SOLVENT	:	DMSO

*** Processing Parameters ***

AZFE	:	0.100

*** 1D NMR Plot Parameters ***

SOLVENT	:	?

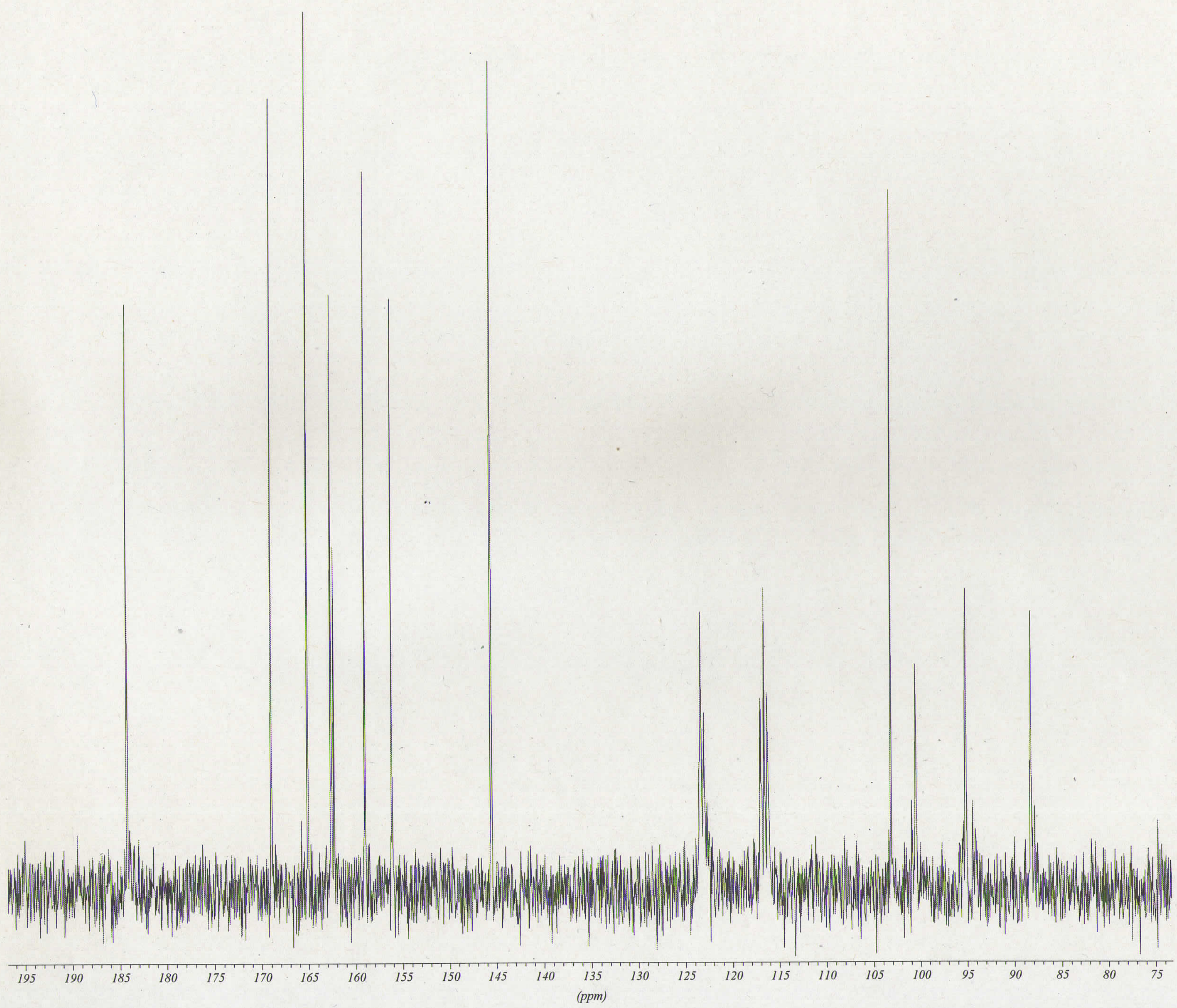

*** Current Data Parameters ***

NAME	:	acb-2
EXPNO	:	11
PROCNO	:	1

*** Acquisition Parameters ***

BF1	:	75.4677190 MH
SOLVENT	:	DMSO

*** Processing Parameters ***

AZFE	:	0.100 ppn

*** 1D NMR Plot Parameters ***

SOLVENT	:	?

^{1}H and ^{13}C, DEPT, HMQC and HMBC spectral data of compound **2** (DMSO)

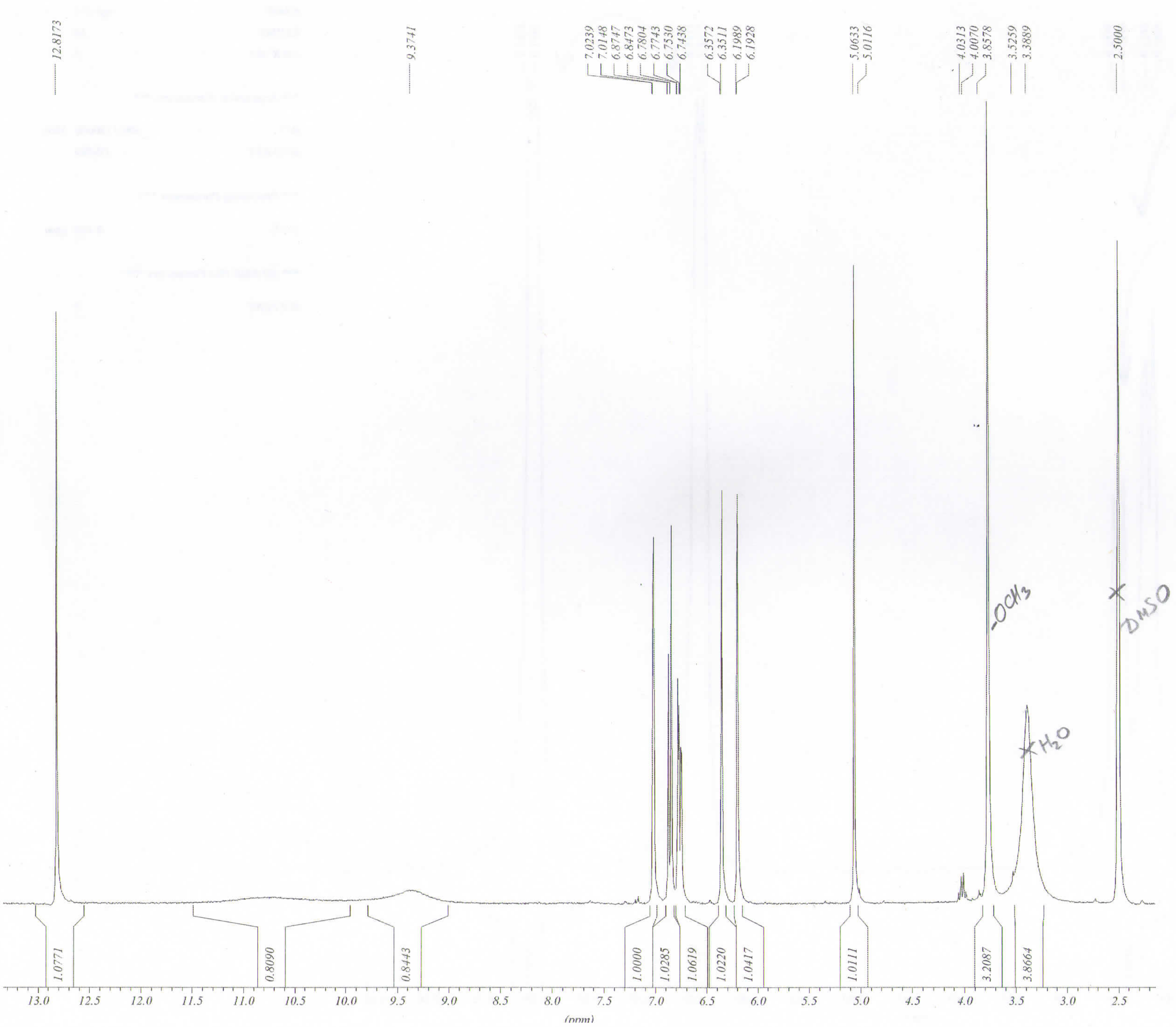

*** Current Data Parameters ***

NAME	:	agb-2-1
EXPNO	:	10
PROCNO	:	1

*** Acquisition Parameters ***

BF1	:	300.1300000 MHz
SOLVENT	:	DMSO

*** Processing Parameters ***

AZFE	:	0.100 ppm

*** 1D NMR Plot Parameters ***

SOLVENT	:	?

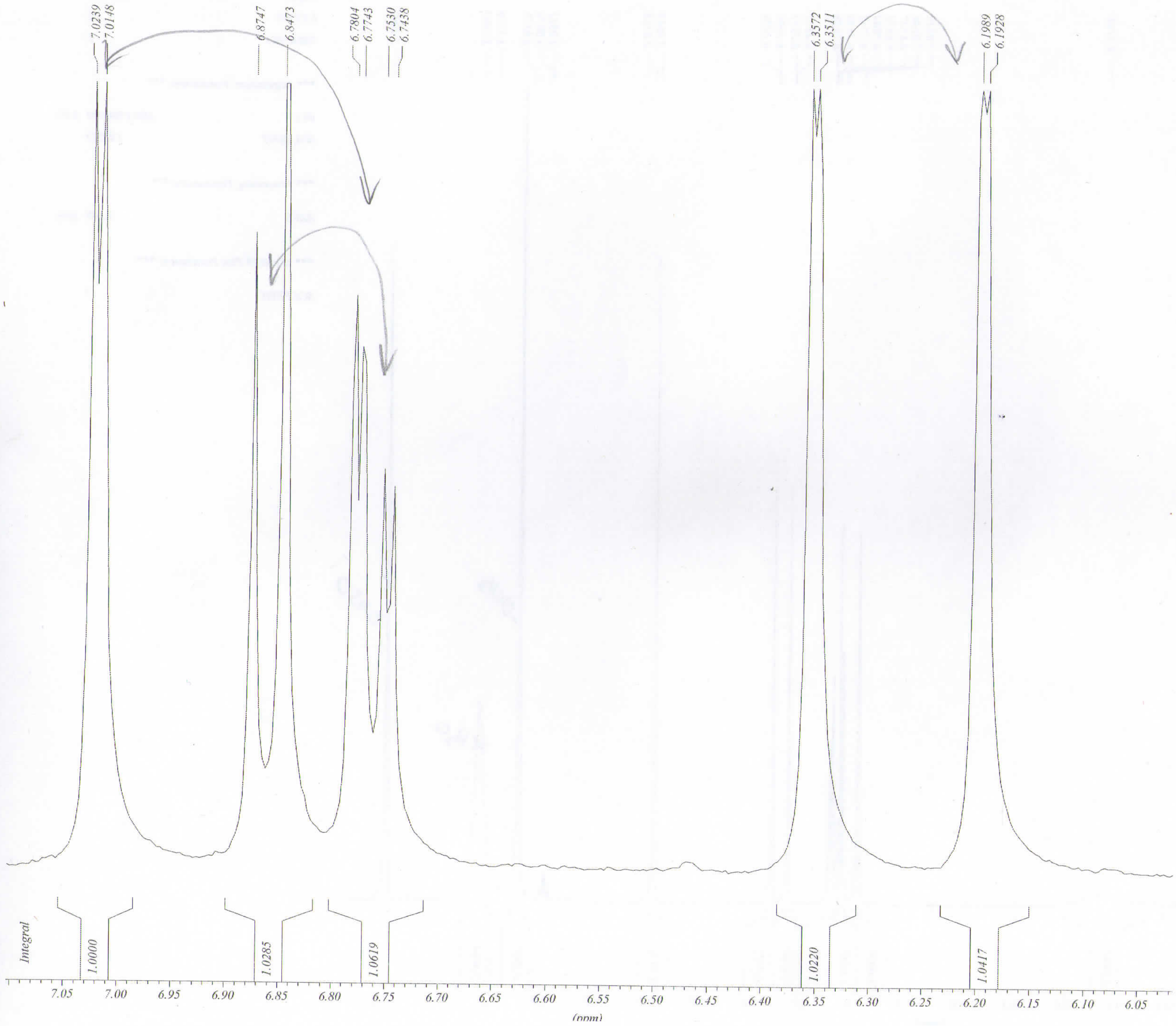

*** Current Data Parameters ***

NAME	:	agb-2-1
EXPNO	:	10
PROCNO	:	1

*** Acquisition Parameters ***

BF1	:	300.1300000 MHz
SOLVENT	:	DMSO

*** Processing Parameters ***

AZFE	:	0.100 ppm

*** 1D NMR Plot Parameters ***

SOLVENT	:	?

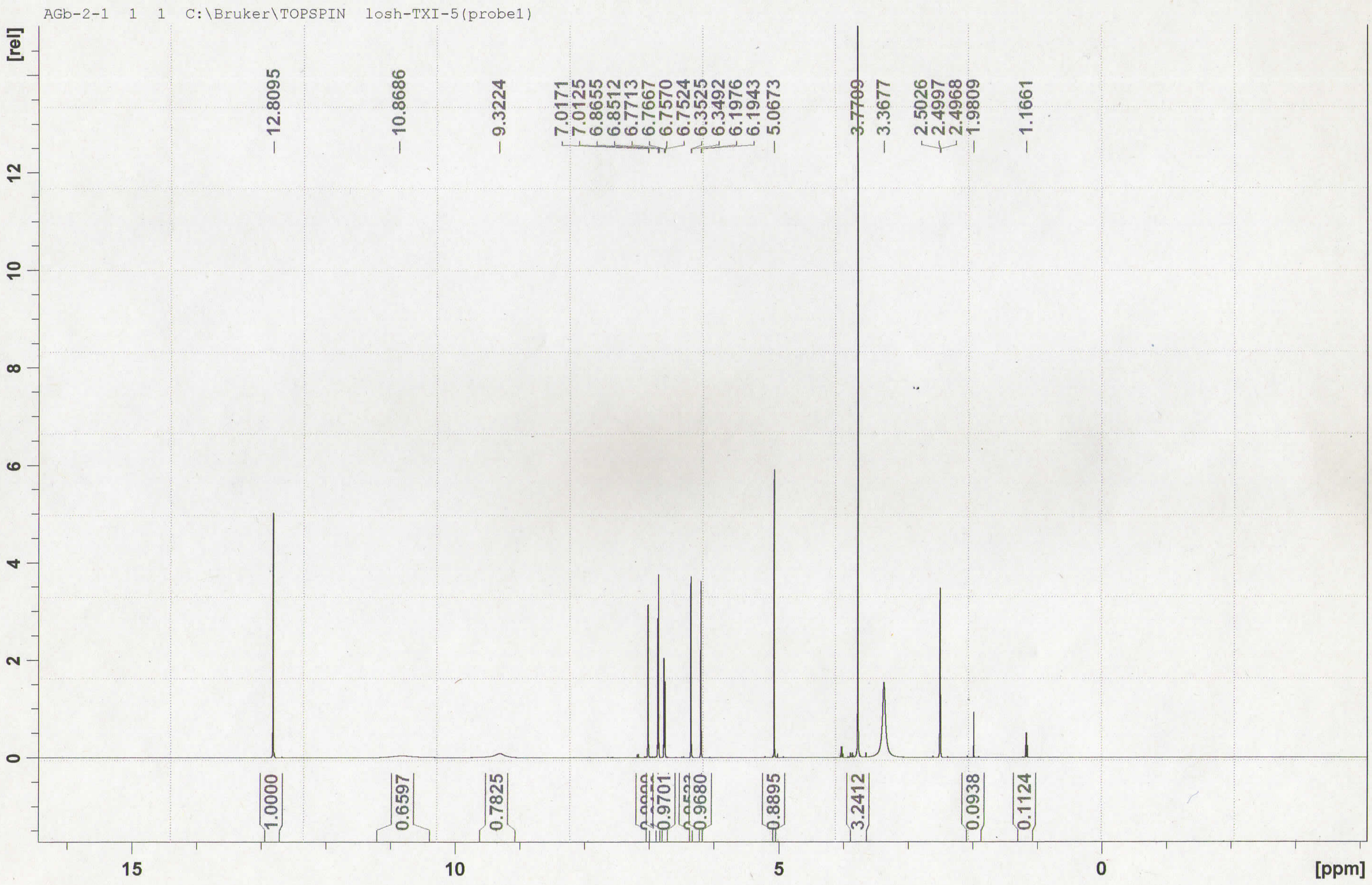
AGb-2-1 1 1 C:\Bruker\TOPSPIN losh-TXI-5(probe1)
12.8095
10.8686
9.3224
7.0171
7.0125
6.8655
6.8512
6.7713
6.7667
6.7570
6.7524
6.3525
6.3492
6.1976
6.1943
5.0673
3.7709
3.3677
2.5026
2.4997
2.4968
1.9809
1.1661
1.0000
0.6597
0.7825
0.9701
0.9680
0.8895
3.2412
0.0938
0.1124
[rel]
[ppm]

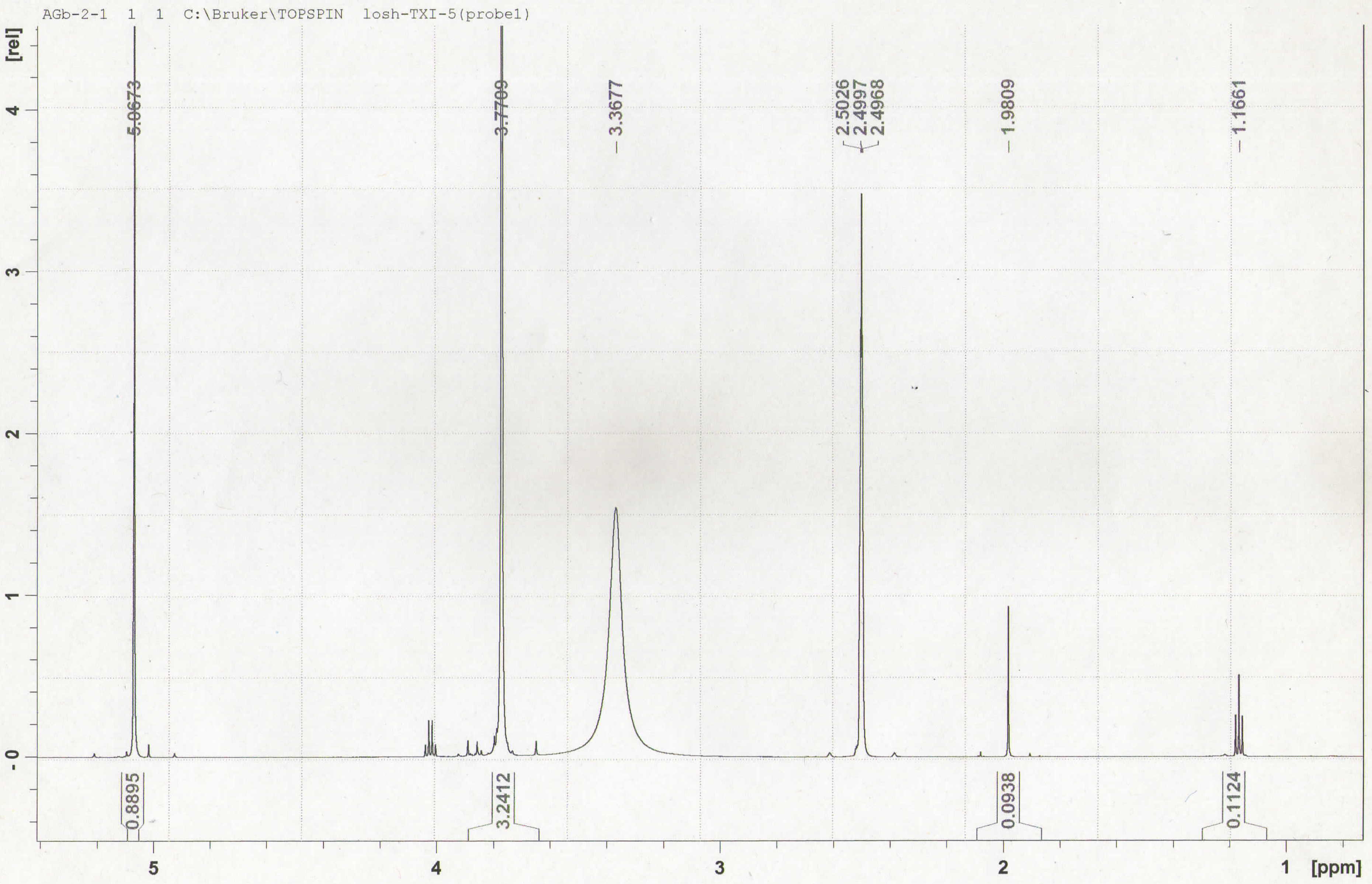
AGb-2-1 1 1 C:\Bruker\TOPSPIN losh-TXI-5(probe1)
[rel]
4
3
2
1
-0
5.0673
3.7709
3.3677
2.5026
2.4997
2.4968
1.9809
1.1661
0.8895
3.2412
0.0938
0.1124
5
4
3
2
1
[ppm]

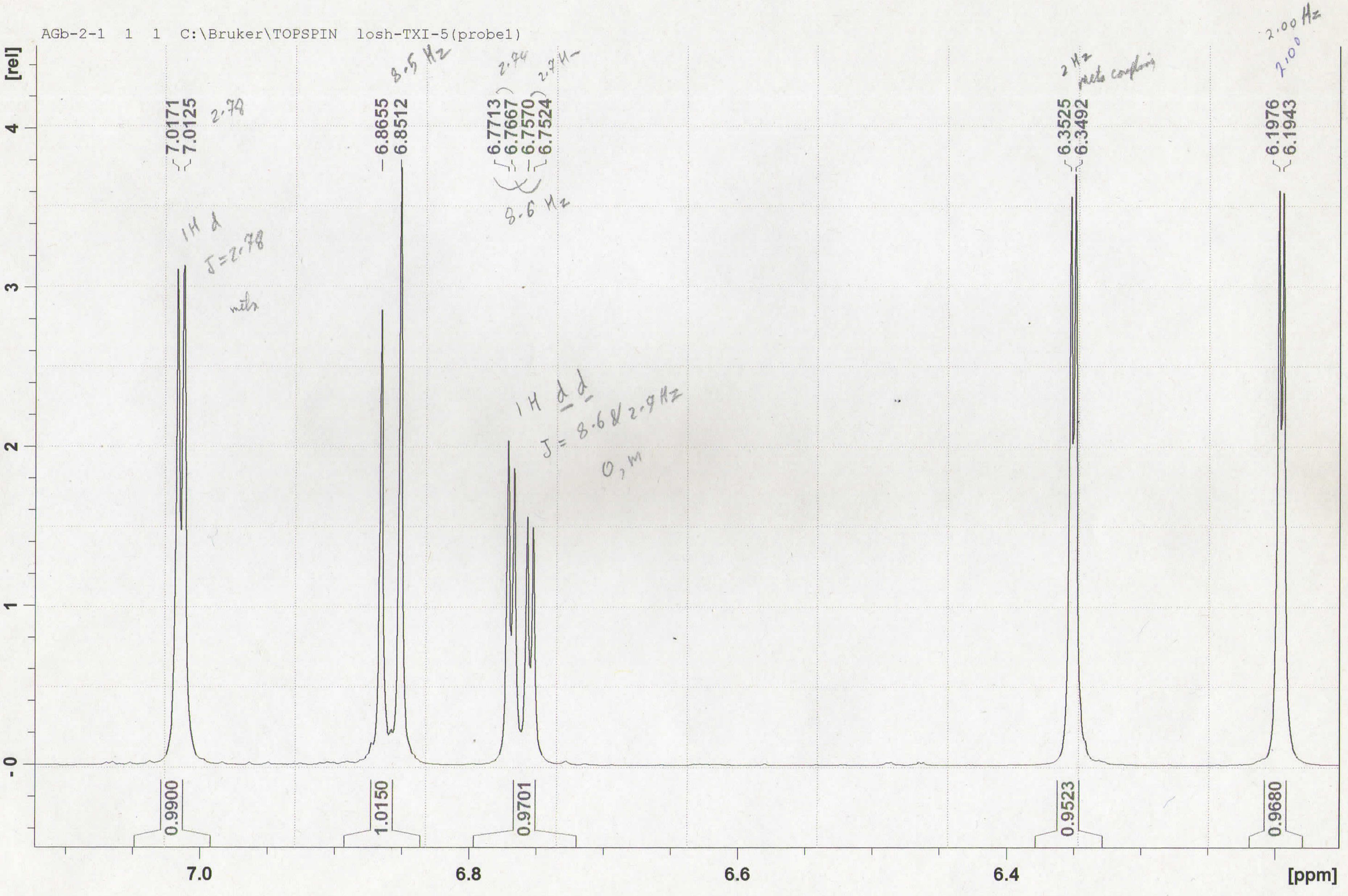
AGb-2-1 1 1 C:\Bruker\TOPSPIN losh-TXI-5(probe1)
7.0171
7.0125
6.8655
6.8512
6.7713
6.7667
6.7570
6.7524
6.3525
6.3492
6.1976
6.1943
0.9900
1.0150
0.9701
0.9523
0.9680
[rel]
[ppm]
7.0
6.8
6.6
6.4

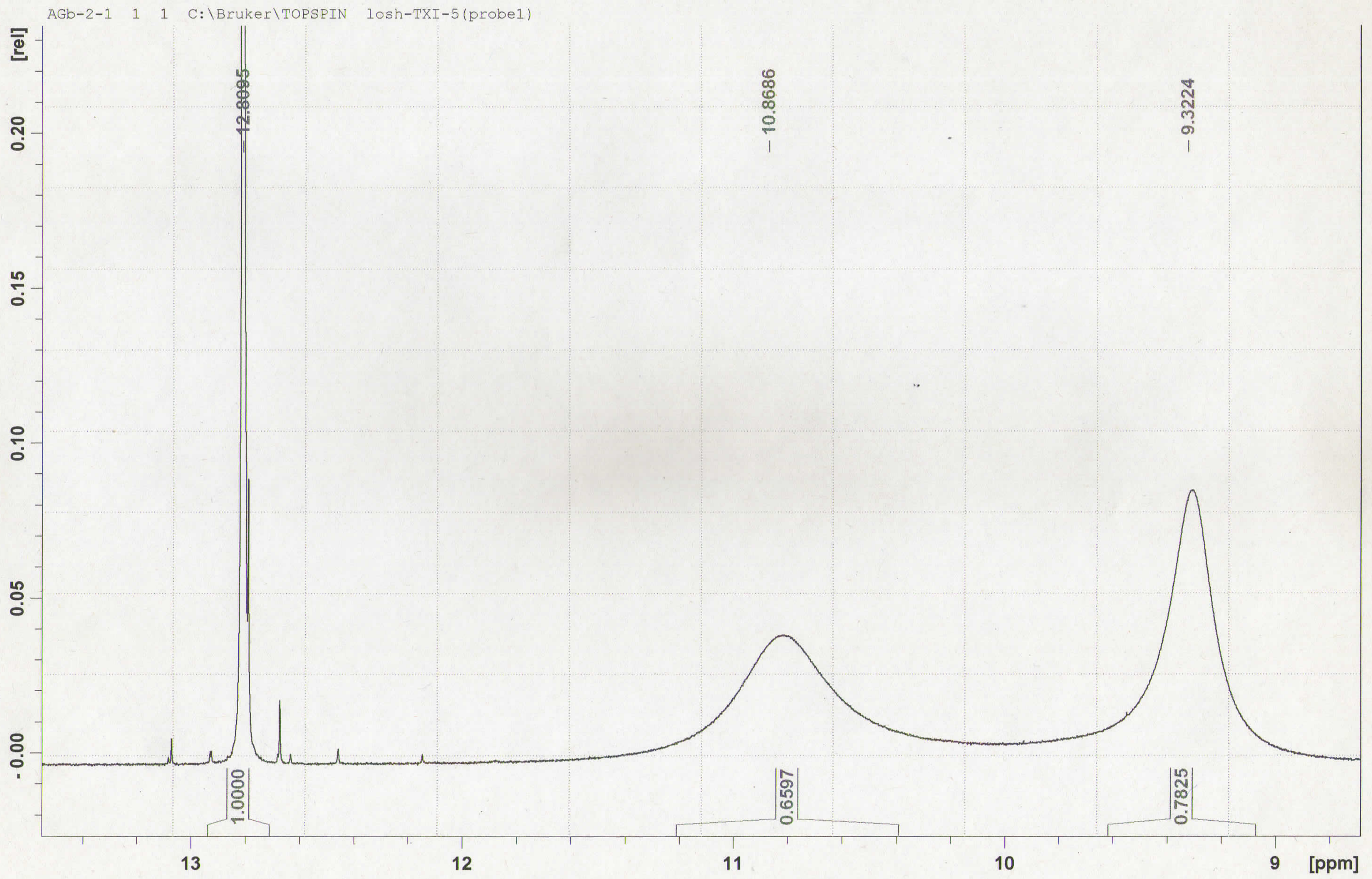

AGb-2-1 1 1 C:\Bruker\TOPSPIN losh-TXI-5(probe1)
[rel]
0.20
0.15
0.10
0.05
- 0.00
12.8095
10.8686
9.3224
1.0000
0.6597
0.7825
13
12
11
10
9
[ppm]

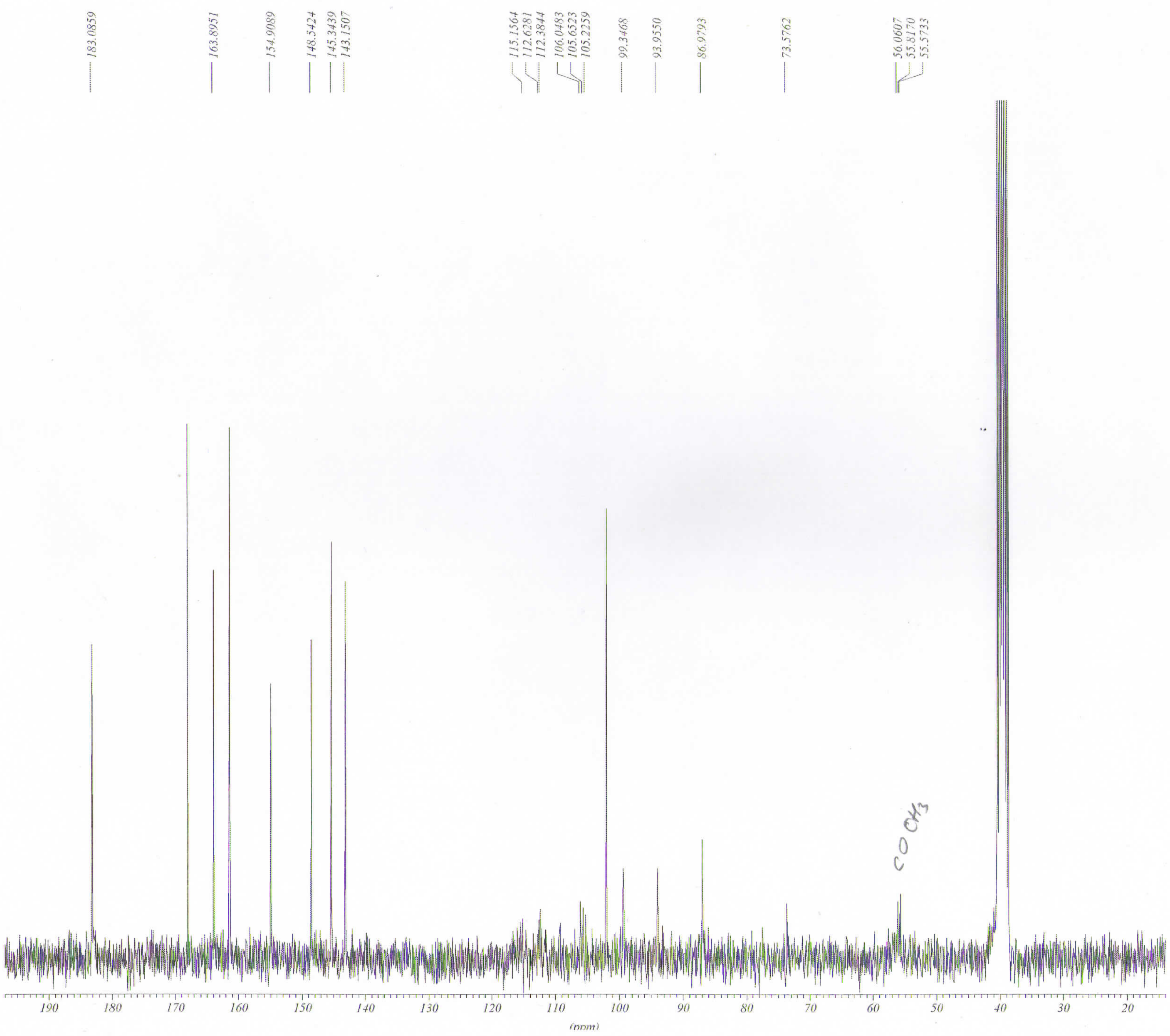

*** Current Data Parameters ***

NAME	:	agb-2-1
EXPNO	:	11
PROCNO	:	1

*** Acquisition Parameters ***

BF1	:	75.4677190 MHz
SOLVENT	:	DMSO

*** Processing Parameters ***

AZFE	:	0.100 ppm

*** 1D NMR Plot Parameters ***

SOLVENT	:	?

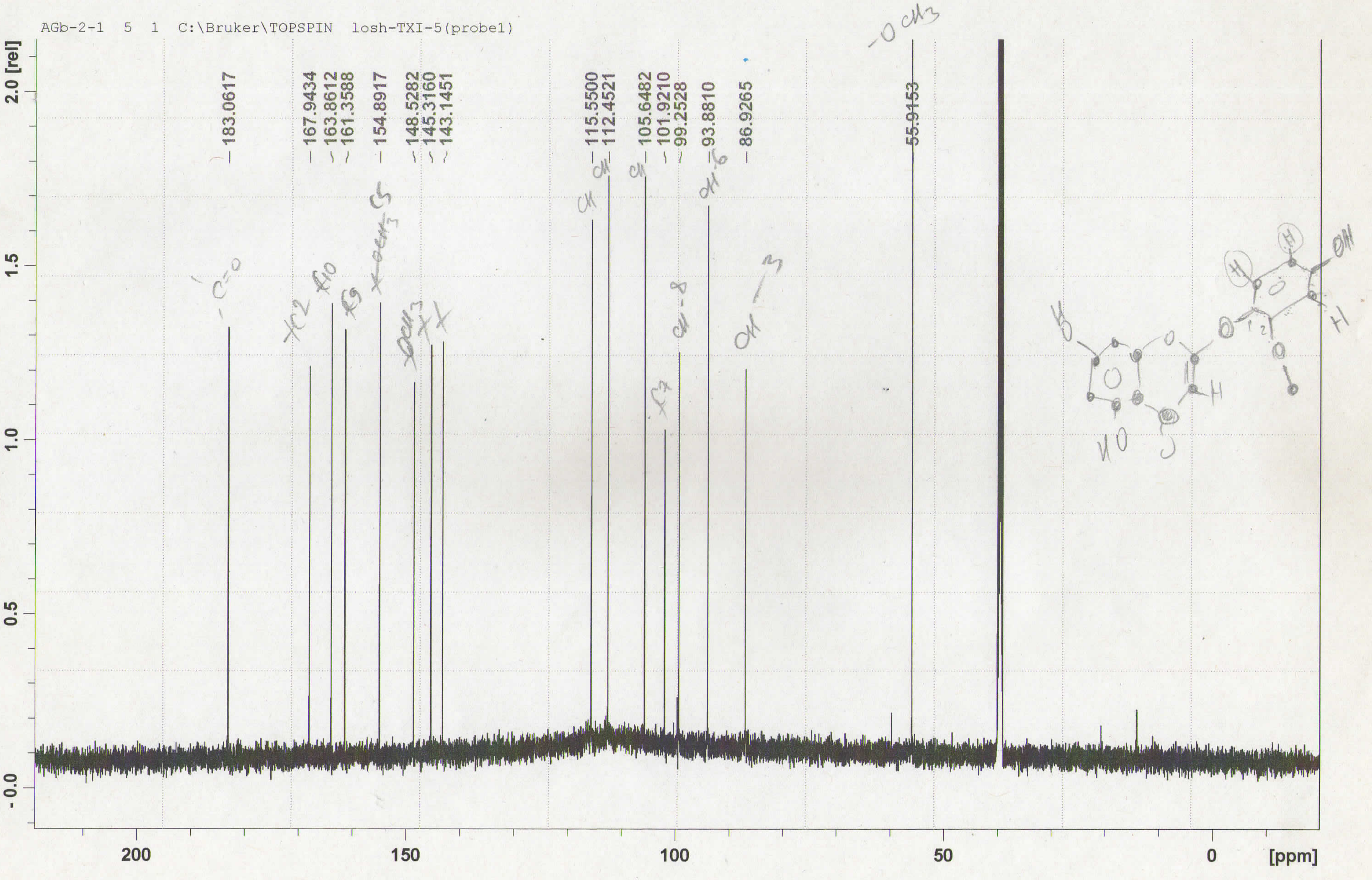
AGb-2-1 5 1 C:\Bruker\TOPSPIN losh-TXI-5(probe1)
183.0617
167.9434
163.8612
161.3588
154.8917
148.5282
145.3160
143.1451
115.5500
112.4521
105.6482
101.9210
99.2528
93.8810
86.9265
55.9153
[rel]
[ppm]

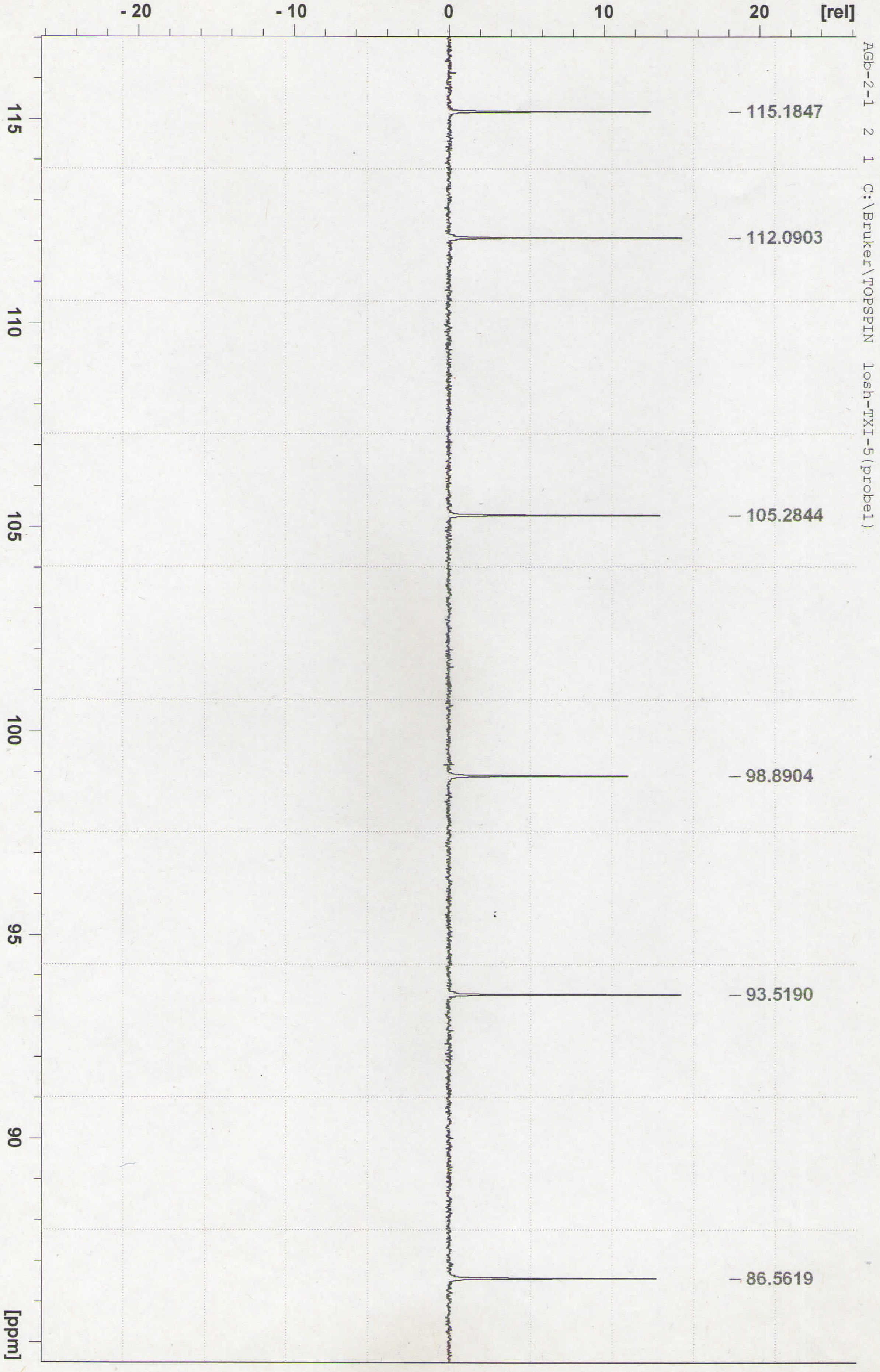
AGb-2-1 2 1 C:\Bruker\TOPSPIN losh-TXI-5(probe1)
- 20
- 10
0
10
20
[rel]
115
110
105
100
95
90
[ppm]
115.1847
112.0903
105.2844
98.8904
93.5190
86.5619

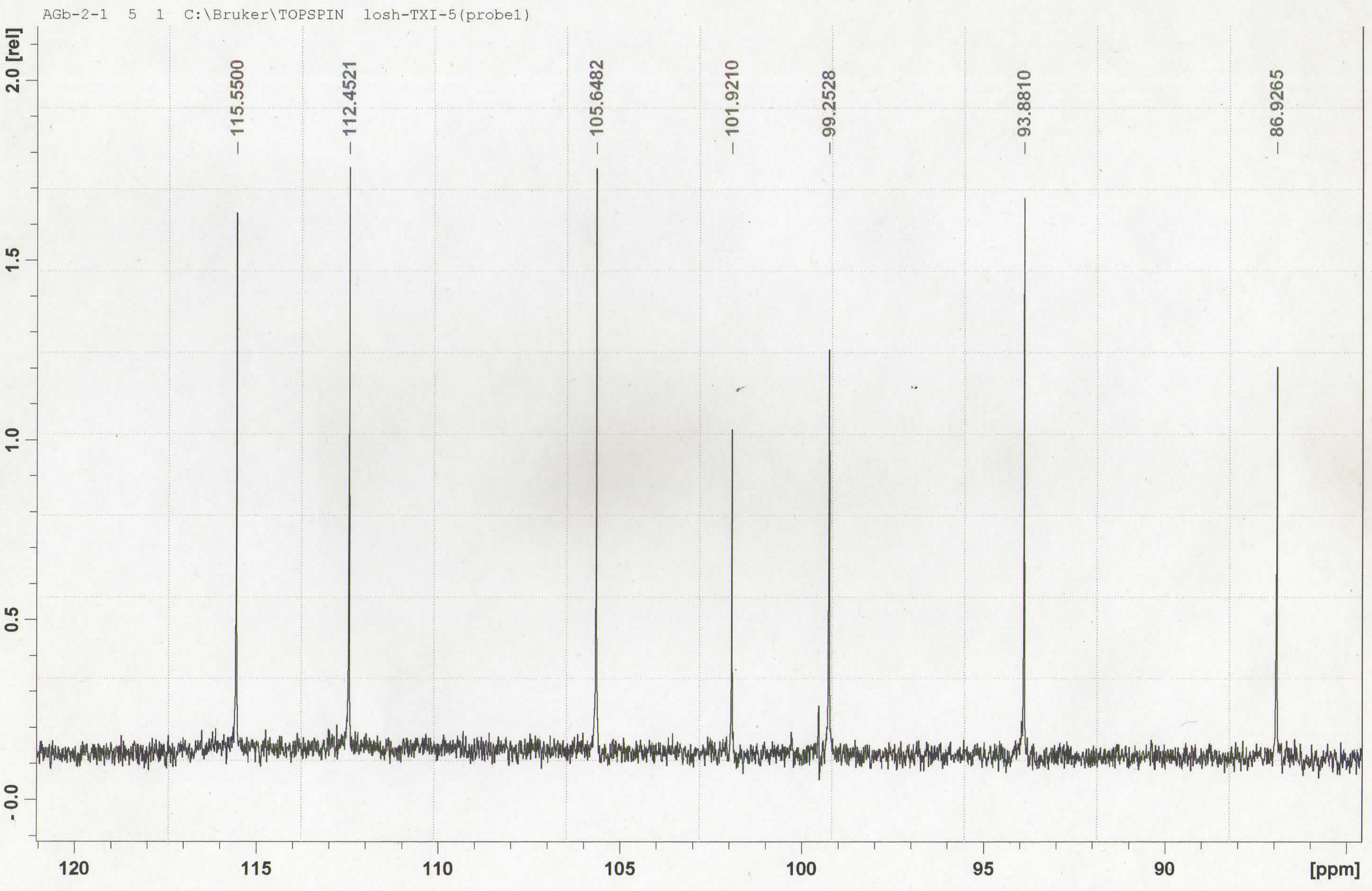
AGb-2-1 5 1 C:\Bruker\TOPSPIN losh-TXI-5(probe1)
115.5500
112.4521
105.6482
101.9210
99.2528
93.8810
86.9265
2.0 [rel]
1.5
1.0
0.5
- 0.0
120
115
110
105
100
95
90
[ppm]

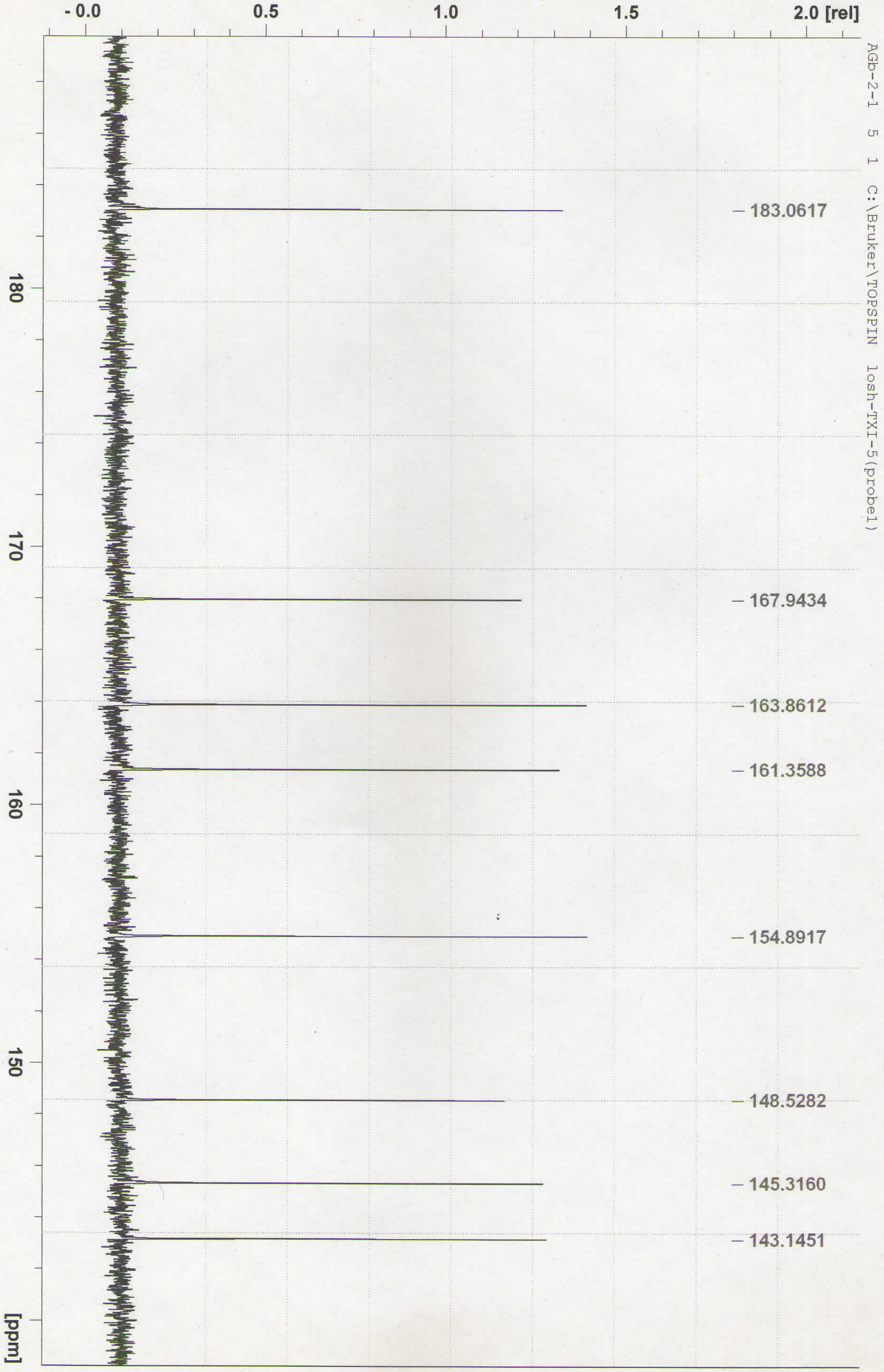

AGb-2-1 5 1 C:\Bruker\TOPSPIN losh-TXI-5(probe1)
- 0.0
0.5
1.0
1.5
2.0 [rel]
183.0617
167.9434
163.8612
161.3588
154.8917
148.5282
145.3160
143.1451
180
170
160
150
[ppm]

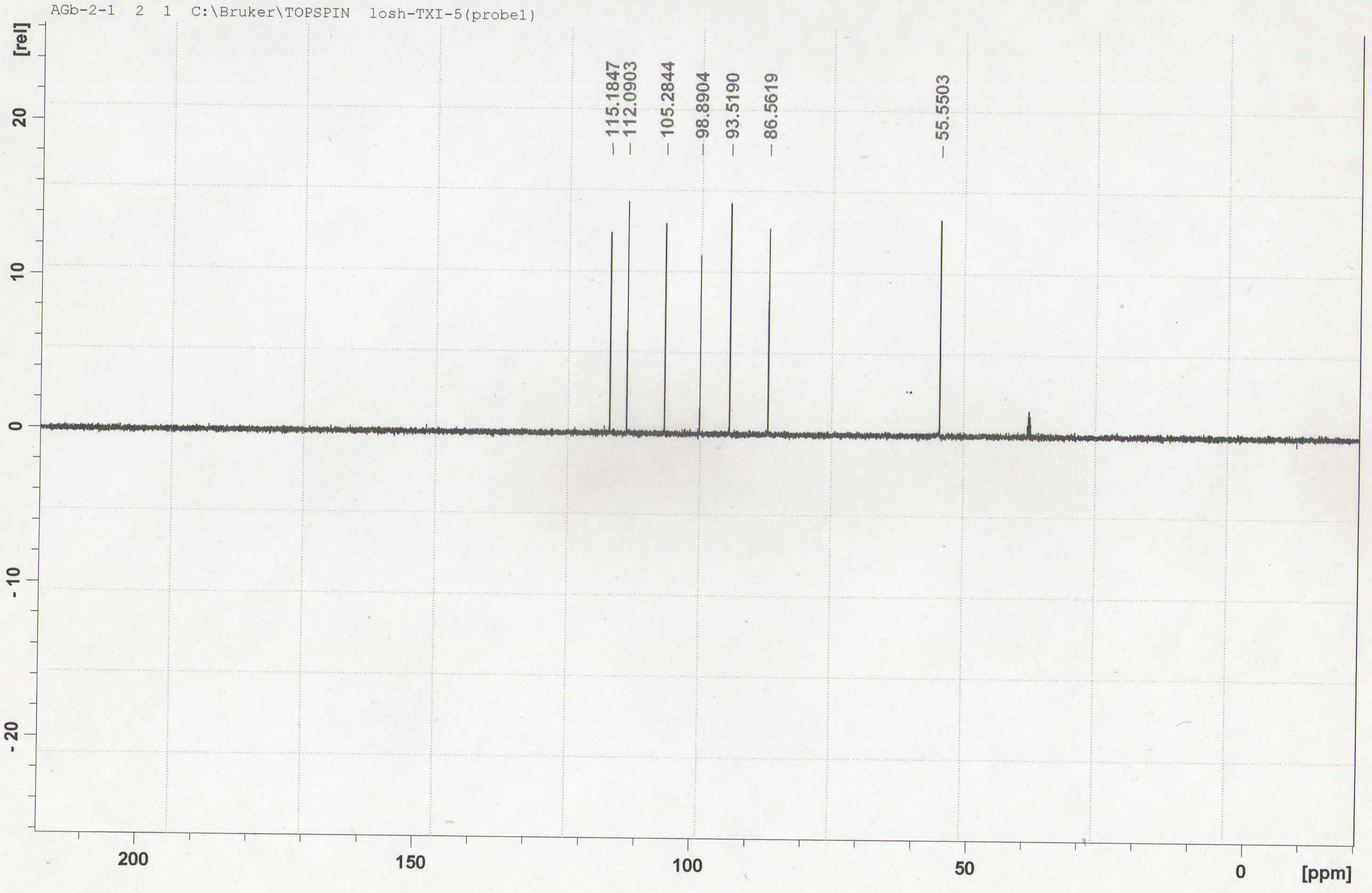

AGb-2-1 2 1 C:\Bruker\TOPSPIN losh-TXI-5(probe1)
[rel]
20
10
0
- 10
- 20
115.1847
112.0903
105.2844
98.8904
93.5190
86.5619
55.5503
200
150
100
50
0
[ppm]

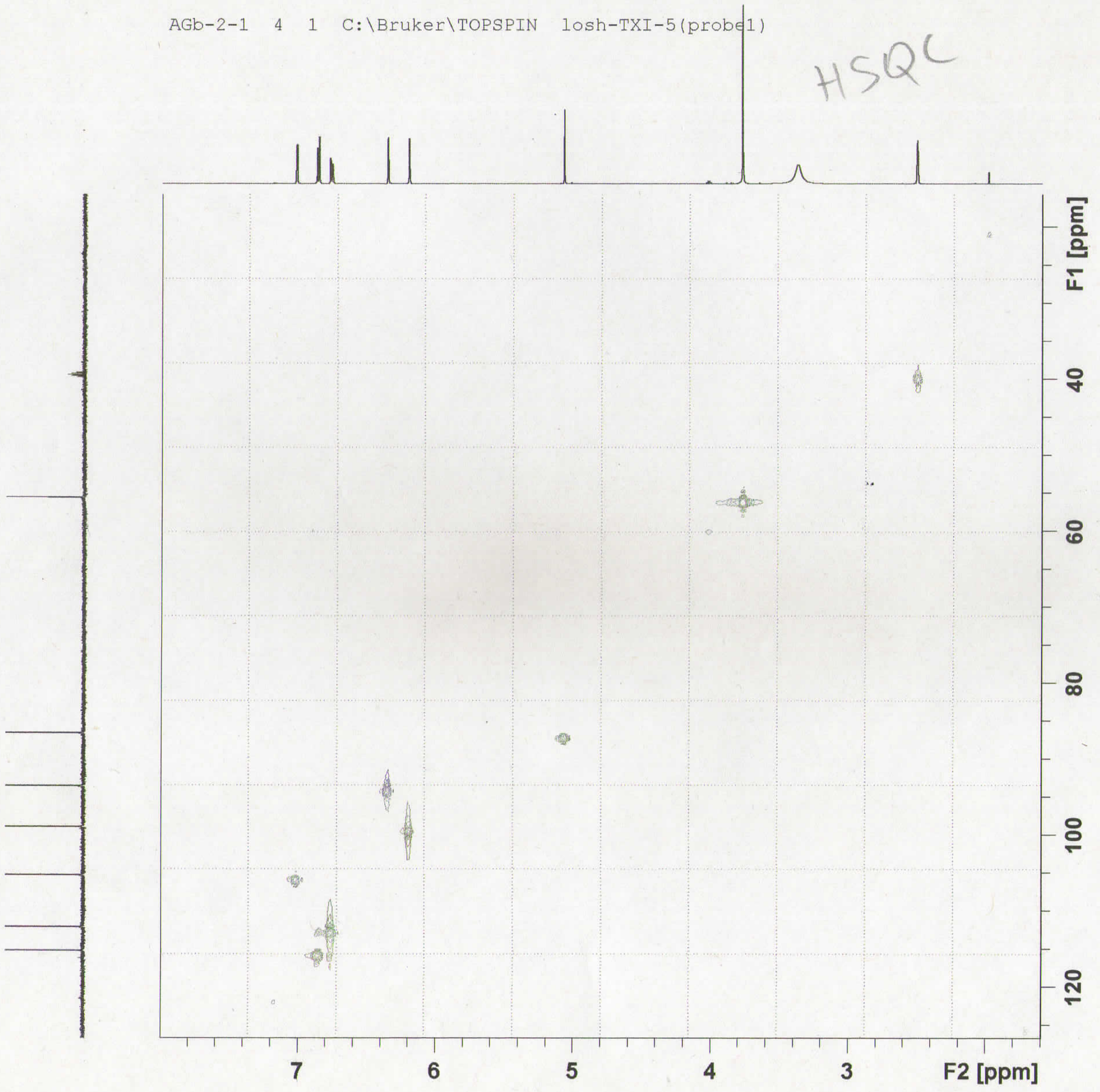

AGb-2-1 4 1 C:\Bruker\TOPSPIN losh-TXI-5(probe1)
HSQC
F1 [ppm]
40
60
80
100
120
7
6
5
4
3
F2 [ppm]

AGb-2-1 4 1 C:\Bruker\TOPSPIN losh-TXI-5(probe1)

HSQC

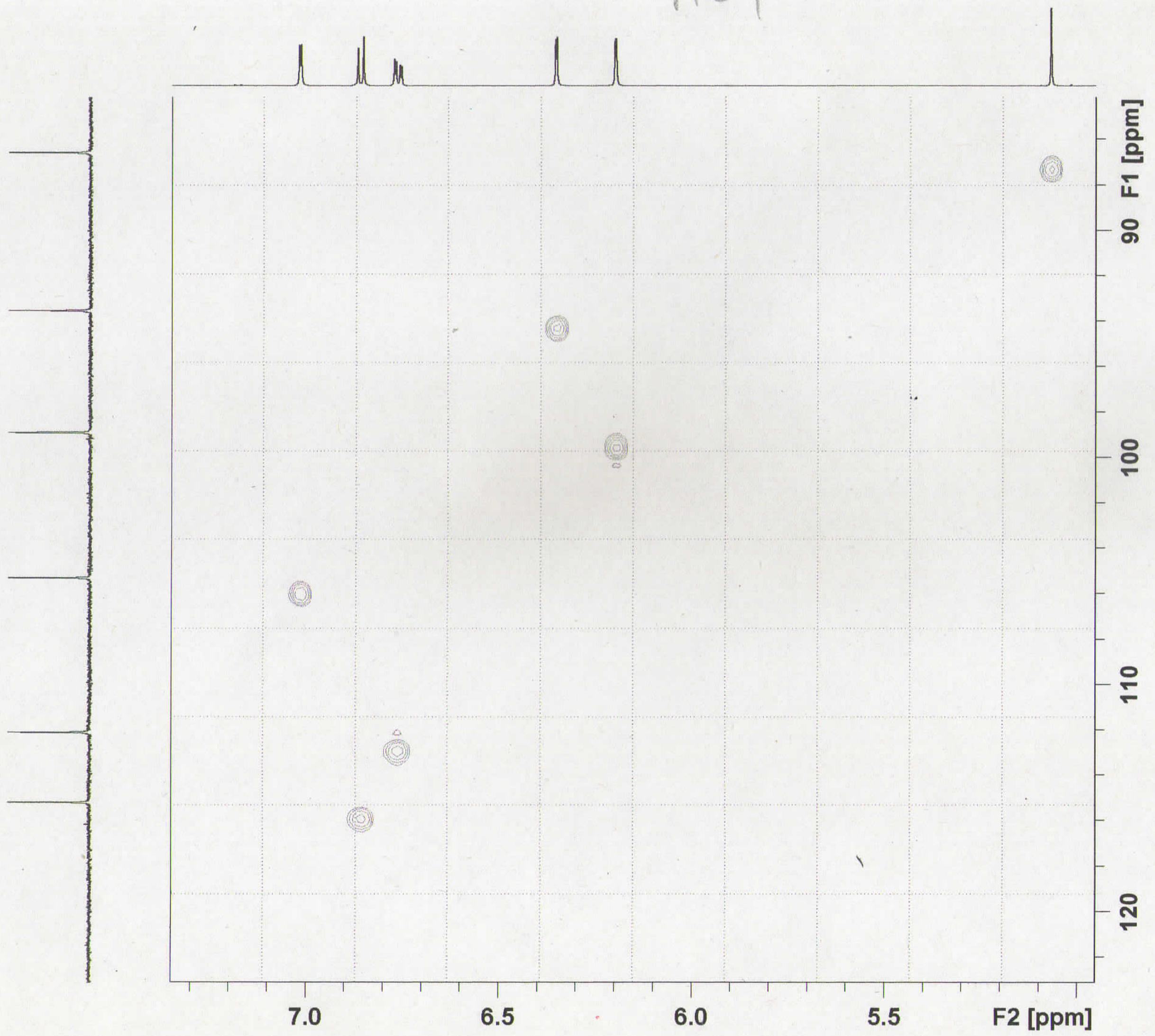

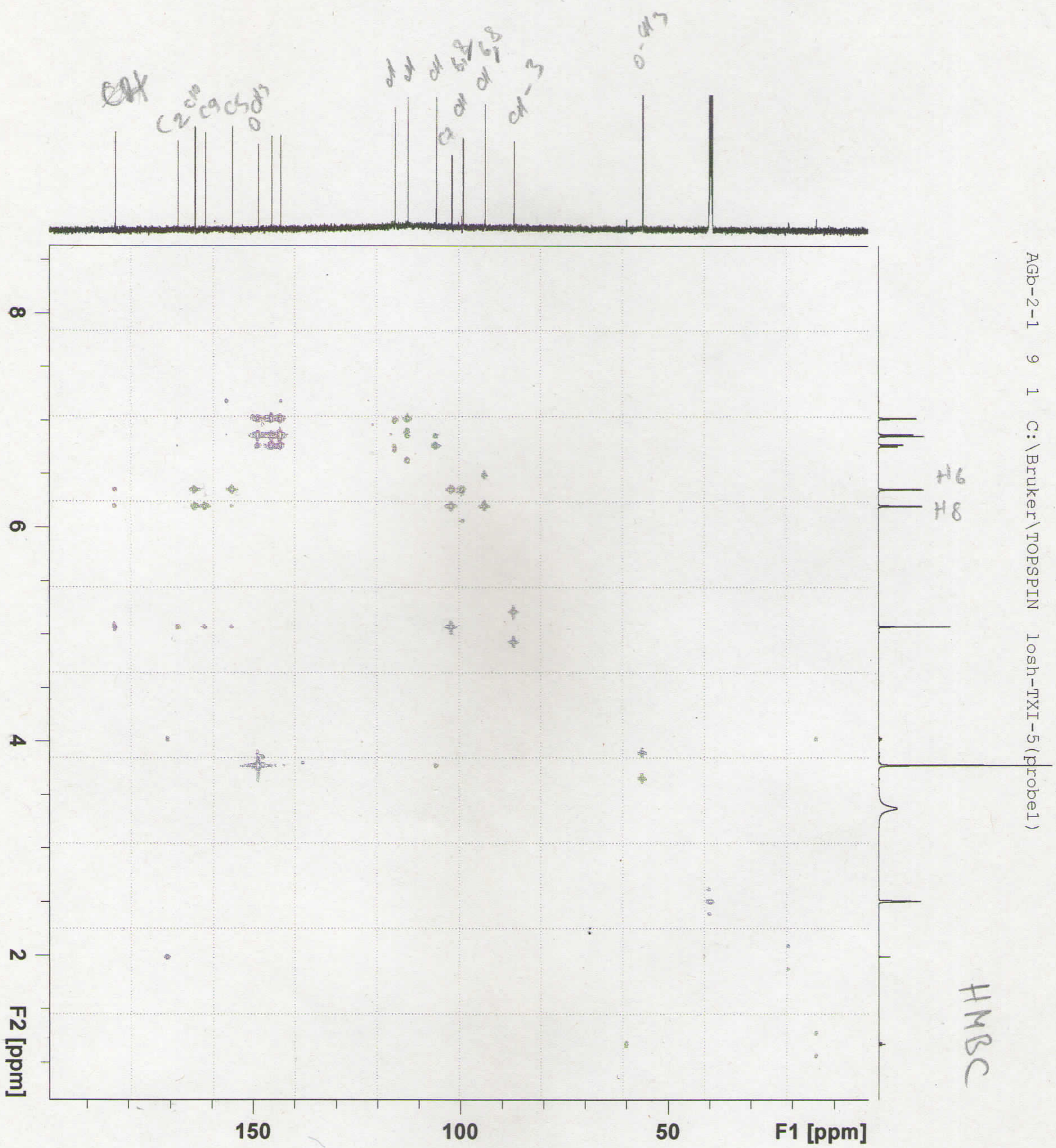

AGb-2-1 9 1 C:\Bruker\TOPSPIN losh-TXI-5(probe1)
8
6
4
2
F2 [ppm]
150
100
50
F1 [ppm]
H6
H8
HMBC

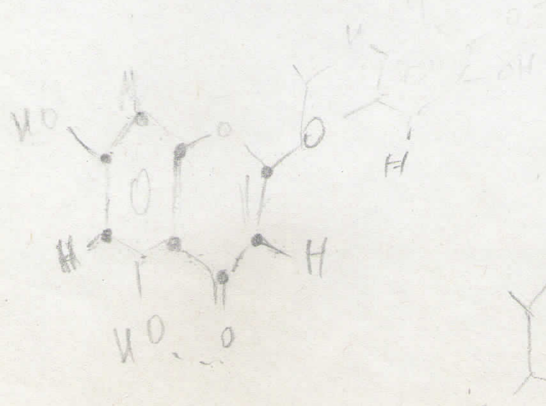

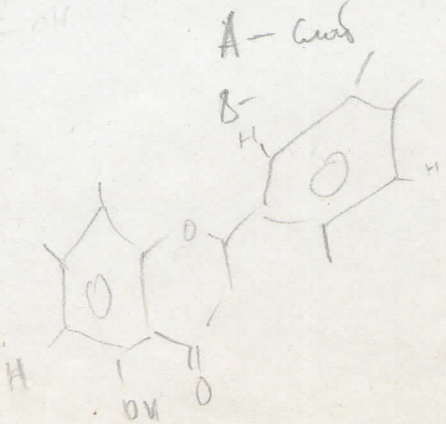

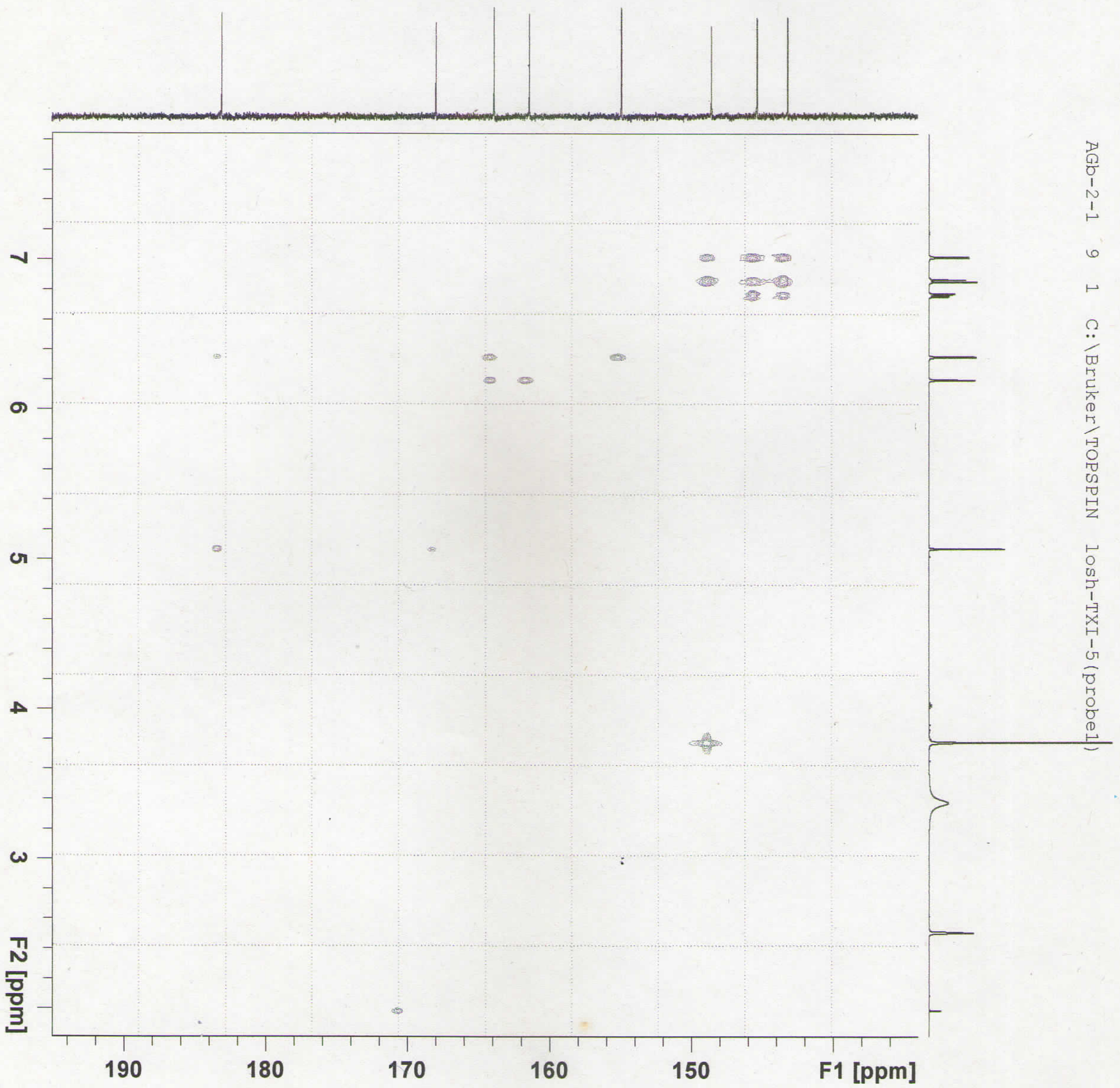
AGb-2-1 9 1 C:\Bruker\TOPSPIN losh-TXI-5(probe1)
F2 [ppm]
7
6
5
4
3
190
180
170
160
150
F1 [ppm]

HMBC

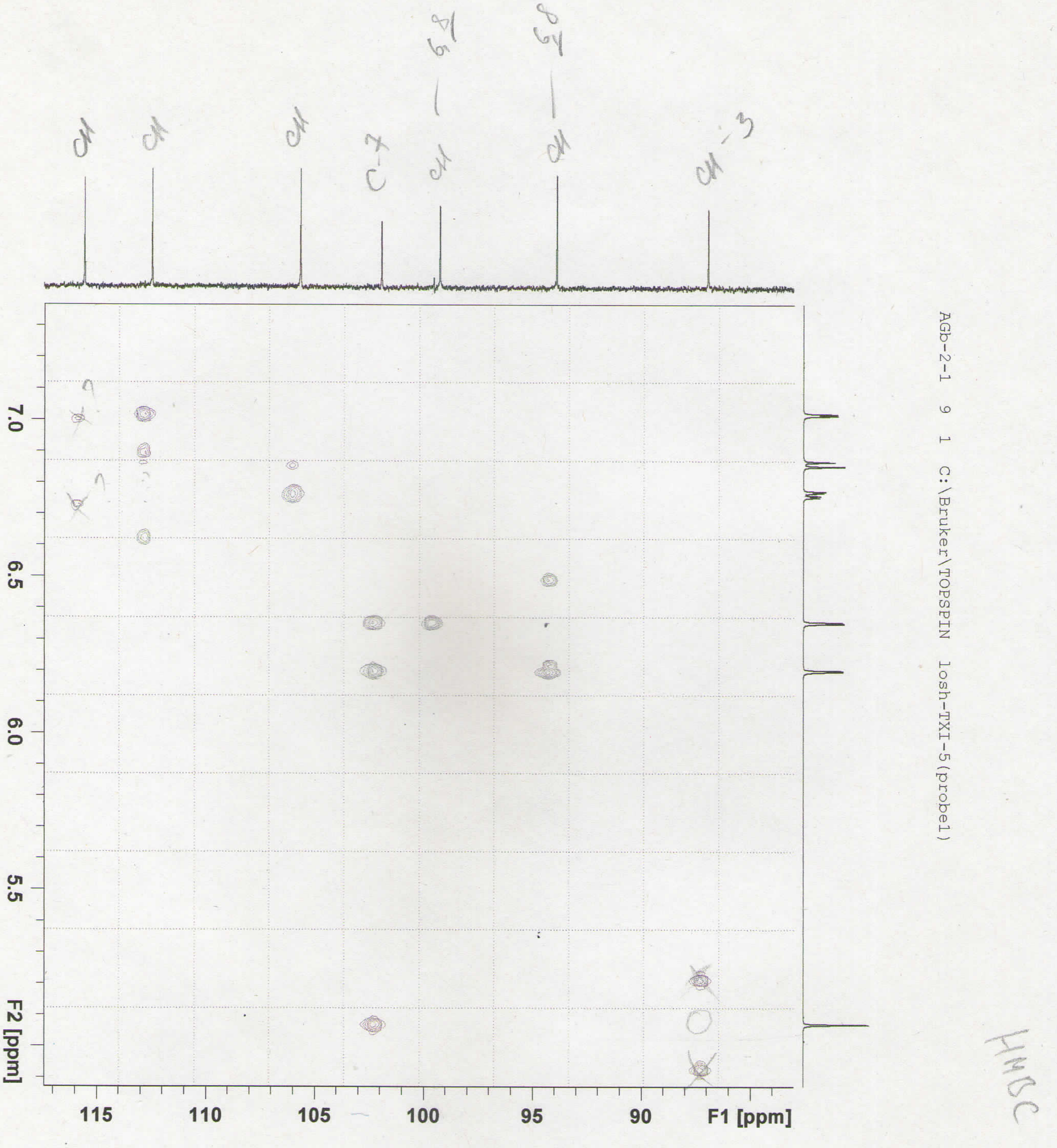
AGb-2-1 9 1 C:\Bruker\TOPSPIN losh-TXI-5(probe1)
HMBC
F2 [ppm]
7.0
6.5
6.0
5.5
F1 [ppm]
115
110
105
100
95
90

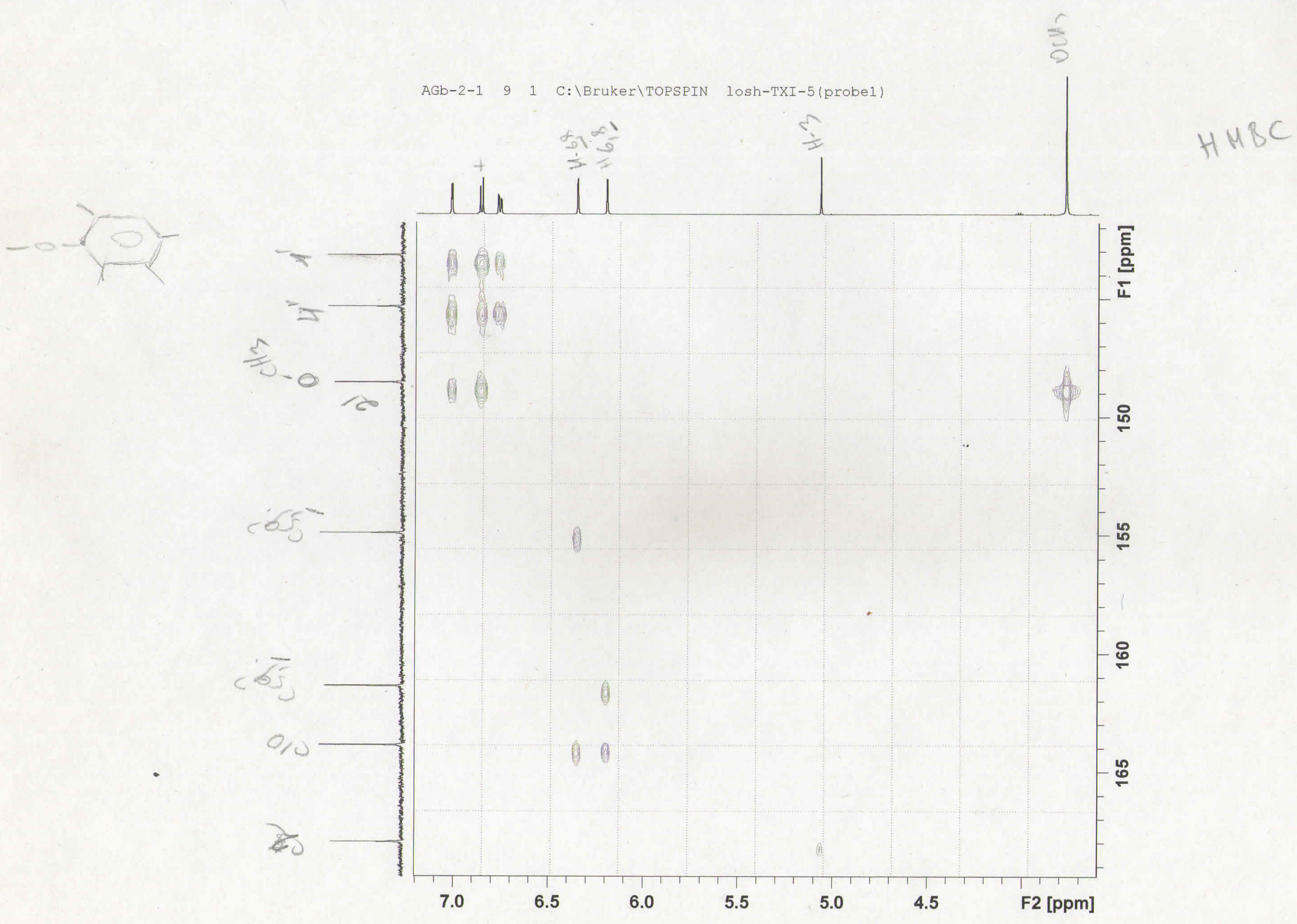
AGb-2-1 9 1 C:\Bruker\TOPSPIN losh-TXI-5(probe1)
HMBC
H-3
OCH3
C10
4'
1'
7.0
6.5
6.0
5.5
5.0
4.5
F2 [ppm]
150
155
160
165
F1 [ppm]

Method

1. General experimental section

NMR spectra are carried out on a commercial instrument (Bruker Avance 300 MHz), chemical shifts are presented in parts per million (ppm) and re-calculated with respect to tetramethylsilane (TMS) (^{1}H) or carbon signals of deuterium solvents (^{13}C). Spin-spin coupling constants (J) are given in hertz (Hz). Refinement of ^{13}C NMR spectra signals is carried out using Dept, HSQC, HMBC NMR spectra. Melting points are determined on a Boetius apparatus. For column chromatography, silica gel 0,06-0,2 mm (Kieselgel) is used as the stationary phase. For TLC, we used ready-made TLC Silica gel 60 F_{254} plates from Merc sprayed with Seebach solution and saturated $KMnO_4$ solution.

2. Plant material

0.94 kg of *Artemisia commutata* and 1.04 kg of *Artemisia glauca* areal parts was extracted with chloroform. Successive chromatographic methods led to the isolation of compounds (**1** and **2**).

To study the component composition of *Artemisia commutata* and *Artemisia glauca*, family *Asteraceae* the aboveground part of plant is collected in the Eastern-Kazakhstan region (Western Altai Mountains) on, phase of blooming – beginning of flowering.

Species is identified by botanists of the Altai Botanical Garden (Rider city, eastern Kazakhstan).

3. Extraction and isolation

0.94 kg of *Artemisia commutata* and 1.04 kg of *Artemisia glauca* areal parts was extracted with chloroform. This operation is repeated three times. The solvent is evaporated on a rotary evaporator under the vacuum of a water-jet pump to obtain $CHCl_3$ extracts, which is used for preparative chromatographic separation by column chromatography on silica gel.

4. Molecular Similarity

Molecular Similarity of commutin against the eight co-crystallized ligand of SARS-Cov-2 was carried out calculated using Discovery studio 4.0. At first, the CHARMM force field was applied then the compounds were prepared using prepare ligand protocol. Then, the tested compounds were used as a test set while the co-crystallized ligand was used as a reference compounds. The protocol was adjusted to give one output. The default molecular properties were applied. The molecular properties include number of rotatable bonds, number of rings, number of aromatic rings, number of hydrogen bond donors (HBA), number of hydrogen bond acceptors (HBD), partition coefficient (ALog p), molecular weight (M. Wt), and molecular fractional polar surface area (MFPSA).

5. Fingerprint study

Fingerprint study of commutin against the eight co-crystallized ligand of SARS-Cov-2 was carried out calculated using Discovery studio 4.0. At first, the CHARMM force field was applied then the compounds were prepared using prepare ligand protocol. Then, the tested compounds were used as a test set while the co-crystallized ligand was used as a reference compounds. The protocol was adjusted to give the most related co-crystallized ligand to the tested compounds. The default molecular properties were applied. The used fingerprints were based on some parameters related to type of atoms which may be one of the following: charge, hybridization, H-bond acceptor, H-bond donor, Positive ionizable, Negative ionizable, Halogen, Aromatic, or None of the above. In addition, it includes the ALogP category of atoms.

6. DFT

The DFT parameters (total energy, binding energy, HOMO, LUMO, gap energy, dipole moment, and electrostatic potential) were calculated using Discovery studio software. the tested compounds were prepared using prepare ligand protocol. Then, the prepared compounds were subjected to DFT calculation protocol using the default option

7. Docking studies

Crystal structure of SARS-Cov-2 Papain Like Protease, PLP, (PDB ID: 3E9S) was obtained from Protein Data Bank. The docking investigation was accomplished using MOE2014 software. At first, the crystal structure of PLP, (PDB ID: 3E9S) was prepared by removing water molecules. Only one chain was retained beside the co-crystallized ligand **TTT**, (5-amino-2-methyl-*N*-[(1R)-1-naphthalen-1-ylethyl]benzamide). Then, the selected chain was protonated and subjected to minimization of energy process. Next, the active site of the target protein was defined.

Structures of the tested compounds and the co-crystallized ligand were drawn using ChemBioDraw Ultra 14.0 and saved as MDL-SD format. Such file was opened using MOE to display the 3D structures which were protonated and subjected to energy minimization. Formerly, validation of the docking process was performed by docking the co-crystallized ligand against the isolated pocket of active site. The produced RMSD value indicated the validity of process. Finally, docking of the tested compounds was done through the dock option inserted in compute window. For each docked molecule, 30 docked poses were produced using ASE for scoring function and force field for refinement. The results of the docking process were then visualized using Discovery Studio 4.0 software.

8. ADMET

ADMET descriptors (absorption, distribution, metabolism, excretion and toxicity) of the compounds were determined using Discovery studio 4.0. At first, the CHARMM force field was applied then the tested compounds were prepared and minimized according to the preparation of small molecule protocol. Then ADMET descriptors protocol was applied to carry out these studies.

9. Toxicity studies

The toxicity parameters of the tested compounds were calculated using Discovery studio 4.0. Indinavir was used as a reference drug. At first, the CHARMM force field was applied then

the compounds were prepared and minimized according to the preparation of small molecule protocol. Then different parameters were calculated from the toxicity prediction (extensible) protocol.

10. Molecular dynamics simulations

The system was prepared using the web-based CHARMM-GUI[1-3] interface with the CHARMM36 force field[4]. All the simulations were done using the NAMD 2.13[5] package. The TIP3P explicit solvation model was used[6], and the periodic boundary conditions were set with a dimension of the dimensions ---- Å, -----Å, and ----- Å in x, y, and z, respectively. The parameters for the top docking results were generated using the CHARMM general force field[7] Afterward, the system was neutralized using ---- (Cl^-/Na^+) ions. The MD protocols involved minimization, equilibration, and production.a 2 *fs* time step of integration was chosen for all MD simulations, the equilibration was carried in the canonical (*NVT*) ensemble, while the isothermal–isobaric (*NPT*) ensemble was for the production. Through the 100 *ns* of MD production, the pressure was set at 1 atm using the Nose´–Hoover Langevin piston barostat[8,9] with a Langevin piston decay of 0.05*ps* and a period of 0.1*ps*. The temperature was set at 298.15 K using the Langevin thermostat[10]. A distance cutoff of 12.0 Å was applied to short-range nonbonded interactions with a pair list distance of 16 Å, and Lennard Jones interactions were smoothly truncated at 8.0 Å. Long-range electrostatic interactions were treated using the particle-mesh Ewald (PME) method[11,12], where a grid spacing of 1.0 Å was used for all simulation cells. All covalent bonds involving hydrogen atoms were constrained using the SHAKE algorithm[13]. For consistency, we have applied the same protocol for all MD simulations.

Binding Energy Calculations

The one-average molecular mechanics generalized Born surface area (MM/GBSA)[14,15]approach implemented in the MOLAICAL code[16] was used for the relative binding energy calculations, in which the ligand (L) binds to the protein receptor (R) to form the complex (RL),

$$\Delta G_{bind} = \Delta G_{RL} - \Delta G_R - \Delta G_L$$

which can be represented by contributions of different interactions,

$$\Delta G_{bind} = \Delta H - T\Delta S = \Delta E_{MM} + \Delta G_{Sol} - T\Delta S$$

where the changes in the gas phase molecular mechanics (ΔE_{MM}), solvation Gibbs energy (ΔG_{Sol}), and conformational entropy $(-T\Delta S)$ are determined as follows: ΔE_{MM} is the sum of the changes in the electrostatic energies ΔE_{ele}, the van der Waals energies ΔE_{vdW}, and the internal energies ΔE_{int} (bonded interactions); ΔG_{Sol} is the total of both the polar solvation (calculated using the generalized Born model) and the nonpolar solvation (calculated using the solvent-accessible surface area) and $-T\Delta S$ is calculated by the normal mode analysis. The solvent dielectric constant of 78.5 and the surface tension constant of 0.03012 kJ mol^{-1} $Å^2$ were used for MM/GBSA calculations.

1. Jo, S.; Kim, T.; Iyer, V.G.; Im, W. CHARMM-GUI: A web-based graphical user interface for CHARMM. *Journal of computational chemistry* **2008**, *29*, 1859-1865, doi:https://doi.org/10.1002/jcc.20945.
2. Brooks, B.R.; Brooks III, C.L.; Mackerell Jr., A.D.; Nilsson, L.; Petrella, R.J.; Roux, B.; Won, Y.; Archontis, G.; Bartels, C.; Boresch, S., et al. CHARMM: The biomolecular simulation program. *Journal of computational chemistry* **2009**, *30*, 1545-1614, doi:https://doi.org/10.1002/jcc.21287.
3. Lee, J.; Cheng, X.; Swails, J.M.; Yeom, M.S.; Eastman, P.K.; Lemkul, J.A.; Wei, S.; Buckner, J.; Jeong, J.C.; Qi, Y., et al. CHARMM-GUI Input Generator for NAMD, GROMACS, AMBER, OpenMM, and CHARMM/OpenMM Simulations Using the CHARMM36 Additive Force Field. *Journal of chemical theory and computation* **2016**, *12*, 405-413, doi:10.1021/acs.jctc.5b00935.
4. Best, R.B.; Zhu, X.; Shim, J.; Lopes, P.E.; Mittal, J.; Feig, M.; Mackerell, A.D., Jr. Optimization of the additive CHARMM all-atom protein force field targeting improved sampling of the backbone phi, psi and side-chain chi(1) and chi(2) dihedral angles. *Journal of chemical theory and computation* **2012**, *8*, 3257-3273, doi:10.1021/ct300400x.
5. Phillips, J.C.; Braun, R.; Wang, W.; Gumbart, J.; Tajkhorshid, E.; Villa, E.; Chipot, C.; Skeel, R.D.; Kale, L.; Schulten, K. Scalable molecular dynamics with NAMD. *Journal of computational chemistry* **2005**, *26*, 1781-1802, doi:10.1002/jcc.20289.
6. Jorgensen, W.L.; Chandrasekhar, J.; Madura, J.D.; Impey, R.W.; Klein, M.L. Comparison of simple potential functions for simulating liquid water. *The Journal of Chemical Physics* **1983**, *79*, 926-935, doi:10.1063/1.445869.
7. Yu, W.; He, X.; Vanommeslaeghe, K.; MacKerell, A.D., Jr. Extension of the CHARMM General Force Field to sulfonyl-containing compounds and its utility in biomolecular simulations. *Journal of computational chemistry* **2012**, *33*, 2451-2468, doi:10.1002/jcc.23067.
8. Nosé, S.; Klein, M.L. Constant pressure molecular dynamics for molecular systems. *Molecular Physics* **1983**, *50*, 1055-1076, doi:10.1080/00268978300102851.
9. Nosé, S. A molecular dynamics method for simulations in the canonical ensemble. *Molecular Physics* **1984**, *52*, 255-268, doi:10.1080/00268978400101201.
10. Grest, G.S.; Kremer, K. Molecular dynamics simulation for polymers in the presence of a heat bath. *Physical review. A, General physics* **1986**, *33*, 3628-3631, doi:10.1103/physreva.33.3628.
11. Darden, T.; York, D.; Pedersen, L. Particle mesh Ewald: AnN·log(N) method for Ewald sums in large systems. *The Journal of Chemical Physics* **1993**, *98*, 10089-10092, doi:10.1063/1.464397.
12. Essmann, U.; Perera, L.; Berkowitz, M.L.; Darden, T.; Lee, H.; Pedersen, L.G. A smooth particle mesh Ewald method. *The Journal of Chemical Physics* **1995**, *103*, 8577-8593, doi:10.1063/1.470117.

13. Ryckaert, J.-P.; Ciccotti, G.; Berendsen, H.J.C. Numerical integration of the cartesian equations of motion of a system with constraints: molecular dynamics of n-alkanes. *Journal of Computational Physics* **1977**, *23*, 327-341, doi:https://doi.org/10.1016/0021-9991(77)90098-5.
14. Genheden, S.; Ryde, U. Comparison of end-point continuum-solvation methods for the calculation of protein-ligand binding free energies. *Proteins* **2012**, *80*, 1326-1342, doi:10.1002/prot.24029.
15. Wang, E.; Sun, H.; Wang, J.; Wang, Z.; Liu, H.; Zhang, J.Z.H.; Hou, T. End-Point Binding Free Energy Calculation with MM/PBSA and MM/GBSA: Strategies and Applications in Drug Design. *Chemical reviews* **2019**, *119*, 9478-9508, doi:10.1021/acs.chemrev.9b00055.
16. Bai, Q.; Tan, S.; Xu, T.; Liu, H.; Huang, J.; Yao, X. MolAICal: a soft tool for 3D drug design of protein targets by artificial intelligence and classical algorithm. *Briefings in bioinformatics* **2021**, *22*, bbaa161.

Toxicity Report

Flavonoid-1

TOPKAT_Developmental_Toxicity_Potential

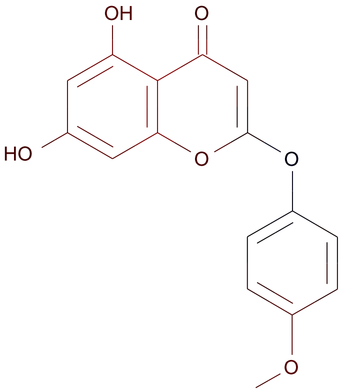

$C_{16}H_{12}O_6$
Molecular Weight: 300.26287
ALogP: 3.129
Rotatable Bonds: 3
Acceptors: 6
Donors: 2

Model Prediction

Prediction: Toxic
Probability: 0.69
Enrichment: 1.31
Bayesian Score: 3.65
Mahalanobis Distance: 10.8
Mahalanobis Distance p-value: 0.00203

Prediction: Positive if the Bayesian score is above the estimated best cutoff value from minimizing the false positive and false negative rate.
Probability: The esimated probability that the sample is in the positive category. This assumes that the Bayesian score follows a normal distribution and is different from the prediction using a cutoff.
Enrichment: An estimate of enrichment, that is, the increased likelihood (versus random) of this sample being in the category.
Bayesian Score: The standard Laplacian-modified Bayesian score.
Mahalanobis Distance: The Mahalanobis distance (MD) is the distance to the center of the training data. The larger the MD, the less trustworthy the prediction.
Mahalanobis Distance p-value: The p-value gives the fraction of training data with an MD greater than or equal to the one for the given sample, assuming normally distributed data. The smaller the p-value, the less trustworthy the prediciton. For highly non-normal X properties (e.g., fingerprints), the MD p-value is wildly inaccurate.

Structural Similar Compounds

Name	D&C Yellow 8	Benomyl	Sulfonylurea Gliclazide
Structure	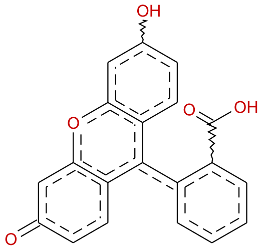	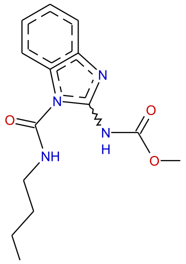	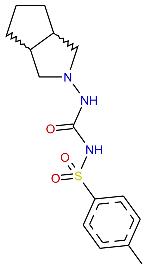
Actual Endpoint	Non-Toxic	Toxic	Toxic
Predicted Endpoint	Non-Toxic	Toxic	Toxic
Distance	0.506	0.635	0.643
Reference	Food Chem Toxicol 24:819-823; 1986	J Toxicol Environ Health 17:405-417; 1986	Yakuri to Chiryo 9:3551-3571; 1981

Model Applicability

Unknown features are fingerprint features in the query molecule, but not found or appearing too infrequently in the training set.

1. All properties and OPS components are within expected ranges.

Feature Contribution

Top features for positive contribution

Fingerprint	Bit/Smiles	Feature Structure	Score	Toxic in training set
SCFP_6	1237755852	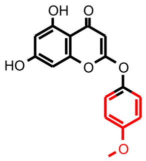 CO[c]1:[cH]:[cH]:[*]:[cH]:[cH]:1	0.453	8 out of 9

SCFP_6	591469355	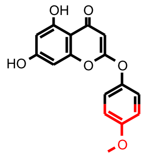 [*]:[cH]:[c](OC):[cH]:[*]	0.411	10 out of 12
SCFP_6	1534870744	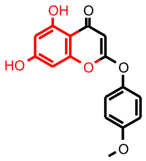 [*]O[c]1:[cH]:[c](O):[cH]:[c](O):[c]:1[*]	0.381	2 out of 2
**Top Features for negative contribution**				
**Fingerprint**	**Bit/Smiles**	**Feature Structure**	**Score**	**Toxic in training set**
SCFP_6	-609499983	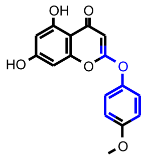 [*]C(=[*])O[c]1:[cH]:[cH]:[*]:[cH]:[cH]:1	-0.422	0 out of 1
SCFP_6	3	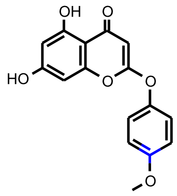 [*][c](:[*]):[*]	0	92 out of 181
SCFP_6	2019093677	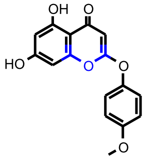 [*]C(=[*])O[c](:[*]):[*]	0	4 out of 8

Flavonoid-2

TOPKAT_Developmental_Toxicity_Potential

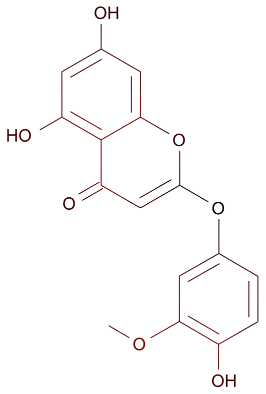

$C_{16}H_{12}O_7$
Molecular Weight: 316.26227
ALogP: 2.887
Rotatable Bonds: 3
Acceptors: 7
Donors: 3

Model Prediction

Prediction: Toxic
Probability: 0.721
Enrichment: 1.37
Bayesian Score: 4.47
Mahalanobis Distance: 11.2
Mahalanobis Distance p-value: 0.000561

Prediction: Positive if the Bayesian score is above the estimated best cutoff value from minimizing the false positive and false negative rate.
Probability: The esimated probability that the sample is in the positive category. This assumes that the Bayesian score follows a normal distribution and is different from the prediction using a cutoff.
Enrichment: An estimate of enrichment, that is, the increased likelihood (versus random) of this sample being in the category.
Bayesian Score: The standard Laplacian-modified Bayesian score.
Mahalanobis Distance: The Mahalanobis distance (MD) is the distance to the center of the training data. The larger the MD, the less trustworthy the prediction.
Mahalanobis Distance p-value: The p-value gives the fraction of training data with an MD greater than or equal to the one for the given sample, assuming normally distributed data. The smaller the p-value, the less trustworthy the prediciton. For highly non-normal X properties (e.g., fingerprints), the MD p-value is wildly inaccurate.

Structural Similar Compounds

Name	D&C Yellow 8	Ochratoxin a	Quercetin
Structure	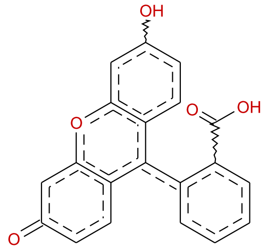	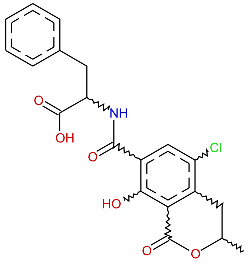	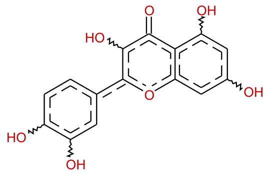
Actual Endpoint	Non-Toxic	Toxic	Toxic
Predicted Endpoint	Non-Toxic	Toxic	Toxic
Distance	0.630	0.647	0.679
Reference	Food Chem Toxicol 24:819-823; 1986	Toxicol Appl Pharmacol 37(2):331-8; 1976	Food Chem Toxicol 20(1):75-9; 1982

Model Applicability

Unknown features are fingerprint features in the query molecule, but not found or appearing too infrequently in the training set.

1. All properties and OPS components are within expected ranges.

Feature Contribution

Top features for positive contribution

Fingerprint	Bit/Smiles	Feature Structure	Score	Toxic in training set
SCFP_6	2116304939	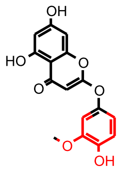 [*]O[c]1:[cH]:[*]:[cH]:[cH]:[c]:1O	0.504	5 out of 5

SCFP_6	591469355	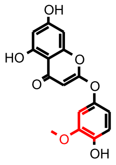 [*]:[cH]:[c](OC):[cH]:[*]	0.411	10 out of 12
SCFP_6	-617610981	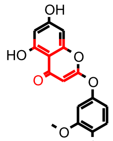 [*][c](:[*]):[c]1C(=O)C=C([*])[*][c]:1:[*]	0.381	2 out of 2
**Top Features for negative contribution**				
**Fingerprint**	**Bit/Smiles**	**Feature Structure**	**Score**	**Toxic in training set**
SCFP_6	-609499983	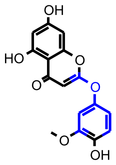 [*]C(=[*])O[c]1:[cH]:[cH]:[*]:[cH]:[cH]:1	-0.422	0 out of 1
SCFP_6	12	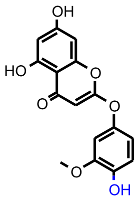 [*]O[*]	0	97 out of 178
SCFP_6	1	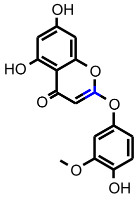 [*]C(=[*])[*]	0	90 out of 173

remdesivir

TOPKAT_Developmental_Toxicity_Potential

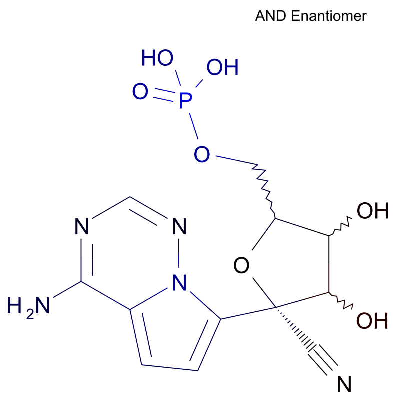

$C_{12}H_{14}N_5O_7P$
Molecular Weight: 371.24262
ALogP: -1.502
Rotatable Bonds: 4
Acceptors: 11
Donors: 5

Model Prediction

Prediction: Non-Toxic
Probability: 0.373
Enrichment: 0.709
Bayesian Score: -5.42
Mahalanobis Distance: 9.05
Mahalanobis Distance p-value: 0.163

Prediction: Positive if the Bayesian score is above the estimated best cutoff value from minimizing the false positive and false negative rate.
Probability: The esimated probability that the sample is in the positive category. This assumes that the Bayesian score follows a normal distribution and is different from the prediction using a cutoff.
Enrichment: An estimate of enrichment, that is, the increased likelihood (versus random) of this sample being in the category.
Bayesian Score: The standard Laplacian-modified Bayesian score.
Mahalanobis Distance: The Mahalanobis distance (MD) is the distance to the center of the training data. The larger the MD, the less trustworthy the prediction.
Mahalanobis Distance p-value: The p-value gives the fraction of training data with an MD greater than or equal to the one for the given sample, assuming normally distributed data. The smaller the p-value, the less trustworthy the prediciton. For highly non-normal X properties (e.g., fingerprints), the MD p-value is wildly inaccurate.

Structural Similar Compounds

Name	Sinigrin (Free Acid Form)	Azthreonam	Vidarabine
Structure	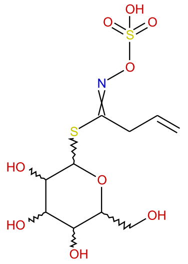	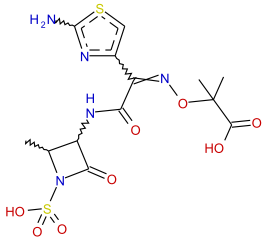	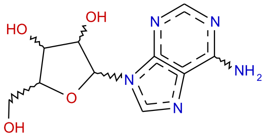
Actual Endpoint	Toxic	Non-Toxic	Non-Toxic
Predicted Endpoint	Toxic	Non-Toxic	Non-Toxic
Distance	0.632	0.707	0.714
Reference	Food Cosmet Toxicol 18(2):159-72; 1980	Chemotherapy 33:203-218; 1985	Teratology 15(3):231-41; 1977

Model Applicability

Unknown features are fingerprint features in the query molecule, but not found or appearing too infrequently in the training set.

1. All properties and OPS components are within expected ranges.

Feature Contribution

Top features for positive contribution				
Fingerprint	Bit/Smiles	Feature Structure	Score	Toxic in training set
SCFP_6	-1486266146	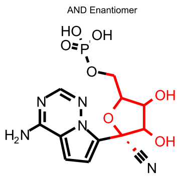 [*]CC1OC([*])([*])C(O)C1O	0.431	7 out of 8

SCFP_6	-1181430618	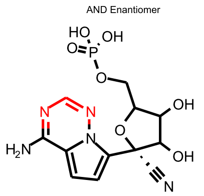 [*]:n:[cH]:n:[*]	0.298	6 out of 8
SCFP_6	-1715619483	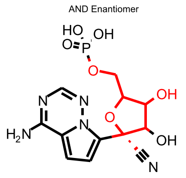 [*]OCC1OC([*])([*])C([*])C1O	0.298	6 out of 8

Top Features for negative contribution

Fingerprint	Bit/Smiles	Feature Structure	Score	Toxic in training set
SCFP_6	2108966103	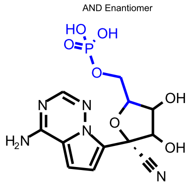 [*]C([*])COP(=O)(O)O	-0.945	0 out of 3
SCFP_6	-1375522316	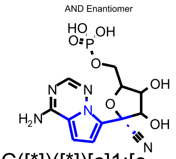 [*]C([*])([*])[c]1:[cH]:[cH]:[c](:[*]):n:1:[*]	-0.945	0 out of 3
SCFP_6	269938867	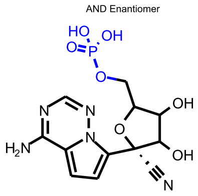 [*]OP(=O)(O)O	-0.729	1 out of 6

Flavonoid-1 TOPKAT_Mouse_Female_FDA_None_vs_Carcinogen

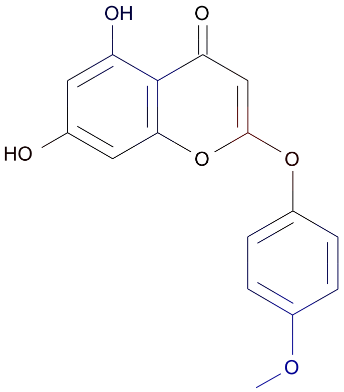

$C_{16}H_{12}O_6$

Molecular Weight: 300.26287

ALogP: 3.129

Rotatable Bonds: 3

Acceptors: 6

Donors: 2

Model Prediction

Prediction: Non-Carcinogen

Probability: 0.22

Enrichment: 0.685

Bayesian Score: -3.03

Mahalanobis Distance: 10.4

Mahalanobis Distance p-value: 0.265

Prediction: Positive if the Bayesian score is above the estimated best cutoff value from minimizing the false positive and false negative rate.
Probability: The esimated probability that the sample is in the positive category. This assumes that the Bayesian score follows a normal distribution and is different from the prediction using a cutoff.
Enrichment: An estimate of enrichment, that is, the increased likelihood (versus random) of this sample being in the category.
Bayesian Score: The standard Laplacian-modified Bayesian score.
Mahalanobis Distance: The Mahalanobis distance (MD) is the distance to the center of the training data. The larger the MD, the less trustworthy the prediction.
Mahalanobis Distance p-value: The p-value gives the fraction of training data with an MD greater than or equal to the one for the given sample, assuming normally distributed data. The smaller the p-value, the less trustworthy the prediciton. For highly non-normal X properties (e.g., fingerprints), the MD p-value is wildly inaccurate.

Structural Similar Compounds

Name	Niclosamide	Mebendazole	Cytembena
Structure	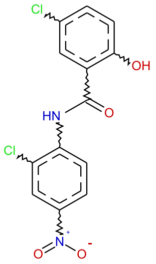	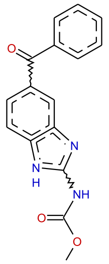	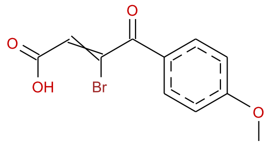
Actual Endpoint	Non-Carcinogen	Non-Carcinogen	Non-Carcinogen
Predicted Endpoint	Non-Carcinogen	Non-Carcinogen	Non-Carcinogen
Distance	0.639	0.640	0.656
Reference	US FDA (Centre for Drug Eval.& Res./Off. Testing & Res.) Sept. 1997	US FDA (Centre for Drug Eval.& Res./Off. Testing & Res.) Sept. 1997	US FDA (Centre for Drug Eval.& Res./Off. Testing & Res.) Sept. 1997

Model Applicability

Unknown features are fingerprint features in the query molecule, but not found or appearing too infrequently in the training set.

1. All properties and OPS components are within expected ranges.
2. Unknown ECFP_2 feature: 367973906: [*]OC(=C[*])O[*]

Feature Contribution

Top features for positive contribution

Fingerprint	Bit/Smiles	Feature Structure	Score	Carcinogen in training set
ECFP_6	464808839	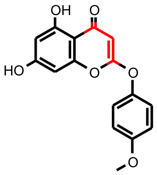 [*]C(=CC(=[*])[*])[*]	0.524	8 out of 14

ECFP_6	1305253718	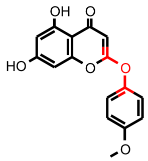 [*]C(=[*])O[c](:[*]):[*]	0.424	1 out of 1
ECFP_6	1407472008	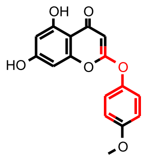 [*]C(=[*])O[c]1:[cH]:[cH]:[*]:[cH]:[cH]:1	0.424	1 out of 1

Top Features for negative contribution

Fingerprint	Bit/Smiles	Feature Structure	Score	Carcinogen in training set
ECFP_6	-1271104377	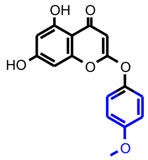 CO[c]1:[cH]:[cH]:[*]:[cH]:[cH]:1	-0.805	0 out of 4
ECFP_6	693720869	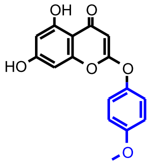 [*][c]1:[cH]:[cH]:[c](OC):[cH]:[cH]:1	-0.805	0 out of 4
ECFP_6	1307307440	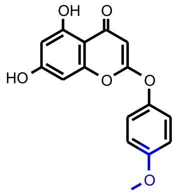 [*]:[c](:[*])OC	-0.558	4 out of 25

Flavonoid-2

TOPKAT_Mouse_Female_FDA_None_vs_Carcinogen

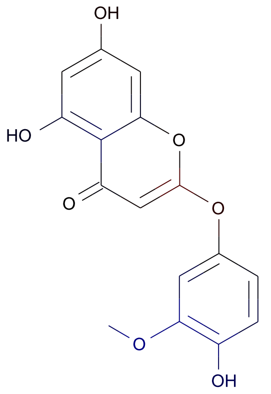

$C_{16}H_{12}O_7$
Molecular Weight: 316.26227
ALogP: 2.887
Rotatable Bonds: 3
Acceptors: 7
Donors: 3

Structural Similar Compounds

Name	Nedocromil	Olsalazine	Sulfasalazine
Structure	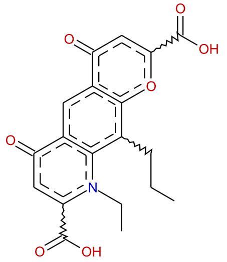	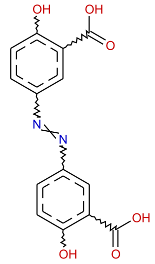	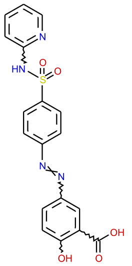
Actual Endpoint	Non-Carcinogen	Non-Carcinogen	Carcinogen
Predicted Endpoint	Non-Carcinogen	Carcinogen	Carcinogen
Distance	0.678	0.685	0.719
Reference	US FDA (Centre for Drug Eval.& Res./Off. Testing & Res.) Sept. 1997	US FDA (Centre for Drug Eval.& Res./Off. Testing & Res.) Sept. 1997	US FDA (Centre for Drug Eval.& Res./Off. Testing & Res.) Sept. 1997

Model Prediction

Prediction: Non-Carcinogen
Probability: 0.216
Enrichment: 0.674
Bayesian Score: -3.42
Mahalanobis Distance: 12.4
Mahalanobis Distance p-value: 0.00312

Prediction: Positive if the Bayesian score is above the estimated best cutoff value from minimizing the false positive and false negative rate.
Probability: The esimated probability that the sample is in the positive category. This assumes that the Bayesian score follows a normal distribution and is different from the prediction using a cutoff.
Enrichment: An estimate of enrichment, that is, the increased likelihood (versus random) of this sample being in the category.
Bayesian Score: The standard Laplacian-modified Bayesian score.
Mahalanobis Distance: The Mahalanobis distance (MD) is the distance to the center of the training data. The larger the MD, the less trustworthy the prediction.
Mahalanobis Distance p-value: The p-value gives the fraction of training data with an MD greater than or equal to the one for the given sample, assuming normally distributed data. The smaller the p-value, the less trustworthy the prediciton. For highly non-normal X properties (e.g., fingerprints), the MD p-value is wildly inaccurate.

Model Applicability

Unknown features are fingerprint features in the query molecule, but not found or appearing too infrequently in the training set.

1. All properties and OPS components are within expected ranges.
2. Unknown ECFP_2 feature: 367973906: [*]OC(=C[*])O[*]

Feature Contribution

Top features for positive contribution				
**Fingerprint**	**Bit/Smiles**	**Feature Structure**	**Score**	**Carcinogen in training set**
ECFP_6	464808839	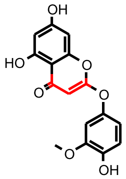 [*]C(=CC(=[*])[*])[*]	0.524	8 out of 14

ECFP_6	1305253718	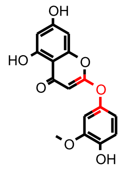 [*]C(=[*])O[c](:[*]):[*]	0.424	1 out of 1
ECFP_6	143734695	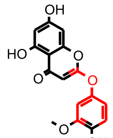 [*]C(=[*])O[c]1:[cH]:[cH]:[*]:[c]([*]):[cH]:1	0.424	1 out of 1

Top Features for negative contribution

Fingerprint	Bit/Smiles	Feature Structure	Score	Carcinogen in training set
ECFP_6	1307307440	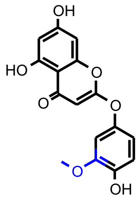 [*]:[c](:[*])OC	-0.558	4 out of 25
ECFP_6	1334400011	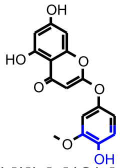 [*][c](:[*]):[c](O):[cH]:[*]	-0.496	3 out of 18
ECFP_6	864909220	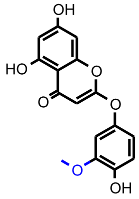 [*]OC	-0.466	7 out of 38

remdesivir

TOPKAT_Mouse_Female_FDA_None_vs_Carcinogen

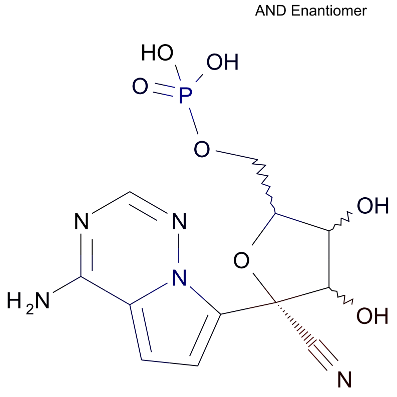

$C_{12}H_{14}N_5O_7P$
Molecular Weight: 371.24262
ALogP: -1.502
Rotatable Bonds: 4
Acceptors: 11
Donors: 5

Model Prediction

Prediction: Non-Carcinogen
Probability: 0.206
Enrichment: 0.642
Bayesian Score: -7.17
Mahalanobis Distance: 12.9
Mahalanobis Distance p-value: 0.00074

Prediction: Positive if the Bayesian score is above the estimated best cutoff value from minimizing the false positive and false negative rate.
Probability: The esimated probability that the sample is in the positive category. This assumes that the Bayesian score follows a normal distribution and is different from the prediction using a cutoff.
Enrichment: An estimate of enrichment, that is, the increased likelihood (versus random) of this sample being in the category.
Bayesian Score: The standard Laplacian-modified Bayesian score.
Mahalanobis Distance: The Mahalanobis distance (MD) is the distance to the center of the training data. The larger the MD, the less trustworthy the prediction.
Mahalanobis Distance p-value: The p-value gives the fraction of training data with an MD greater than or equal to the one for the given sample, assuming normally distributed data. The smaller the p-value, the less trustworthy the prediciton. For highly non-normal X properties (e.g., fingerprints), the MD p-value is wildly inaccurate.

Structural Similar Compounds

Name	Famotidine	Tetracycline	Oxytetracycline
Structure	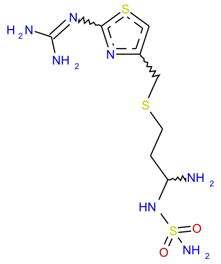	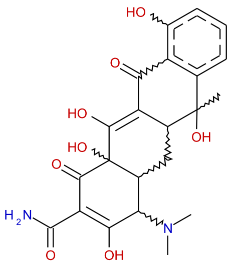	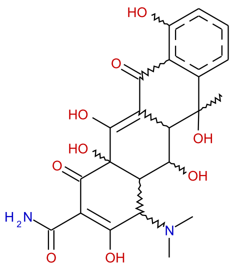
Actual Endpoint	Non-Carcinogen	Non-Carcinogen	Non-Carcinogen
Predicted Endpoint	Non-Carcinogen	Non-Carcinogen	Non-Carcinogen
Distance	0.846	0.848	0.870
Reference	US FDA (Centre for Drug Eval.& Res./Off. Testing & Res.) Sept. 1997	US FDA (Centre for Drug Eval.& Res./Off. Testing & Res.) Sept. 1997	US FDA (Centre for Drug Eval.& Res./Off. Testing & Res.) Sept. 1997

Model Applicability

Unknown features are fingerprint features in the query molecule, but not found or appearing too infrequently in the training set.

1. All properties and OPS components are within expected ranges.
2. Unknown ECFP_2 feature: 1126642748: [*]OP(=O)(O)O
3. Unknown ECFP_2 feature: -1250439909: [*]COP(=[*])([*])[*]
4. Unknown ECFP_2 feature: 1258791451: [*]C1[*][*]O[C@]1(C#[*])[c](:[*]):[*]
5. Unknown ECFP_2 feature: -1507082173: [*][c]1:[*]:[*]:[c](:[*]):n:1:n:[*]
6. Unknown ECFP_2 feature: -66263742: [*]C([*])([*])[c]1:[cH]:[*]:[*]:n:1:[*]

Feature Contribution

Top features for positive contribution				
Fingerprint	Bit/Smiles	Feature Structure	Score	Carcinogen in training set

ECFP_6	-1114776580	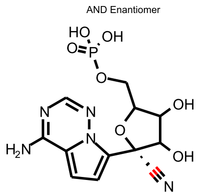 [*]C#[*]	0.755	11 out of 15
ECFP_6	-521596699	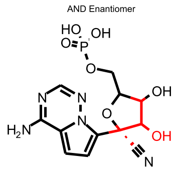 [*]C1[*][*]C([*])([*])C1O	0.451	3 out of 5
ECFP_6	-264833661	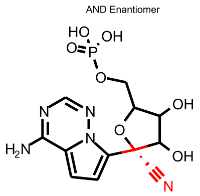 [*]C([*])([*])C#N	0.424	1 out of 1

Top Features for negative contribution

Fingerprint	Bit/Smiles	Feature Structure	Score	Carcinogen in training set
ECFP_6	1334415134	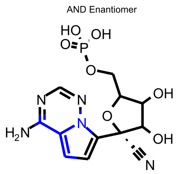 [*][c](:[*]):[c]1:[cH]:[*]:[*]:n:1:[*]	-0.935	0 out of 5
ECFP_6	2100964382	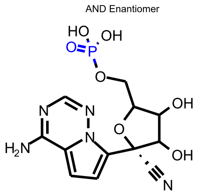 [*]P(=O)([*])[*]	-0.935	0 out of 5

ECFP_6	-826638028	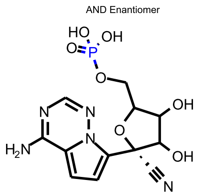 [*]P(=[*])([*])[*]	-0.935	0 out of 5

Flavonoid-1

TOPKAT_Mouse_Male_FDA_None_vs_Carcinogen

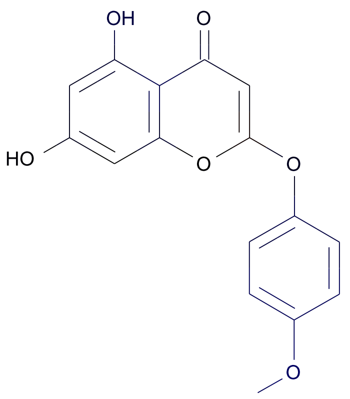

$C_{16}H_{12}O_6$

Molecular Weight: 300.26287

ALogP: 3.129

Rotatable Bonds: 3

Acceptors: 6

Donors: 2

Model Prediction

Prediction: Non-Carcinogen

Probability: 0.218

Enrichment: 0.739

Bayesian Score: -3.94

Mahalanobis Distance: 13.3

Mahalanobis Distance p-value: 0.000115

Prediction: Positive if the Bayesian score is above the estimated best cutoff value from minimizing the false positive and false negative rate.
Probability: The esimated probability that the sample is in the positive category. This assumes that the Bayesian score follows a normal distribution and is different from the prediction using a cutoff.
Enrichment: An estimate of enrichment, that is, the increased likelihood (versus random) of this sample being in the category.
Bayesian Score: The standard Laplacian-modified Bayesian score.
Mahalanobis Distance: The Mahalanobis distance (MD) is the distance to the center of the training data. The larger the MD, the less trustworthy the prediction.
Mahalanobis Distance p-value: The p-value gives the fraction of training data with an MD greater than or equal to the one for the given sample, assuming normally distributed data. The smaller the p-value, the less trustworthy the prediciton. For highly non-normal X properties (e.g., fingerprints), the MD p-value is wildly inaccurate.

Structural Similar Compounds

Name	Cytembena	Niclosamide	Mebendazole
Structure	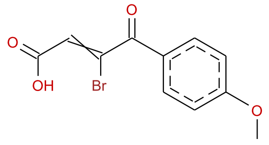	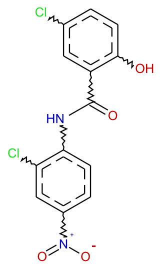	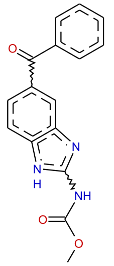
Actual Endpoint	Non-Carcinogen	Non-Carcinogen	Non-Carcinogen
Predicted Endpoint	Non-Carcinogen	Non-Carcinogen	Non-Carcinogen
Distance	0.614	0.616	0.639
Reference	US FDA (Centre for Drug Eval.& Res./Off. Testing & Res.) Sept. 1997	US FDA (Centre for Drug Eval.& Res./Off. Testing & Res.) Sept. 1997	US FDA (Centre for Drug Eval.& Res./Off. Testing & Res.) Sept. 1997

Model Applicability

Unknown features are fingerprint features in the query molecule, but not found or appearing too infrequently in the training set.

1. All properties and OPS components are within expected ranges.
2. Unknown FCFP_2 feature: -2115241127: [*]OC(=C[*])O[*]

Feature Contribution

Top features for positive contribution				
**Fingerprint**	**Bit/Smiles**	**Feature Structure**	**Score**	**Carcinogen in training set**
FCFP_6	451847724	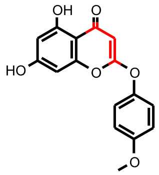 [*]C(=CC(=[*])[*])[*]	0.479	21 out of 48

FCFP_6	-158888774	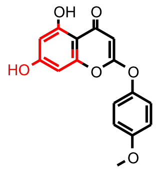 [*][c]1:[*]:[c]([*]):<br>[cH]:[c](O):[cH]:1	0.367	5 out of 12
FCFP_6	1872154524	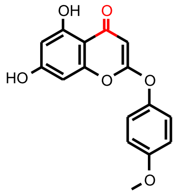 [*]C(=O)[*]	0.205	69 out of 213
**Top Features for negative contribution**				
**Fingerprint**	**Bit/Smiles**	**Feature Structure**	**Score**	**Carcinogen in training set**
FCFP_6	-9847677	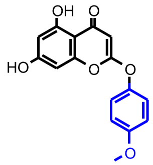 [*][c]1:[cH]:[cH]:[c]<br>(OC):[cH]:[cH]:1	-0.719	0 out of 4
FCFP_6	356782498	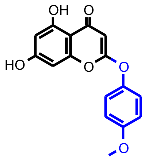 [*]O[c]1:[cH]:[cH]:[c<br>](OC):[cH]:[cH]:1	-0.582	0 out of 3
FCFP_6	-1549192822	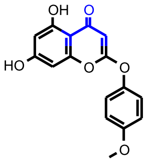 [*]=CC(=O)[c](:[*]):[<br>*]	-0.489	3 out of 21

Flavonoid-2

TOPKAT_Mouse_Male_FDA_None_vs_Carcinogen

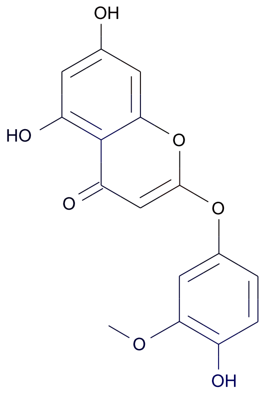

$C_{16}H_{12}O_7$
Molecular Weight: 316.26227
ALogP: 2.887
Rotatable Bonds: 3
Acceptors: 7
Donors: 3

Model Prediction

Prediction: Non-Carcinogen
Probability: 0.219
Enrichment: 0.743
Bayesian Score: -3.87
Mahalanobis Distance: 13.6
Mahalanobis Distance p-value: 3.49e-005

Prediction: Positive if the Bayesian score is above the estimated best cutoff value from minimizing the false positive and false negative rate.
Probability: The esimated probability that the sample is in the positive category. This assumes that the Bayesian score follows a normal distribution and is different from the prediction using a cutoff.
Enrichment: An estimate of enrichment, that is, the increased likelihood (versus random) of this sample being in the category.
Bayesian Score: The standard Laplacian-modified Bayesian score.
Mahalanobis Distance: The Mahalanobis distance (MD) is the distance to the center of the training data. The larger the MD, the less trustworthy the prediction.
Mahalanobis Distance p-value: The p-value gives the fraction of training data with an MD greater than or equal to the one for the given sample, assuming normally distributed data. The smaller the p-value, the less trustworthy the prediciton. For highly non-normal X properties (e.g., fingerprints), the MD p-value is wildly inaccurate.

Structural Similar Compounds

Name	Olsalazine	Nedocromil	Sulfasalazine
Structure	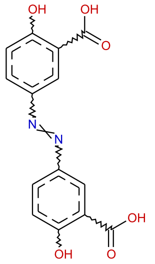	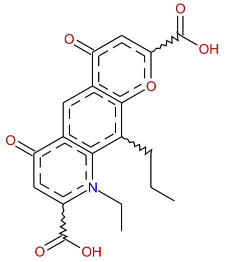	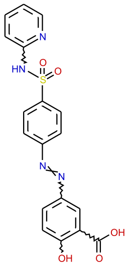
Actual Endpoint	Non-Carcinogen	Non-Carcinogen	Carcinogen
Predicted Endpoint	Carcinogen	Non-Carcinogen	Carcinogen
Distance	0.640	0.658	0.687
Reference	US FDA (Centre for Drug Eval.& Res./Off. Testing & Res.) Sept. 1997	US FDA (Centre for Drug Eval.& Res./Off. Testing & Res.) Sept. 1997	US FDA (Centre for Drug Eval.& Res./Off. Testing & Res.) Sept. 1997

Model Applicability

Unknown features are fingerprint features in the query molecule, but not found or appearing too infrequently in the training set.

1. All properties and OPS components are within expected ranges.
2. Unknown FCFP_2 feature: -2115241127: [*]OC(=C[*])O[*]

Feature Contribution

Top features for positive contribution

Fingerprint	Bit/Smiles	Feature Structure	Score	Carcinogen in training set
FCFP_6	451847724	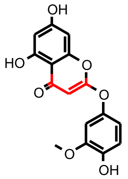 [*]C(=CC(=[*])[*])[*]	0.479	21 out of 48

FCFP_6	-158888774	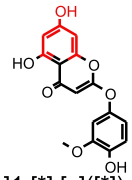 [*][c]1:[*]:[c]([*]):[cH]:[c](O):[cH]:1	0.367	5 out of 12
FCFP_6	1679744180	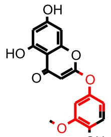 [*]O[c]1:[cH]:[*]:[c]([*]):[c](O[*]):[cH]:1	0.271	1 out of 2
**Top Features for negative contribution**				
**Fingerprint**	**Bit/Smiles**	**Feature Structure**	**Score**	**Carcinogen in training set**
FCFP_6	-1549192822	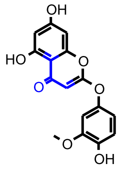 [*]=CC(=O)[c](:[*]):[*]	-0.489	3 out of 21
FCFP_6	-1604301295	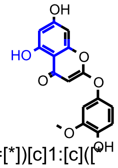 [*]C(=[*])[c]1:[c]([*]):[*]:[c]([*]):[cH]:[c]:1O	-0.445	2 out of 14
FCFP_6	523826990	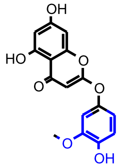 [*]O[c]1:[cH]:[*]:[cH]:[cH]:[c]:1O	-0.423	0 out of 2

remdesivir

TOPKAT_Mouse_Male_FDA_None_vs_Carcinogen

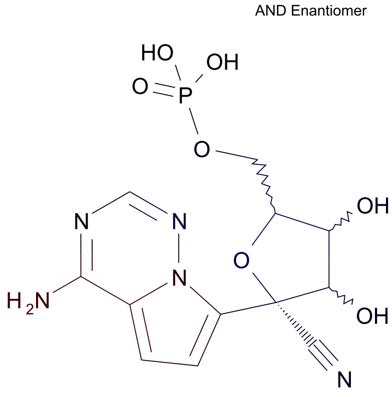

$C_{12}H_{14}N_5O_7P$
Molecular Weight: 371.24262
ALogP: -1.502
Rotatable Bonds: 4
Acceptors: 11
Donors: 5

Structural Similar Compounds

Name	Famotidine	Tetracycline	Ribavirin
Structure	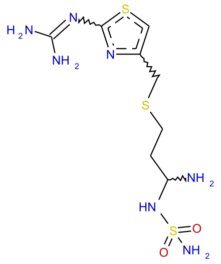	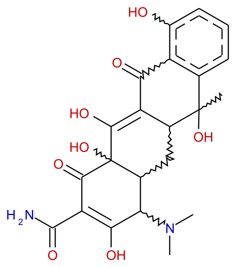	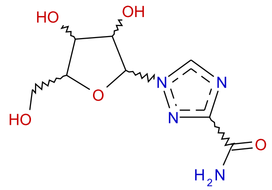
Actual Endpoint	Non-Carcinogen	Non-Carcinogen	Non-Carcinogen
Predicted Endpoint	Non-Carcinogen	Non-Carcinogen	Non-Carcinogen
Distance	0.813	0.843	0.860
Reference	US FDA (Centre for Drug Eval.& Res./Off. Testing & Res.) Sept. 1997	US FDA (Centre for Drug Eval.& Res./Off. Testing & Res.) Sept. 1997	US FDA (Centre for Drug Eval.& Res./Off. Testing & Res.) Sept. 1997

Model Prediction

Prediction: Non-Carcinogen
Probability: 0.239
Enrichment: 0.812
Bayesian Score: -2.82
Mahalanobis Distance: 19.2
Mahalanobis Distance p-value: 7.81e-017

Prediction: Positive if the Bayesian score is above the estimated best cutoff value from minimizing the false positive and false negative rate.
Probability: The esimated probability that the sample is in the positive category. This assumes that the Bayesian score follows a normal distribution and is different from the prediction using a cutoff.
Enrichment: An estimate of enrichment, that is, the increased likelihood (versus random) of this sample being in the category.
Bayesian Score: The standard Laplacian-modified Bayesian score.
Mahalanobis Distance: The Mahalanobis distance (MD) is the distance to the center of the training data. The larger the MD, the less trustworthy the prediction.
Mahalanobis Distance p-value: The p-value gives the fraction of training data with an MD greater than or equal to the one for the given sample, assuming normally distributed data. The smaller the p-value, the less trustworthy the prediciton. For highly non-normal X properties (e.g., fingerprints), the MD p-value is wildly inaccurate.

Model Applicability

Unknown features are fingerprint features in the query molecule, but not found or appearing too infrequently in the training set.

1. All properties and OPS components are within expected ranges.
2. Unknown FCFP_2 feature: 472180098: [*]OP(=O)(O)O
3. Unknown FCFP_2 feature: -332197802: [*][c]1:[*]:[*]:[c](:[*]):n:1:n:[*]

Feature Contribution

Top features for positive contribution

Fingerprint	Bit/Smiles	Feature Structure	Score	Carcinogen in training set
FCFP_6	-450797925	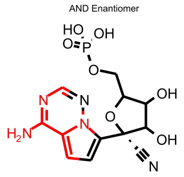 N[c]1:n:[cH]:[*]:n2:[*]:[*]:[cH]:[c]:1:2	0.676	2 out of 2

FCFP_6	-1151884458	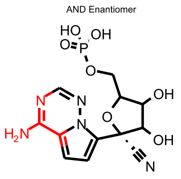 [*]:n:[c](N):[c](:[*]):[*]	0.348	6 out of 15
FCFP_6	-1280036918	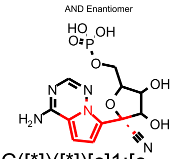 [*]C([*])([*])[c]1:[cH]:[cH]:[c](:[*]):n:1:[*]	0.333	7 out of 18
**Top Features for negative contribution**				
**Fingerprint**	**Bit/Smiles**	**Feature Structure**	**Score**	**Carcinogen in training set**
FCFP_6	-124685461	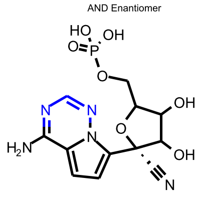 [*]:n:[cH]:n:[*]	-0.731	1 out of 12
FCFP_6	422052003	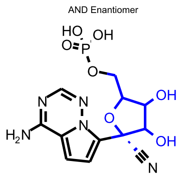 [*]CC1OC([*])([*])C(O)C1O	-0.582	0 out of 3
FCFP_6	-1277879912	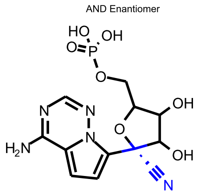 [*]C([*])([*])C#N	-0.582	0 out of 3

Flavonoid-1

TOPKAT_Ocular_Irritancy_Mild_vs_Moderate_Severe

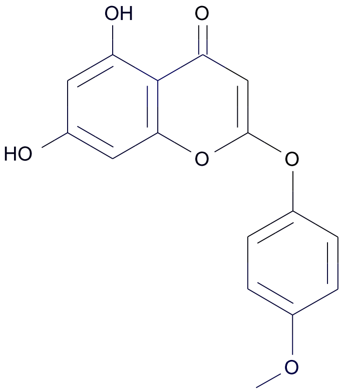

$C_{16}H_{12}O_6$
Molecular Weight: 300.26287
ALogP: 3.129
Rotatable Bonds: 3
Acceptors: 6
Donors: 2

Structural Similar Compounds

Name	ANTHRAQUINONE; 1-AMINO-4-HYDROXY-2-PHENOXY-	s-TRIAZINE; 2;4-BIS(ISOPROPYLAMINO0-6-(METHYLTHIO)-	s-TRIAZINE; 2-(tert-BUTYLAMINO)-4-(ETHYLAMINO)-6-(METHYLTHIO)-
Structure	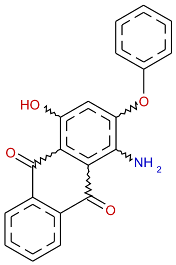	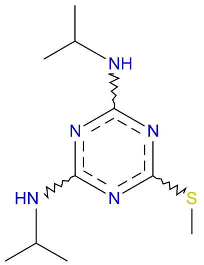	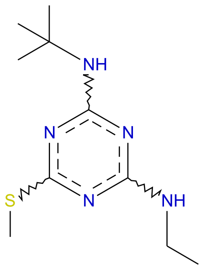
Actual Endpoint	Mild	Mild	Moderate_Severe
Predicted Endpoint	Mild	Mild	Moderate_Severe
Distance	0.555	0.587	0.591
Reference	28ZPAK 239;72	CIGET* -;-;77	CIGET* -;-;77

Model Prediction

Prediction: Mild
Probability: 0.756
Enrichment: 1.1
Bayesian Score: -2.35
Mahalanobis Distance: 7.66
Mahalanobis Distance p-value: 0.972

Prediction: Positive if the Bayesian score is above the estimated best cutoff value from minimizing the false positive and false negative rate.
Probability: The esimated probability that the sample is in the positive category. This assumes that the Bayesian score follows a normal distribution and is different from the prediction using a cutoff.
Enrichment: An estimate of enrichment, that is, the increased likelihood (versus random) of this sample being in the category.
Bayesian Score: The standard Laplacian-modified Bayesian score.
Mahalanobis Distance: The Mahalanobis distance (MD) is the distance to the center of the training data. The larger the MD, the less trustworthy the prediction.
Mahalanobis Distance p-value: The p-value gives the fraction of training data with an MD greater than or equal to the one for the given sample, assuming normally distributed data. The smaller the p-value, the less trustworthy the prediciton. For highly non-normal X properties (e.g., fingerprints), the MD p-value is wildly inaccurate.

Model Applicability

Unknown features are fingerprint features in the query molecule, but not found or appearing too infrequently in the training set.

1. All properties and OPS components are within expected ranges.
2. Unknown FCFP_2 feature: -2115241127: [*]OC(=C[*])O[*]

Feature Contribution

Top features for positive contribution

Fingerprint	Bit/Smiles	Feature Structure	Score	Moderate_Severe in training set
FCFP_10	-158888774	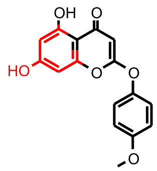 [*][c]1:[*]:[c]([*]):[cH]:[c](O):[cH]:1	0.356	24 out of 25

FCFP_10	7	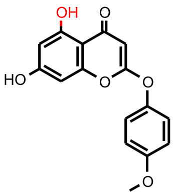 [*]O	0.219	117 out of 142
FCFP_10	346218766	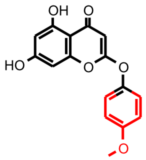 CO[c]1:[cH]:[cH]:[*]:[cH]:[cH]:1	0.197	30 out of 37
**Top Features for negative contribution**				
**Fingerprint**	**Bit/Smiles**	**Feature Structure**	**Score**	**Moderate_Severe in training set**
FCFP_10	-1977641857	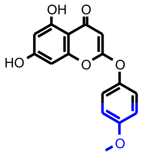 [*]:[cH]:[c](OC):[cH]:[*]	-0.78	4 out of 15
FCFP_10	115228054	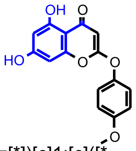 [*]C(=[*])[c]1:[c]([*]):[cH]:[c](O):[cH]:[c]:1O	-0.507	0 out of 1
FCFP_10	946068634	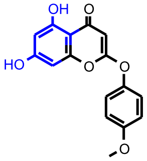 [*][c]1:[*]:[cH]:[c](O):[cH]:[c]:1O	-0.4	1 out of 3

Flavonoid-2 TOPKAT_Ocular_Irritancy_Mild_vs_Moderate_Severe

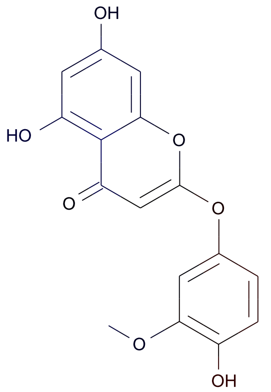

$C_{16}H_{12}O_7$
Molecular Weight: 316.26227
ALogP: 2.887
Rotatable Bonds: 3
Acceptors: 7
Donors: 3

Model Prediction

Prediction: Mild
Probability: 0.787
Enrichment: 1.14
Bayesian Score: -1.48
Mahalanobis Distance: 8.43
Mahalanobis Distance p-value: 0.792

Prediction: Positive if the Bayesian score is above the estimated best cutoff value from minimizing the false positive and false negative rate.
Probability: The esimated probability that the sample is in the positive category. This assumes that the Bayesian score follows a normal distribution and is different from the prediction using a cutoff.
Enrichment: An estimate of enrichment, that is, the increased likelihood (versus random) of this sample being in the category.
Bayesian Score: The standard Laplacian-modified Bayesian score.
Mahalanobis Distance: The Mahalanobis distance (MD) is the distance to the center of the training data. The larger the MD, the less trustworthy the prediction.
Mahalanobis Distance p-value: The p-value gives the fraction of training data with an MD greater than or equal to the one for the given sample, assuming normally distributed data. The smaller the p-value, the less trustworthy the prediciton. For highly non-normal X properties (e.g., fingerprints), the MD p-value is wildly inaccurate.

Structural Similar Compounds

**Name**	**ANTHRAQUINONE; 1;5-DIAMINO-4;8-DIHYDROXY-3-(p-METHOXYPHENYL)-**	**1;2;4-TRIHYDROXY ANTHRAQUINONE**	**2-NAPHTHALENESULFONIC ACID; 4-HYDROXY-7-(METHYLAMINO)-**
Structure	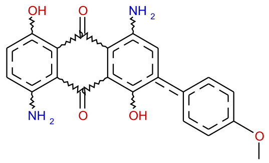	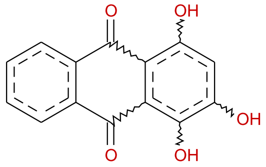	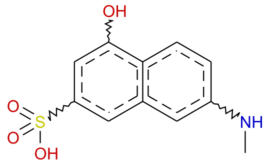
Actual Endpoint	Mild	Mild	Mild
Predicted Endpoint	Mild	Mild	Mild
Distance	0.689	0.694	0.715
Reference	28ZPAK 245;72	28ZPAK-;103;7	28ZPAK 190;72

Model Applicability

Unknown features are fingerprint features in the query molecule, but not found or appearing too infrequently in the training set.

1. All properties and OPS components are within expected ranges.
2. Unknown FCFP_2 feature: -2115241127: [*]OC(=C[*])O[*]

Feature Contribution

Top features for positive contribution

Fingerprint	Bit/Smiles	Feature Structure	Score	Moderate_Severe in training set
FCFP_10	-158888774	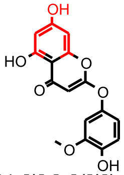 [*][c]1:[*]:[c]([*]):[cH]:[c](O):[cH]:1	0.356	24 out of 25

FCFP_10	1679744180	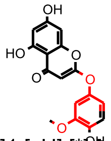 [*]O[c]1:[cH]:[*]:[c]([*]):[c](O[*]):[cH]:1	0.256	2 out of 2
FCFP_10	7	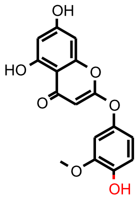 [*]O	0.219	117 out of 142

Top Features for negative contribution

**Fingerprint**	**Bit/Smiles**	**Feature Structure**	**Score**	**Moderate_Severe in training set**
FCFP_10	-1977641857	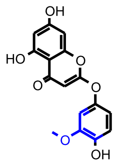 [*]:[cH]:[c](OC):[cH]:[*]	-0.78	4 out of 15
FCFP_10	115228054	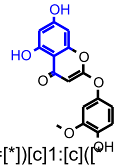 [*]C(=[*])[c]1:[c]([*]):[cH]:[c](O):[cH]:[c]:1O	-0.507	0 out of 1
FCFP_10	946068634	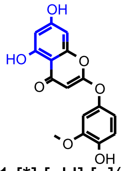 [*][c]1:[*]:[cH]:[c](O):[cH]:[c]:1O	-0.4	1 out of 3

remdesivir

TOPKAT_Ocular_Irritancy_Mild_vs_Moderate_Severe

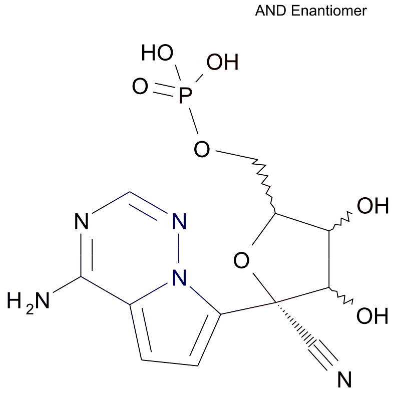

$C_{12}H_{14}N_5O_7P$

Molecular Weight: 371.24262

ALogP: -1.502

Rotatable Bonds: 4

Acceptors: 11

Donors: 5

Structural Similar Compounds

Name	1;3;6-NAPHTHALENE TRISULFONIC ACID;7-AMINO-	Methanol; (s-triazine-2;4;6-triyltrinitrilo)hexa-	2;2'-Biphenyldisulfonic acid; 4;4'-diamino-
Structure	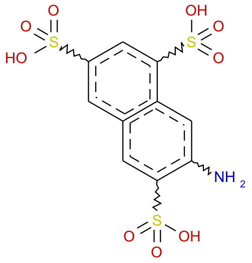	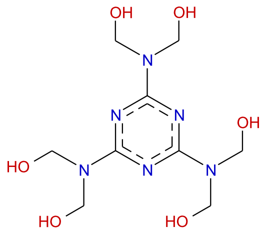	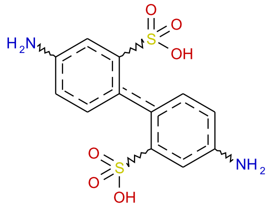
Actual Endpoint	Mild	Moderate_Severe	Mild
Predicted Endpoint	Mild	Moderate_Severe	Mild
Distance	0.776	0.802	0.878
Reference	28ZPAK-;190;72	Prehled Prumyslove Toxikologie; Organicke Latky; Marhold; J. -;876;86	Prehled Prumyslove Toxikologie; Organicke Latky; Marhold; J. pp 1061;86

Model Prediction

Prediction: Mild

Probability: 0.789

Enrichment: 1.15

Bayesian Score: -1.39

Mahalanobis Distance: 13.8

Mahalanobis Distance p-value: 1.42e-009

Prediction: Positive if the Bayesian score is above the estimated best cutoff value from minimizing the false positive and false negative rate.
Probability: The esimated probability that the sample is in the positive category. This assumes that the Bayesian score follows a normal distribution and is different from the prediction using a cutoff.
Enrichment: An estimate of enrichment, that is, the increased likelihood (versus random) of this sample being in the category.
Bayesian Score: The standard Laplacian-modified Bayesian score.
Mahalanobis Distance: The Mahalanobis distance (MD) is the distance to the center of the training data. The larger the MD, the less trustworthy the prediction.
Mahalanobis Distance p-value: The p-value gives the fraction of training data with an MD greater than or equal to the one for the given sample, assuming normally distributed data. The smaller the p-value, the less trustworthy the prediciton. For highly non-normal X properties (e.g., fingerprints), the MD p-value is wildly inaccurate.

Model Applicability

Unknown features are fingerprint features in the query molecule, but not found or appearing too infrequently in the training set.

1. OPS PC17 out of range. Value: 4.6782. Training min, max, SD, explained variance: -4.348, 3.9505, 1.094, 0.0146.
2. Unknown FCFP_2 feature: 472180098: [*]OP(=O)(O)O
3. Unknown FCFP_2 feature: -836603894: [*]C1[*][*]O[C@]1(C#[*])[c](:[*]):[*]
4. Unknown FCFP_2 feature: -124685461: [*]:n:[cH]:n:[*]
5. Unknown FCFP_2 feature: -1151884458: [*]:n:[c](N):[c](:[*]):[*]

Feature Contribution

Top features for positive contribution

Fingerprint	Bit/Smiles	Feature Structure	Score	Moderate_Severe in training set

FCFP_10	1070061035	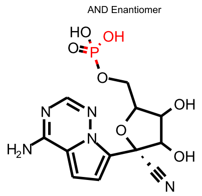 [*]P(=[*])([*])O	0.239	284 out of 338
FCFP_10	-1539132615	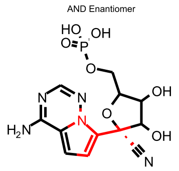 [*]C([*])([*])[c]1:[cH]:[*]:[*]:n:1:[*]	0.224	11 out of 13
FCFP_10	-1043250487	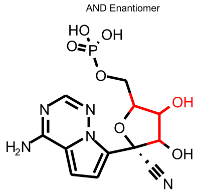 [*]C1[*][*]C([*])C1O	0.22	62 out of 75

Top Features for negative contribution

Fingerprint	Bit/Smiles	Feature Structure	Score	Moderate_Severe in training set
FCFP_10	4427049	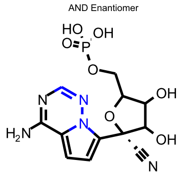 [*]:[cH]:n:n(:[*]):[*]	-1.29	0 out of 4
FCFP_10	-332197802	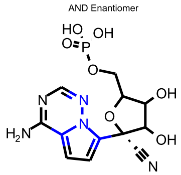 [*][c]1:[*]:[*]:[c](:[*]):n:1:n:[*]	-0.507	0 out of 1

FCFP_10	713358128	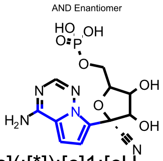 [*][c](:[*]):[c]1:[cH]:[cH]:[c]([*]):n:1:[*]	-0.307	8 out of 17

Flavonoid-1

TOPKAT_Ocular_Irritancy_None_vs_Irritant

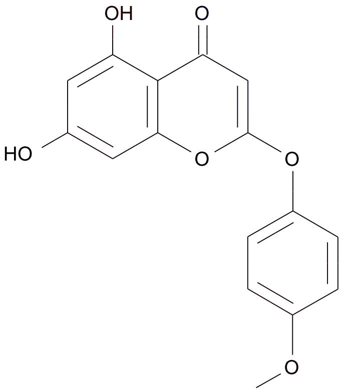

$C_{16}H_{12}O_6$

Molecular Weight: 300.26287

ALogP: 3.129

Rotatable Bonds: 3

Acceptors: 6

Donors: 2

Structural Similar Compounds

Name	2;2';-Dihydroxy-4;4'-dimethoxybenzophenone	ANTHRAQUINONE; 1-AMINO-4-HYDROXY-2-PHENOXY-	s-TRIAZINE; 2;4-BIS(ISOPROPYLAMINO0-6-(METHYLTHIO)-
Structure	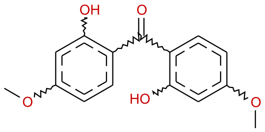	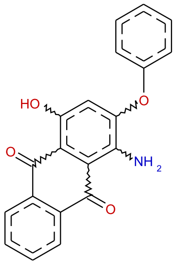	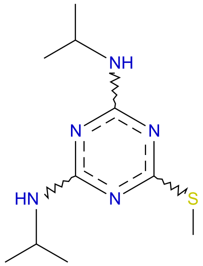
Actual Endpoint	Non-Irritant	Irritant	Irritant
Predicted Endpoint	Non-Irritant	Irritant	Irritant
Distance	0.514	0.553	0.581
Reference	J. Am. Coll. Toxicol. 2(5):35;1983	28ZPAK 239;72	CIGET* -;-;77

Model Prediction

Prediction: Irritant

Probability: 1

Enrichment: 1.18

Bayesian Score: 0.451

Mahalanobis Distance: 7.1

Mahalanobis Distance p-value: 0.997

Prediction: Positive if the Bayesian score is above the estimated best cutoff value from minimizing the false positive and false negative rate.
Probability: The esimated probability that the sample is in the positive category. This assumes that the Bayesian score follows a normal distribution and is different from the prediction using a cutoff.
Enrichment: An estimate of enrichment, that is, the increased likelihood (versus random) of this sample being in the category.
Bayesian Score: The standard Laplacian-modified Bayesian score.
Mahalanobis Distance: The Mahalanobis distance (MD) is the distance to the center of the training data. The larger the MD, the less trustworthy the prediction.
Mahalanobis Distance p-value: The p-value gives the fraction of training data with an MD greater than or equal to the one for the given sample, assuming normally distributed data. The smaller the p-value, the less trustworthy the prediciton. For highly non-normal X properties (e.g., fingerprints), the MD p-value is wildly inaccurate.

Model Applicability

Unknown features are fingerprint features in the query molecule, but not found or appearing too infrequently in the training set.

1. All properties and OPS components are within expected ranges.
2. Unknown FCFP_2 feature: -2115241127: [*]OC(=C[*])O[*]

Feature Contribution

Top features for positive contribution

Fingerprint	Bit/Smiles	Feature Structure	Score	Irritant in training set
FCFP_12	-1099193755	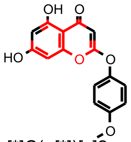 [*]C1=[*]C(=[*])[c]2:[c]([*]):[*]:[c]([*]):[cH]:[c]:2O1	0.175	5 out of 5

FCFP_12	946068634	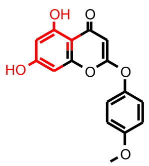 [*][c]1:[*]:[cH]:[c](O):[cH]:[c]:1O	0.156	3 out of 3
FCFP_12	-9847677	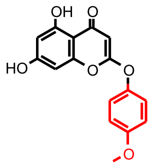 [*][c]1:[cH]:[cH]:[c](OC):[cH]:[cH]:1	0.156	3 out of 3

Top Features for negative contribution

Fingerprint	Bit/Smiles	Feature Structure	Score	Irritant in training set
FCFP_12	1244036906	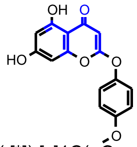 [*][c](:[*]):[c]1C(=O)C=C([*])[*][c]:1:[*]	-0.592	0 out of 1
FCFP_12	1673930087	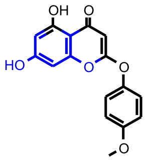 [*]O[c]1:[cH]:[c](O):[cH]:[*]:[c]:1[*]	-0.218	5 out of 8
FCFP_12	136627117	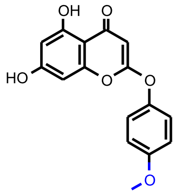 [*]OC	0	96 out of 113

Flavonoid-2

TOPKAT_Ocular_Irritancy_None_vs_Irritant

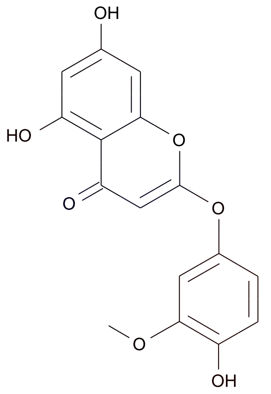

$C_{16}H_{12}O_7$

Molecular Weight: 316.26227

ALogP: 2.887

Rotatable Bonds: 3

Acceptors: 7

Donors: 3

Structural Similar Compounds

Name	Disperse Black 9	1;2;4-TRIHYDROXY ANTHRAQUINONE	ANTHRAQUINONE; 1;5-DIAMINO-4;8-DIHYDROXY-3-(p-METHOXYPHENYL)-
Structure	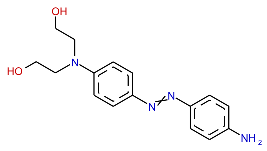	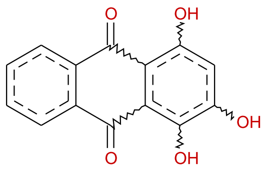	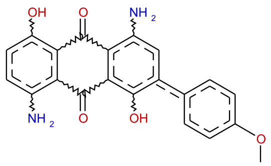
Actual Endpoint	Non-Irritant	Irritant	Irritant
Predicted Endpoint	Non-Irritant	Irritant	Irritant
Distance	0.645	0.682	0.688
Reference	J. Am. Coll. Toxicol. 5(3):205;1986	28ZPAK-;103;7	28ZPAK 245;72

Model Prediction

Prediction: Irritant

Probability: 1

Enrichment: 1.18

Bayesian Score: 0.552

Mahalanobis Distance: 8.09

Mahalanobis Distance p-value: 0.905

Prediction: Positive if the Bayesian score is above the estimated best cutoff value from minimizing the false positive and false negative rate.
Probability: The esimated probability that the sample is in the positive category. This assumes that the Bayesian score follows a normal distribution and is different from the prediction using a cutoff.
Enrichment: An estimate of enrichment, that is, the increased likelihood (versus random) of this sample being in the category.
Bayesian Score: The standard Laplacian-modified Bayesian score.
Mahalanobis Distance: The Mahalanobis distance (MD) is the distance to the center of the training data. The larger the MD, the less trustworthy the prediction.
Mahalanobis Distance p-value: The p-value gives the fraction of training data with an MD greater than or equal to the one for the given sample, assuming normally distributed data. The smaller the p-value, the less trustworthy the prediciton. For highly non-normal X properties (e.g., fingerprints), the MD p-value is wildly inaccurate.

Model Applicability

Unknown features are fingerprint features in the query molecule, but not found or appearing too infrequently in the training set.

1. All properties and OPS components are within expected ranges.
2. Unknown FCFP_2 feature: -2115241127: [*]OC(=C[*])O[*]

Feature Contribution

Top features for positive contribution				
**Fingerprint**	**Bit/Smiles**	**Feature Structure**	**Score**	**Irritant in training set**
FCFP_12	-1099193755	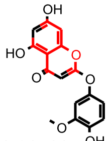 [*]C1=[*]C(=[*])[c]2:[c]([*]):[*]:[c]([*]):[cH]:[c]:2O1	0.175	5 out of 5

FCFP_12	946068634	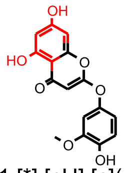 [*][c]1:[*]:[cH]:[c](O):[cH]:[c]:1O	0.156	3 out of 3
FCFP_12	1679744180	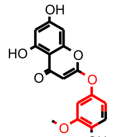 [*]O[c]1:[cH]:[*]:[c]([*]):[c](O[*]):[cH]:1	0.137	2 out of 2

Top Features for negative contribution

**Fingerprint**	**Bit/Smiles**	**Feature Structure**	**Score**	**Irritant in training set**
FCFP_12	1244036906	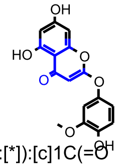 [*][c](:[*]):[c]1C(=O)C=C([*])[*][c]:1:[*]	-0.592	0 out of 1
FCFP_12	1673930087	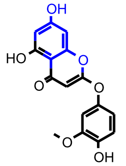 [*]O[c]1:[cH]:[c](O):[cH]:[*]:[c]:1[*]	-0.218	5 out of 8
FCFP_12	-1977641857	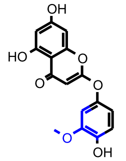 [*]:[cH]:[c](OC):[cH]:[*]	0	15 out of 19

remdesivir

TOPKAT_Ocular_Irritancy_None_vs_Irritant

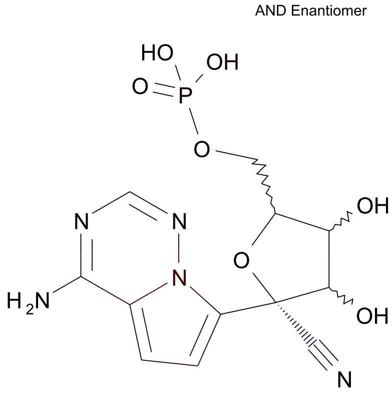

$C_{12}H_{14}N_5O_7P$
Molecular Weight: 371.24262
ALogP: -1.502
Rotatable Bonds: 4
Acceptors: 11
Donors: 5

Structural Similar Compounds

Name	1;3;6-NAPHTHALENE TRISULFONIC ACID;7-AMINO-	Methanol; (s-triazine-2;4;6-triyltrinitrilo)hexa-	2;2'-Biphenyldisulfonic acid; 4;4'-diamino-
Structure	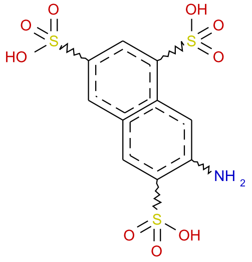	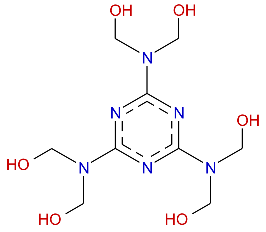	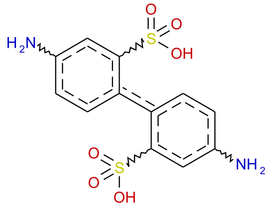
Actual Endpoint	Irritant	Irritant	Irritant
Predicted Endpoint	Irritant	Irritant	Irritant
Distance	0.766	0.795	0.859
Reference	28ZPAK-;190;72	Prehled Prumyslove Toxikologie; Organicke Latky; Marhold; J. -;876;86	Prehled Prumyslove Toxikologie; Organicke Latky; Marhold; J. pp 1061;86

Model Prediction

Prediction: Irritant
Probability: 1
Enrichment: 1.18
Bayesian Score: 1.33
Mahalanobis Distance: 10.7
Mahalanobis Distance p-value: 0.0147

Prediction: Positive if the Bayesian score is above the estimated best cutoff value from minimizing the false positive and false negative rate.
Probability: The esimated probability that the sample is in the positive category. This assumes that the Bayesian score follows a normal distribution and is different from the prediction using a cutoff.
Enrichment: An estimate of enrichment, that is, the increased likelihood (versus random) of this sample being in the category.
Bayesian Score: The standard Laplacian-modified Bayesian score.
Mahalanobis Distance: The Mahalanobis distance (MD) is the distance to the center of the training data. The larger the MD, the less trustworthy the prediction.
Mahalanobis Distance p-value: The p-value gives the fraction of training data with an MD greater than or equal to the one for the given sample, assuming normally distributed data. The smaller the p-value, the less trustworthy the prediciton. For highly non-normal X properties (e.g., fingerprints), the MD p-value is wildly inaccurate.

Model Applicability

Unknown features are fingerprint features in the query molecule, but not found or appearing too infrequently in the training set.

1. All properties and OPS components are within expected ranges.
2. Unknown FCFP_2 feature: 472180098: [*]OP(=O)(O)O
3. Unknown FCFP_2 feature: -124685461: [*]:n:[cH]:n:[*]
4. Unknown FCFP_2 feature: -1151884458: [*]:n:[c](N):[c](:[*]):[*]

Feature Contribution

Top features for positive contribution

Fingerprint	Bit/Smiles	Feature Structure	Score	Irritant in training set

FCFP_12	1747237384	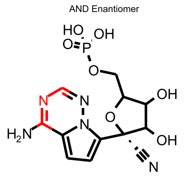<br>[*][c](:[*]):n:[cH]:[*]	0.208	44 out of 44
FCFP_12	178336375	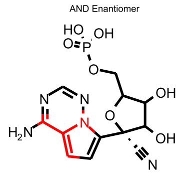<br>[*][c](:[*]):[c]1:[cH]:[*]:[*]:n:1:[*]	0.202	19 out of 19
FCFP_12	713358128	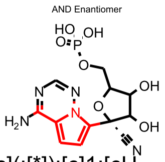<br>[*][c](:[*]):[c]1:[cH]:[cH]:[c]([*]):n:1:[*]	0.2	17 out of 17

Top Features for negative contribution

Fingerprint	Bit/Smiles	Feature Structure	Score	Irritant in training set
FCFP_12	-836603894	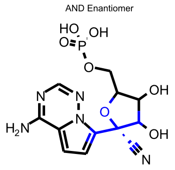<br>[*]C1[*][*]O[C@]1(C#[*])[c](:[*]):[*]	-0.592	0 out of 1
FCFP_12	-1277879912	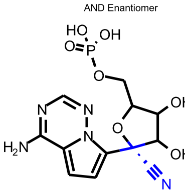<br>[*]C([*])([*])C#N	-0.0939	33 out of 45

FCFP_12	-1272768868	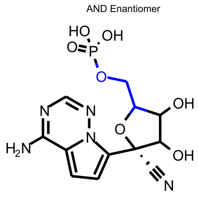<br>[*]OCC([*])[*]	0	396 out of 514

Flavonoid-1

TOPKAT_Rat_Female_FDA_None_vs_Carcinogen

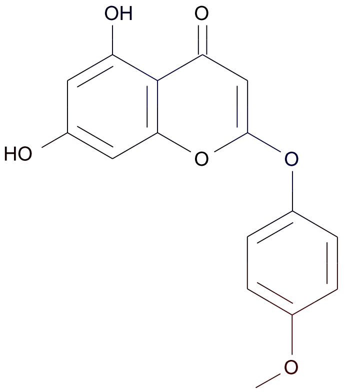

$C_{16}H_{12}O_6$

Molecular Weight: 300.26287

ALogP: 3.129

Rotatable Bonds: 3

Acceptors: 6

Donors: 2

Model Prediction

Prediction: Carcinogen

Probability: 0.285

Enrichment: 0.886

Bayesian Score: -0.681

Mahalanobis Distance: 9.5

Mahalanobis Distance p-value: 0.607

Prediction: Positive if the Bayesian score is above the estimated best cutoff value from minimizing the false positive and false negative rate.
Probability: The esimated probability that the sample is in the positive category. This assumes that the Bayesian score follows a normal distribution and is different from the prediction using a cutoff.
Enrichment: An estimate of enrichment, that is, the increased likelihood (versus random) of this sample being in the category.
Bayesian Score: The standard Laplacian-modified Bayesian score.
Mahalanobis Distance: The Mahalanobis distance (MD) is the distance to the center of the training data. The larger the MD, the less trustworthy the prediction.
Mahalanobis Distance p-value: The p-value gives the fraction of training data with an MD greater than or equal to the one for the given sample, assuming normally distributed data. The smaller the p-value, the less trustworthy the prediciton. For highly non-normal X properties (e.g., fingerprints), the MD p-value is wildly inaccurate.

Structural Similar Compounds

Name	Niclosamide	Mebendazole	Estrogens; conjug.
Structure	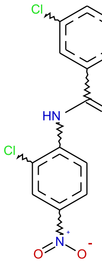	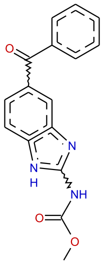	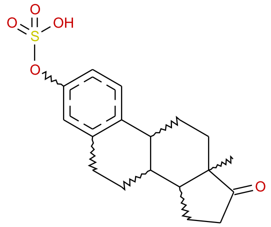
Actual Endpoint	Non-Carcinogen	Non-Carcinogen	Carcinogen
Predicted Endpoint	Non-Carcinogen	Non-Carcinogen	Carcinogen
Distance	0.658	0.661	0.667
Reference	US FDA (Centre for Drug Eval.& Res./Off. Testing & Res.) Sept. 1997	US FDA (Centre for Drug Eval.& Res./Off. Testing & Res.) Sept. 1997	US FDA (Centre for Drug Eval.& Res./Off. Testing & Res.) Sept. 1997

Model Applicability

Unknown features are fingerprint features in the query molecule, but not found or appearing too infrequently in the training set.

1. All properties and OPS components are within expected ranges.
2. Unknown ECFP_2 feature: 367973906: [*]OC(=C[*])O[*]

Feature Contribution

Top features for positive contribution

Fingerprint	Bit/Smiles	Feature Structure	Score	Carcinogen in training set
ECFP_12	-177786161	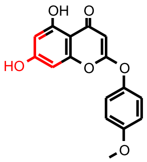 [*]:[cH]:[c](O):[cH]:[*]	0.341	7 out of 15

ECFP_12	693720869	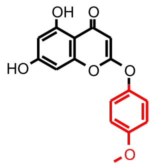<br>[*][c]1:[cH]:[cH]:[c]<br>(OC):[cH]:[cH]:1	0.33	3 out of 6
ECFP_12	-1271104377	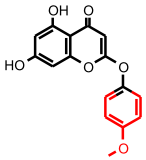<br>CO[c]1:[cH]:[cH]:[*]:<br>[cH]:[cH]:1	0.33	3 out of 6
**Top Features for negative contribution**				
**Fingerprint**	**Bit/Smiles**	**Feature Structure**	**Score**	**Carcinogen in training set**
ECFP_12	1305253718	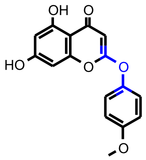<br>[*]C(=[*])O[c](:[*]):<br>[*]	-0.485	0 out of 2
ECFP_12	1407472008	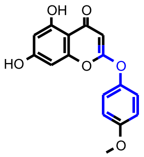<br>[*]C(=[*])O[c]1:[cH]:<br>[cH]:[*]:[cH]:[cH]:1	-0.485	0 out of 2
ECFP_12	1299558496	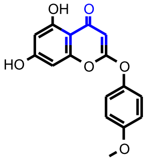<br>[*]=CC(=O)[c](:[*]):[<br>*]	-0.272	0 out of 1

Flavonoid-2 TOPKAT_Rat_Female_FDA_None_vs_Carcinogen

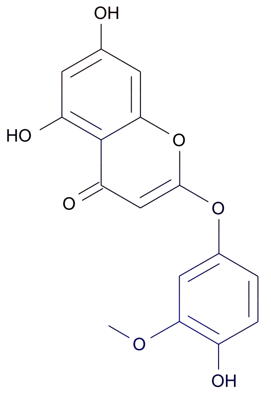

$C_{16}H_{12}O_7$

Molecular Weight: 316.26227

ALogP: 2.887

Rotatable Bonds: 3

Acceptors: 7

Donors: 3

Model Prediction

Prediction: Non-Carcinogen

Probability: 0.241

Enrichment: 0.75

Bayesian Score: -3.38

Mahalanobis Distance: 11.8

Mahalanobis Distance p-value: 0.00808

Prediction: Positive if the Bayesian score is above the estimated best cutoff value from minimizing the false positive and false negative rate.
Probability: The esimated probability that the sample is in the positive category. This assumes that the Bayesian score follows a normal distribution and is different from the prediction using a cutoff.
Enrichment: An estimate of enrichment, that is, the increased likelihood (versus random) of this sample being in the category.
Bayesian Score: The standard Laplacian-modified Bayesian score.
Mahalanobis Distance: The Mahalanobis distance (MD) is the distance to the center of the training data. The larger the MD, the less trustworthy the prediction.
Mahalanobis Distance p-value: The p-value gives the fraction of training data with an MD greater than or equal to the one for the given sample, assuming normally distributed data. The smaller the p-value, the less trustworthy the prediciton. For highly non-normal X properties (e.g., fingerprints), the MD p-value is wildly inaccurate.

Structural Similar Compounds

Name	Clorazepate	Nedocromil	Olsalazine
Structure	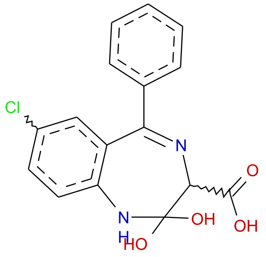	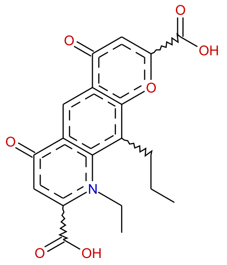	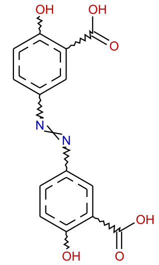
Actual Endpoint	Non-Carcinogen	Non-Carcinogen	Non-Carcinogen
Predicted Endpoint	Non-Carcinogen	Non-Carcinogen	Non-Carcinogen
Distance	0.664	0.695	0.700
Reference	US FDA (Centre for Drug Eval.& Res./Off. Testing & Res.) Sept. 1997	US FDA (Centre for Drug Eval.& Res./Off. Testing & Res.) Sept. 1997	US FDA (Centre for Drug Eval.& Res./Off. Testing & Res.) Sept. 1997

Model Applicability

Unknown features are fingerprint features in the query molecule, but not found or appearing too infrequently in the training set.

1. All properties and OPS components are within expected ranges.
2. Unknown ECFP_2 feature: 367973906: [*]OC(=C[*])O[*]

Feature Contribution

Top features for positive contribution				
**Fingerprint**	**Bit/Smiles**	**Feature Structure**	**Score**	**Carcinogen in training set**
ECFP_12	-177786161	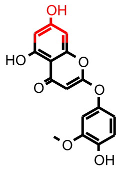 [*]:[cH]:[c](O):[cH]:[*]	0.341	7 out of 15

ECFP_12	683445015	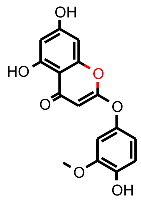 [*]O[*]	0.294	28 out of 66
ECFP_12	478798802	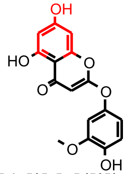 [*][c]1:[*]:[c]([*]):[cH]:[c](O):[cH]:1	0.208	1 out of 2

Top Features for negative contribution

Fingerprint	Bit/Smiles	Feature Structure	Score	Carcinogen in training set
ECFP_12	1408898974	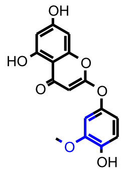 [*]O[c](:[cH]:[*]):[c]([*]):[*]	-0.517	5 out of 29
ECFP_12	1305253718	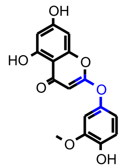 [*]C(=[*])O[c](:[*]):[*]	-0.485	0 out of 2
ECFP_12	1680623188	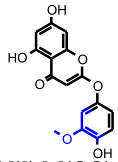 [*][c](:[*]):[c](OC):[cH]:[*]	-0.295	3 out of 14

remdesivir

TOPKAT_Rat_Female_FDA_None_vs_Carcinogen

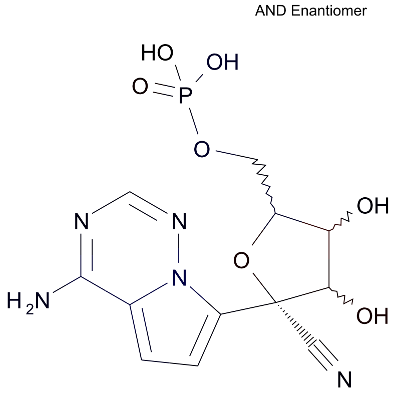

$C_{12}H_{14}N_5O_7P$
Molecular Weight: 371.24262
ALogP: -1.502
Rotatable Bonds: 4
Acceptors: 11
Donors: 5

Model Prediction

Prediction: Non-Carcinogen
Probability: 0.243
Enrichment: 0.756
Bayesian Score: -3.24
Mahalanobis Distance: 13.8
Mahalanobis Distance p-value: 5.04e-006

Prediction: Positive if the Bayesian score is above the estimated best cutoff value from minimizing the false positive and false negative rate.
Probability: The esimated probability that the sample is in the positive category. This assumes that the Bayesian score follows a normal distribution and is different from the prediction using a cutoff.
Enrichment: An estimate of enrichment, that is, the increased likelihood (versus random) of this sample being in the category.
Bayesian Score: The standard Laplacian-modified Bayesian score.
Mahalanobis Distance: The Mahalanobis distance (MD) is the distance to the center of the training data. The larger the MD, the less trustworthy the prediction.
Mahalanobis Distance p-value: The p-value gives the fraction of training data with an MD greater than or equal to the one for the given sample, assuming normally distributed data. The smaller the p-value, the less trustworthy the prediciton. For highly non-normal X properties (e.g., fingerprints), the MD p-value is wildly inaccurate.

Structural Similar Compounds

Name	Streptozocin	Tetracycline	Famotidine
Structure	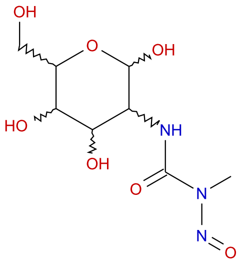	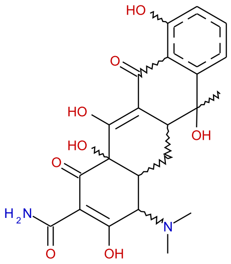	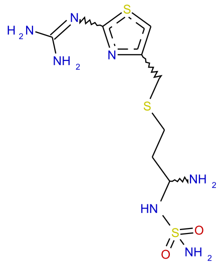
Actual Endpoint	Carcinogen	Non-Carcinogen	Non-Carcinogen
Predicted Endpoint	Carcinogen	Non-Carcinogen	Non-Carcinogen
Distance	0.810	0.858	0.861
Reference	US FDA (Centre for Drug Eval.& Res./Off. Testing & Res.) Sept. 1997	US FDA (Centre for Drug Eval.& Res./Off. Testing & Res.) Sept. 1997	US FDA (Centre for Drug Eval.& Res./Off. Testing & Res.) Sept. 1997

Model Applicability

Unknown features are fingerprint features in the query molecule, but not found or appearing too infrequently in the training set.

1. All properties and OPS components are within expected ranges.
2. Unknown ECFP_2 feature: 1126642748: [*]OP(=O)(O)O
3. Unknown ECFP_2 feature: -1250439909: [*]COP(=[*])([*])[*]
4. Unknown ECFP_2 feature: 1258791451: [*]C1[*][*]O[C@]1(C#[*])[c](:[*]):[*]
5. Unknown ECFP_2 feature: -1507082173: [*][c]1:[*]:[*]:[c](:[*]):n:1:n:[*]
6. Unknown ECFP_2 feature: -66263742: [*]C([*])([*])[c]1:[cH]:[*]:[*]:n:1:[*]

Feature Contribution

Top features for positive contribution

Fingerprint	Bit/Smiles	Feature Structure	Score	Carcinogen in training set

ECFP_12	-553149446	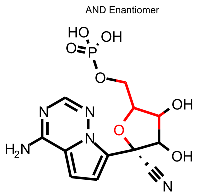 [*]CC1O[*][*]C1[*]	0.575	3 out of 4
ECFP_12	-1114776580	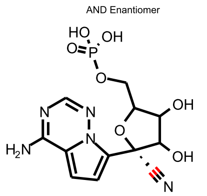 [*]C#[*]	0.461	10 out of 19
ECFP_12	-521596699	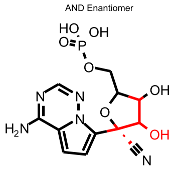 [*]C1[*][*]C([*])([*]<br>)C1O	0.445	3 out of 5

Top Features for negative contribution

Fingerprint	Bit/Smiles	Feature Structure	Score	Carcinogen in training set
ECFP_12	-1687549011	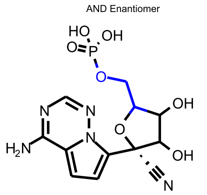 [*]OCC([*])[*]	-0.661	0 out of 3
ECFP_12	2024329577	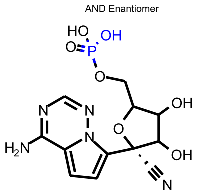 [*]P(=[*])([*])O	-0.661	0 out of 3

ECFP_12	-1734834311	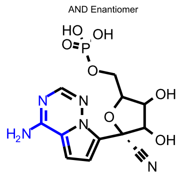 [*]:n:[c](N):[c](:[*]):[*]	-0.56	1 out of 8

Flavonoid-1

TOPKAT_Rat_Female_FDA_Single_vs_Multiple

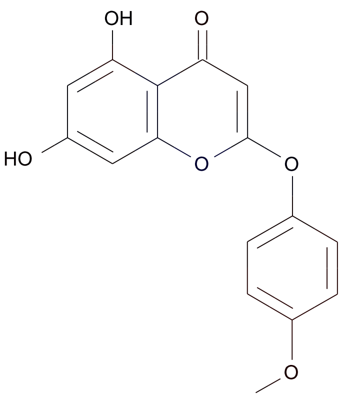

$C_{16}H_{12}O_6$
Molecular Weight: 300.26287
ALogP: 3.129
Rotatable Bonds: 3
Acceptors: 6
Donors: 2

Model Prediction

Prediction: Multiple-Carcinogen
Probability: 0.53
Enrichment: 1.42
Bayesian Score: 1.25
Mahalanobis Distance: 13.2
Mahalanobis Distance p-value: 0.000158

Prediction: Positive if the Bayesian score is above the estimated best cutoff value from minimizing the false positive and false negative rate.
Probability: The esimated probability that the sample is in the positive category. This assumes that the Bayesian score follows a normal distribution and is different from the prediction using a cutoff.
Enrichment: An estimate of enrichment, that is, the increased likelihood (versus random) of this sample being in the category.
Bayesian Score: The standard Laplacian-modified Bayesian score.
Mahalanobis Distance: The Mahalanobis distance (MD) is the distance to the center of the training data. The larger the MD, the less trustworthy the prediction.
Mahalanobis Distance p-value: The p-value gives the fraction of training data with an MD greater than or equal to the one for the given sample, assuming normally distributed data. The smaller the p-value, the less trustworthy the prediciton. For highly non-normal X properties (e.g., fingerprints), the MD p-value is wildly inaccurate.

Structural Similar Compounds

Name	Cytembena	Phenolphthalein	Omeprazole
Structure	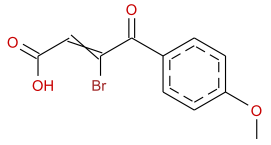	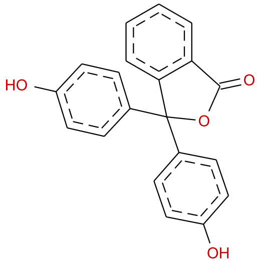	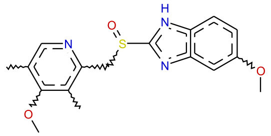
Actual Endpoint	Multiple-Carcinogen	Single-Carcinogen	Multiple-Carcinogen
Predicted Endpoint	Multiple-Carcinogen	Single-Carcinogen	Multiple-Carcinogen
Distance	0.592	0.600	0.653
Reference	US FDA (Centre for Drug Eval.& Res./Off. Testing & Res.) Sept. 1997	US FDA (Centre for Drug Eval.& Res./Off. Testing & Res.) Sept. 1997	US FDA (Centre for Drug Eval.& Res./Off. Testing & Res.) Sept. 1997

Model Applicability

Unknown features are fingerprint features in the query molecule, but not found or appearing too infrequently in the training set.

1. All properties and OPS components are within expected ranges.

Feature Contribution

Top features for positive contribution				
**Fingerprint**	**Bit/Smiles**	**Feature Structure**	**Score**	**Multiple-Carcinogen in training set**
SCFP_4	611156666	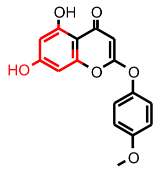 [*][c]1:[*]:[c]([*]):[cH]:[c](O):[cH]:1	0.627	5 out of 7

SCFP_4	1237755852	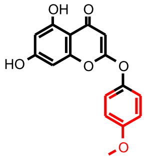 CO[c]1:[cH]:[cH]:[*]:[cH]:[cH]:1	0.295	5 out of 11
SCFP_4	-1971196727	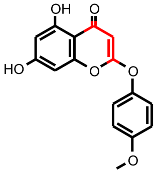 [*]C(=CC(=[*])[*])[*]	0.295	5 out of 11

Top Features for negative contribution

Fingerprint	Bit/Smiles	Feature Structure	Score	Multiple-Carcinogen in training set
SCFP_4	130348166	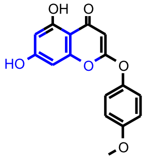 [*]O[c]1:[cH]:[c](O):[cH]:[*]:[c]:1[*]	-0.489	0 out of 2
SCFP_4	2019093677	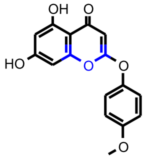 [*]C(=[*])O[c](:[*]):[*]	-0.274	0 out of 1
SCFP_4	616189553	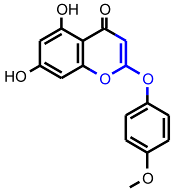 [*]OC(=C[*])O[*]	-0.274	0 out of 1

Flavonoid-1

TOPKAT_Rat_Male_FDA_None_vs_Carcinogen

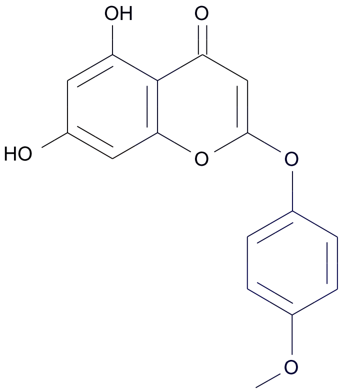

$C_{16}H_{12}O_6$

Molecular Weight: 300.26287

ALogP: 3.129

Rotatable Bonds: 3

Acceptors: 6

Donors: 2

Structural Similar Compounds

Name	Cytembena	Niclosamide	Mebendazole
Structure	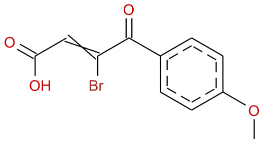	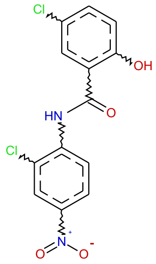	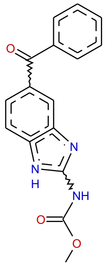
Actual Endpoint	Carcinogen	Non-Carcinogen	Non-Carcinogen
Predicted Endpoint	Carcinogen	Non-Carcinogen	Non-Carcinogen
Distance	0.613	0.620	0.630
Reference	US FDA (Centre for Drug Eval.& Res./Off. Testing & Res.) Sept. 1997	US FDA (Centre for Drug Eval.& Res./Off. Testing & Res.) Sept. 1997	US FDA (Centre for Drug Eval.& Res./Off. Testing & Res.) Sept. 1997

Model Prediction

Prediction: Non-Carcinogen

Probability: 0.282

Enrichment: 0.845

Bayesian Score: -2.87

Mahalanobis Distance: 11.7

Mahalanobis Distance p-value: 0.0514

Prediction: Positive if the Bayesian score is above the estimated best cutoff value from minimizing the false positive and false negative rate.
Probability: The esimated probability that the sample is in the positive category. This assumes that the Bayesian score follows a normal distribution and is different from the prediction using a cutoff.
Enrichment: An estimate of enrichment, that is, the increased likelihood (versus random) of this sample being in the category.
Bayesian Score: The standard Laplacian-modified Bayesian score.
Mahalanobis Distance: The Mahalanobis distance (MD) is the distance to the center of the training data. The larger the MD, the less trustworthy the prediction.
Mahalanobis Distance p-value: The p-value gives the fraction of training data with an MD greater than or equal to the one for the given sample, assuming normally distributed data. The smaller the p-value, the less trustworthy the prediciton. For highly non-normal X properties (e.g., fingerprints), the MD p-value is wildly inaccurate.

Model Applicability

Unknown features are fingerprint features in the query molecule, but not found or appearing too infrequently in the training set.

1. All properties and OPS components are within expected ranges.

Feature Contribution

Top features for positive contribution				
**Fingerprint**	**Bit/Smiles**	**Feature Structure**	**Score**	**Carcinogen in training set**
SCFP_6	-1971196727	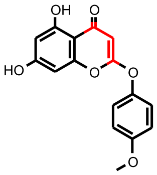 [*]C(=CC(=[*])[*])[*]	0.361	17 out of 36

SCFP_6	1157879834	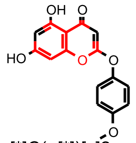<br>[*]C1=[*]C(=[*])[c]2:[c]([*]):[*]:[c]([*]):[cH]:[c]:2O1	0.198	1 out of 2
SCFP_6	611156666	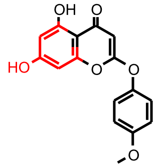<br>[*][c]1:[*]:[c]([*]):[cH]:[c](O):[cH]:1	0.186	6 out of 15
**Top Features for negative contribution**				
**Fingerprint**	**Bit/Smiles**	**Feature Structure**	**Score**	**Carcinogen in training set**
SCFP_6	-609499983	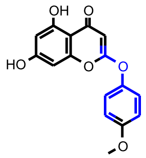<br>[*]C(=[*])O[c]1:[cH]:[cH]:[*]:[cH]:[cH]:1	-0.496	0 out of 2
SCFP_6	1287669168	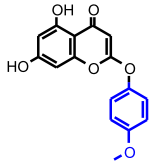<br>[*][c]1:[cH]:[cH]:[c](OC):[cH]:[cH]:1	-0.38	1 out of 6
SCFP_6	1742928053	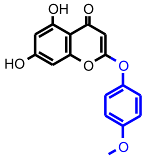<br>[*]O[c]1:[cH]:[cH]:[c](OC):[cH]:[cH]:1	-0.278	0 out of 1

Flavonoid-2

TOPKAT_Rat_Male_FDA_None_vs_Carcinogen

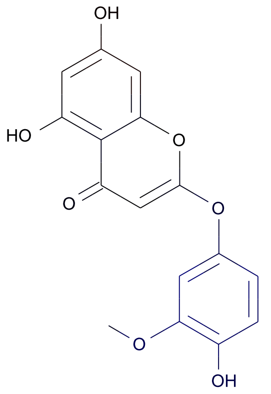

$C_{16}H_{12}O_7$

Molecular Weight: 316.26227

ALogP: 2.887

Rotatable Bonds: 3

Acceptors: 7

Donors: 3

Model Prediction

Prediction: Non-Carcinogen

Probability: 0.29

Enrichment: 0.868

Bayesian Score: -2.53

Mahalanobis Distance: 12

Mahalanobis Distance p-value: 0.0321

Prediction: Positive if the Bayesian score is above the estimated best cutoff value from minimizing the false positive and false negative rate.
Probability: The esimated probability that the sample is in the positive category. This assumes that the Bayesian score follows a normal distribution and is different from the prediction using a cutoff.
Enrichment: An estimate of enrichment, that is, the increased likelihood (versus random) of this sample being in the category.
Bayesian Score: The standard Laplacian-modified Bayesian score.
Mahalanobis Distance: The Mahalanobis distance (MD) is the distance to the center of the training data. The larger the MD, the less trustworthy the prediction.
Mahalanobis Distance p-value: The p-value gives the fraction of training data with an MD greater than or equal to the one for the given sample, assuming normally distributed data. The smaller the p-value, the less trustworthy the prediciton. For highly non-normal X properties (e.g., fingerprints), the MD p-value is wildly inaccurate.

Structural Similar Compounds

Name	Clorazepate	Olsalazine	Nedocromil
Structure	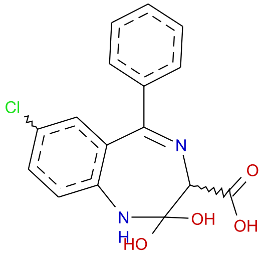	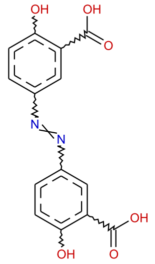	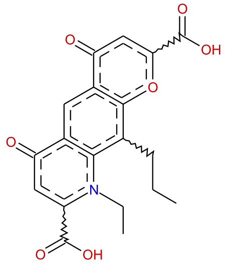
Actual Endpoint	Non-Carcinogen	Non-Carcinogen	Non-Carcinogen
Predicted Endpoint	Non-Carcinogen	Carcinogen	Non-Carcinogen
Distance	0.639	0.656	0.658
Reference	US FDA (Centre for Drug Eval.& Res./Off. Testing & Res.) Sept. 1997	US FDA (Centre for Drug Eval.& Res./Off. Testing & Res.) Sept. 1997	US FDA (Centre for Drug Eval.& Res./Off. Testing & Res.) Sept. 1997

Model Applicability

Unknown features are fingerprint features in the query molecule, but not found or appearing too infrequently in the training set.

1. All properties and OPS components are within expected ranges.

Feature Contribution

Top features for positive contribution				
**Fingerprint**	**Bit/Smiles**	**Feature Structure**	**Score**	**Carcinogen in training set**
SCFP_6	-1971196727	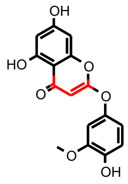 [*]C(=CC(=[*])[*])[*]	0.361	17 out of 36

SCFP_6	1157879834	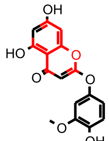 [*]C1=[*]C(=[*])[c]2:[c]([*]):[*]:[c]([*]):[cH]:[c]:2O1	0.198	1 out of 2
SCFP_6	611156666	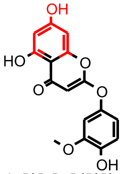 [*][c]1:[*]:[c]([*]):[cH]:[c](O):[cH]:1	0.186	6 out of 15
**Top Features for negative contribution**				
**Fingerprint**	**Bit/Smiles**	**Feature Structure**	**Score**	**Carcinogen in training set**
SCFP_6	2116304939	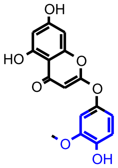 [*]O[c]1:[cH]:[*]:[cH]:[cH]:[c]:1O	-0.825	0 out of 4
SCFP_6	-609499983	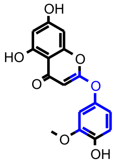 [*]C(=[*])O[c]1:[cH]:[cH]:[*]:[cH]:[cH]:1	-0.496	0 out of 2
SCFP_6	1570454387	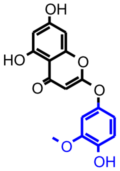 [*][c]1:[cH]:[cH]:[c](O):[c](OC):[cH]:1	-0.278	0 out of 1

remdesivir

TOPKAT_Rat_Male_FDA_None_vs_Carcinogen

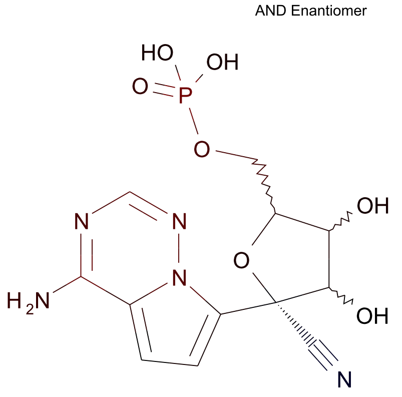

$C_{12}H_{14}N_5O_7P$
Molecular Weight: 371.24262
ALogP: -1.502
Rotatable Bonds: 4
Acceptors: 11
Donors: 5

Model Prediction

Prediction: Carcinogen
Probability: 0.481
Enrichment: 1.44
Bayesian Score: 3.82
Mahalanobis Distance: 14.1
Mahalanobis Distance p-value: 3.32e-005

Prediction: Positive if the Bayesian score is above the estimated best cutoff value from minimizing the false positive and false negative rate.
Probability: The esimated probability that the sample is in the positive category. This assumes that the Bayesian score follows a normal distribution and is different from the prediction using a cutoff.
Enrichment: An estimate of enrichment, that is, the increased likelihood (versus random) of this sample being in the category.
Bayesian Score: The standard Laplacian-modified Bayesian score.
Mahalanobis Distance: The Mahalanobis distance (MD) is the distance to the center of the training data. The larger the MD, the less trustworthy the prediction.
Mahalanobis Distance p-value: The p-value gives the fraction of training data with an MD greater than or equal to the one for the given sample, assuming normally distributed data. The smaller the p-value, the less trustworthy the prediciton. For highly non-normal X properties (e.g., fingerprints), the MD p-value is wildly inaccurate.

Structural Similar Compounds

Name	Streptozocin	Famotidine	Tetracycline
Structure	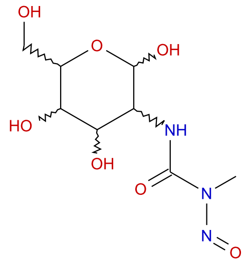	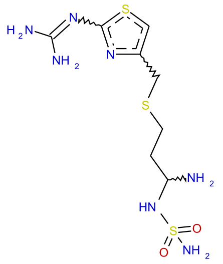	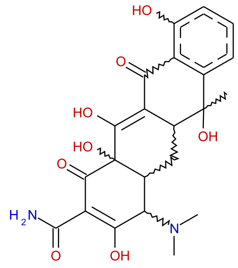
Actual Endpoint	Carcinogen	Non-Carcinogen	Non-Carcinogen
Predicted Endpoint	Carcinogen	Non-Carcinogen	Non-Carcinogen
Distance	0.789	0.850	0.856
Reference	US FDA (Centre for Drug Eval.& Res./Off. Testing & Res.) Sept. 1997	US FDA (Centre for Drug Eval.& Res./Off. Testing & Res.) Sept. 1997	US FDA (Centre for Drug Eval.& Res./Off. Testing & Res.) Sept. 1997

Model Applicability

Unknown features are fingerprint features in the query molecule, but not found or appearing too infrequently in the training set.

1. All properties and OPS components are within expected ranges.

Feature Contribution

Top features for positive contribution

Fingerprint	Bit/Smiles	Feature Structure	Score	Carcinogen in training set
SCFP_6	-1029620989	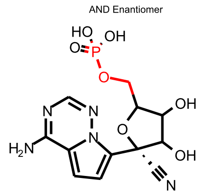 [*]COP(=[*])([*])[*]	0.712	3 out of 3

SCFP_6	1245795878	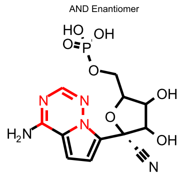<br>[*][c]1:[*]:n(:[*]):n:[cH]:n:1	0.603	2 out of 2
SCFP_6	149212520	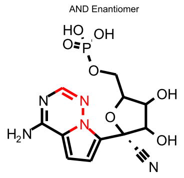<br>[*]:[cH]:n:n(:[*]):[*]]	0.543	9 out of 15

Top Features for negative contribution

Fingerprint	Bit/Smiles	Feature Structure	Score	Carcinogen in training set
SCFP_6	-1019297400	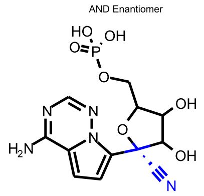<br>[*]C([*])([*])C#N	-0.674	0 out of 3
SCFP_6	194135988	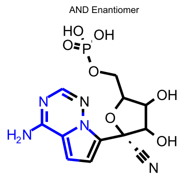<br>N[c]1:n:[cH]:[*]:n2:[*]:[*]:[cH]:[c]:1:2	-0.278	0 out of 1
SCFP_6	-424515134	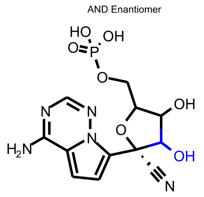<br>[*]C([*])O	-0.157	30 out of 110

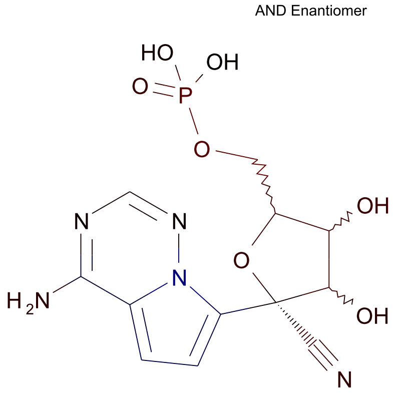

$C_{12}H_{14}N_5O_7P$
Molecular Weight: 371.24262
ALogP: -1.502
Rotatable Bonds: 4
Acceptors: 11
Donors: 5

Model Prediction

Prediction: Multiple-Carcinogen
Probability: 0.556
Enrichment: 1.34
Bayesian Score: 3.52
Mahalanobis Distance: 14
Mahalanobis Distance p-value: 8.72e-005

Prediction: Positive if the Bayesian score is above the estimated best cutoff value from minimizing the false positive and false negative rate.
Probability: The esimated probability that the sample is in the positive category. This assumes that the Bayesian score follows a normal distribution and is different from the prediction using a cutoff.
Enrichment: An estimate of enrichment, that is, the increased likelihood (versus random) of this sample being in the category.
Bayesian Score: The standard Laplacian-modified Bayesian score.
Mahalanobis Distance: The Mahalanobis distance (MD) is the distance to the center of the training data. The larger the MD, the less trustworthy the prediction.
Mahalanobis Distance p-value: The p-value gives the fraction of training data with an MD greater than or equal to the one for the given sample, assuming normally distributed data. The smaller the p-value, the less trustworthy the prediciton. For highly non-normal X properties (e.g., fingerprints), the MD p-value is wildly inaccurate.

Structural Similar Compounds

Name	Streptozocin	Minocycline	Ribavirin
Structure	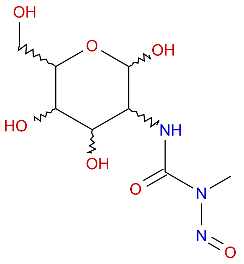	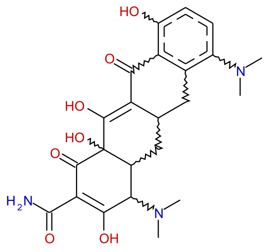	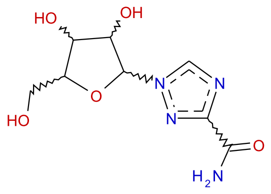
Actual Endpoint	Multiple-Carcinogen	Single-Carcinogen	Multiple-Carcinogen
Predicted Endpoint	Multiple-Carcinogen	Single-Carcinogen	Multiple-Carcinogen
Distance	0.817	0.908	0.929
Reference	US FDA (Centre for Drug Eval.& Res./Off. Testing & Res.) Sept. 1997	US FDA (Centre for Drug Eval.& Res./Off. Testing & Res.) Sept. 1997	US FDA (Centre for Drug Eval.& Res./Off. Testing & Res.) Sept. 1997

Model Applicability

Unknown features are fingerprint features in the query molecule, but not found or appearing too infrequently in the training set.

1. Num_H_Acceptors out of range. Value: 11. Training min, max, mean, SD: 0, 9, 3.8906, 2.196.

Feature Contribution

Top features for positive contribution

Fingerprint	Bit/Smiles	Feature Structure	Score	Multiple-Carcinogen in training set
SCFP_8	-1029620989	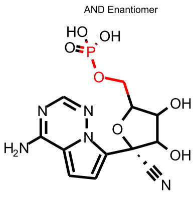 [*]COP(=[*])([*])[*]	0.649	3 out of 3

SCFP_8	2	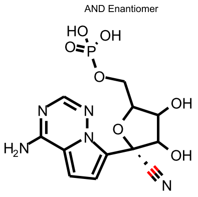<br>[*]C#[*]	0.584	6 out of 8
SCFP_8	-1486266146	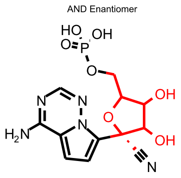<br>[*]CC1OC([*])([*])C(O)C1O	0.553	2 out of 2

Top Features for negative contribution

Fingerprint	Bit/Smiles	Feature Structure	Score	Multiple-Carcinogen in training set
SCFP_8	-1381862798	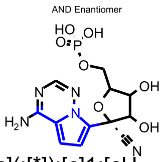<br>[*][c](:[*]):[c]1:[cH]:[cH]:[c]([*]):n:1: [*]	-0.572	1 out of 7
SCFP_8	1245795878	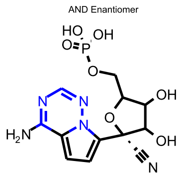<br>[*][c]1:[*]:n(:[*]):n :[cH]:n:1	-0.546	0 out of 2
SCFP_8	-1375522316	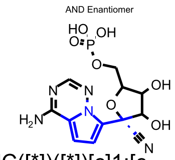<br>[*]C([*])([*])[c]1:[c H]:[cH]:[c](:[*]):n: 1:[*]	-0.546	0 out of 2

remdesivir

TOPKAT_Skin_Irritancy_Mild_vs_Moderate_Severe

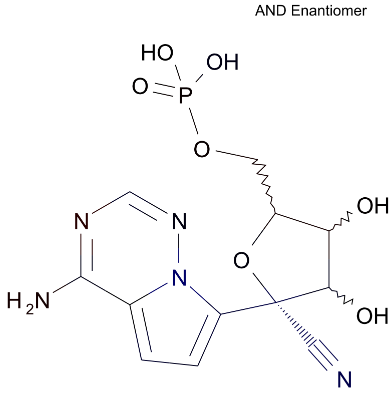

$C_{12}H_{14}N_5O_7P$
Molecular Weight: 371.24262
ALogP: -1.502
Rotatable Bonds: 4
Acceptors: 11
Donors: 5

Model Prediction

Prediction: Mild
Probability: 0.0911
Enrichment: 0.247
Bayesian Score: -8.73
Mahalanobis Distance: 13.5
Mahalanobis Distance p-value: 1.21e-009

Prediction: Positive if the Bayesian score is above the estimated best cutoff value from minimizing the false positive and false negative rate.
Probability: The esimated probability that the sample is in the positive category. This assumes that the Bayesian score follows a normal distribution and is different from the prediction using a cutoff.
Enrichment: An estimate of enrichment, that is, the increased likelihood (versus random) of this sample being in the category.
Bayesian Score: The standard Laplacian-modified Bayesian score.
Mahalanobis Distance: The Mahalanobis distance (MD) is the distance to the center of the training data. The larger the MD, the less trustworthy the prediction.
Mahalanobis Distance p-value: The p-value gives the fraction of training data with an MD greater than or equal to the one for the given sample, assuming normally distributed data. The smaller the p-value, the less trustworthy the prediciton. For highly non-normal X properties (e.g., fingerprints), the MD p-value is wildly inaccurate.

Structural Similar Compounds

Name	1,3,6-Naphthalenetrisulfonic acid, 7-amino-	2,7-Anthracenedisulfonic acid, 9,10-dihydro-4,5-diamino-9,10-dioxo- 1-hydroxy-, disodium salt	1,5-Naphthalenedisulfonic acid, 2-amino-
Structure	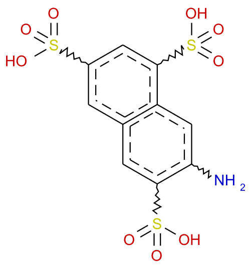	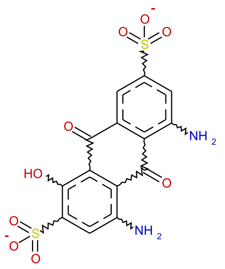	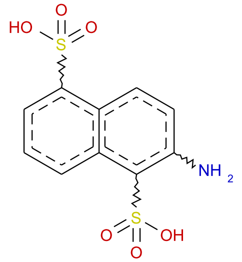
Actual Endpoint	Mild	Mild	Mild
Predicted Endpoint	Mild	Mild	Mild
Distance	0.759	1.033	1.137
Reference	85JCAE "Prehled Prumyslove Toxikologie; Organicke Latky," Marhold, J., Prague , Czechoslovakia, Avicenum, 1986 Volume(issue)/page/year: -,1058,1986	28ZPAK "Sbornik Vysledku Toxixologickeho Vysetreni Latek A Pripravku," Marhold d, J.V., Institut Pro Vychovu Vedoucicn Pracovniku Chemickeho Prumyclu Praha, Cz echoslovakia, 1972 Volume(issue)/page/year: -,239,1	85JCAE "Prehled Prumyslove Toxikologie; Organicke Latky," Marhold, J., Prague , Czechoslovakia, Avicenum, 1986 Volume(issue)/page/year: -,1058,1986

Model Applicability

Unknown features are fingerprint features in the query molecule, but not found or appearing too infrequently in the training set.

1. All properties and OPS components are within expected ranges.
2. Unknown FCFP_2 feature: 472180098: [*]OP(=O)(O)O
3. Unknown FCFP_2 feature: -332197802: [*][c]1:[*]:[*]:[c](:[*]):n:1:n:[*]

Feature Contribution

Top features for positive contribution				
Fingerprint	Bit/Smiles	Feature Structure	Score	Moderate_Severe in training set

FCFP_12	-1151884458	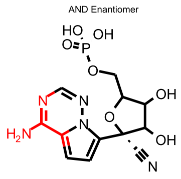 [*]:n:[c](N):[c](:[*]):[*]	0.385	1 out of 1
FCFP_12	76292238	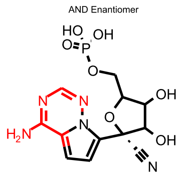 [*]:[c]1:[*]:n:[cH]:n:[c]:1N	0.385	1 out of 1
FCFP_12	-124685461	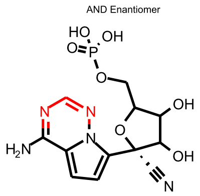 [*]:n:[cH]:n:[*]	0.206	2 out of 4

Top Features for negative contribution

Fingerprint	Bit/Smiles	Feature Structure	Score	Moderate_Severe in training set
FCFP_12	4427049	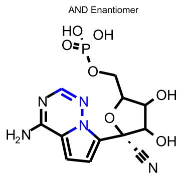 [*]:[cH]:n:n(:[*]):[*]	-0.893	0 out of 4
FCFP_12	-1277879912	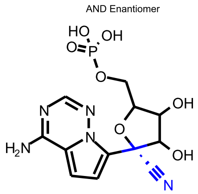 [*]C([*])([*])C#N	-0.548	5 out of 26

FCFP_12	-836603894	AND Enantiomer 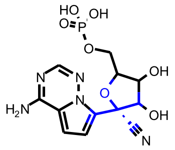 [*]C1[*][*]O[C@]1(C#[*])[c](:[*]):[*]	-0.543	0 out of 2

Flavonoid-1

TOPKAT_Skin_Irritancy_None_vs_Irritant

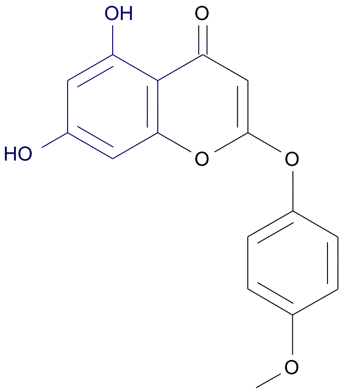

$C_{16}H_{12}O_6$

Molecular Weight: 300.26287

ALogP: 3.129

Rotatable Bonds: 3

Acceptors: 6

Donors: 2

Structural Similar Compounds

Name	Phenol, 4,4'-sulfonyldi-	Anthraquinone, 1,4-diamino-2-methoxy-	1-Amino-2-bromo-4-hydroxyanthraquinone
Structure	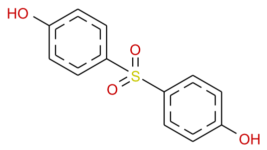	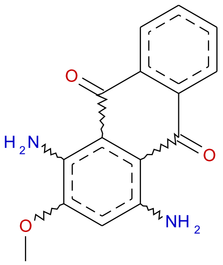	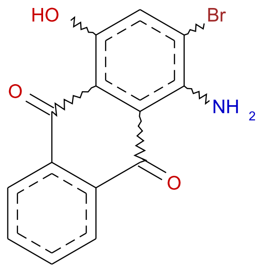
Actual Endpoint	Irritant	Irritant	Non-Irritant
Predicted Endpoint	Non-Irritant	Non-Irritant	Non-Irritant
Distance	0.682	0.692	0.697
Reference	BIOFX* BIOFAX Industrial Bio-Test Laboratories, Inc., Data Sheets. (1810 Fro ntage Rd., Northbrook, IL 60062) Volume(issue)/page/year: 601-05501,1974	NTIS** National Technical Information Service. (Springfield, VA 22161) Forme rly U.S. Clearinghouse for Scientific & Technical Information. Volume(issue)/pag e/year: AD-A172-758	28ZPAK -,83,72

Model Prediction

Prediction: Non-Irritant

Probability: 0.838

Enrichment: 0.91

Bayesian Score: -3.28

Mahalanobis Distance: 8.01

Mahalanobis Distance p-value: 0.88

Prediction: Positive if the Bayesian score is above the estimated best cutoff value from minimizing the false positive and false negative rate.
Probability: The esimated probability that the sample is in the positive category. This assumes that the Bayesian score follows a normal distribution and is different from the prediction using a cutoff.
Enrichment: An estimate of enrichment, that is, the increased likelihood (versus random) of this sample being in the category.
Bayesian Score: The standard Laplacian-modified Bayesian score.
Mahalanobis Distance: The Mahalanobis distance (MD) is the distance to the center of the training data. The larger the MD, the less trustworthy the prediction.
Mahalanobis Distance p-value: The p-value gives the fraction of training data with an MD greater than or equal to the one for the given sample, assuming normally distributed data. The smaller the p-value, the less trustworthy the prediciton. For highly non-normal X properties (e.g., fingerprints), the MD p-value is wildly inaccurate.

Model Applicability

Unknown features are fingerprint features in the query molecule, but not found or appearing too infrequently in the training set.

1. All properties and OPS components are within expected ranges.

Feature Contribution

Top features for positive contribution				
**Fingerprint**	**Bit/Smiles**	**Feature Structure**	**Score**	**Irritant in training set**

FCFP_12	451847724	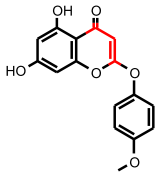 [*]C(=CC(=[*])[*])[*]	0.0737	270 out of 274
FCFP_12	356782498	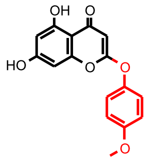 [*]O[c]1:[cH]:[cH]:[c](OC):[cH]:[cH]:1	0.0583	2 out of 2

Top Features for negative contribution

Fingerprint	Bit/Smiles	Feature Structure	Score	Irritant in training set
FCFP_12	946068634	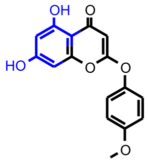 [*][c]1:[*]:[cH]:[c](O):[cH]:[c]:1O	-1.04	0 out of 2
FCFP_12	115228054	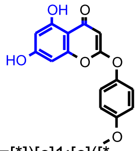 [*]C(=[*])[c]1:[c]([*]):[cH]:[c](O):[cH]: [c]:1O	-0.65	0 out of 1
FCFP_12	-1604301295	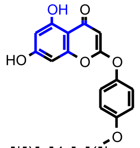 [*]C(=[*])[c]1:[c]([*]):[*]:[c]([*]):[cH] :[c]:1O	-0.18	22 out of 29

Flavonoid-2

TOPKAT_Skin_Irritancy_None_vs_Irritant

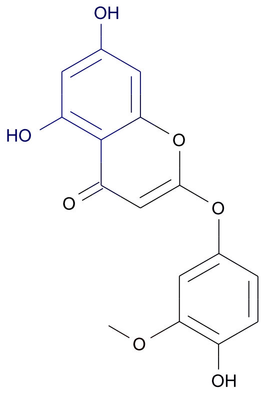

$C_{16}H_{12}O_7$
Molecular Weight: 316.26227
ALogP: 2.887
Rotatable Bonds: 3
Acceptors: 7
Donors: 3

Model Prediction

Prediction: Non-Irritant

Probability: 0.886

Enrichment: 0.962

Bayesian Score: -2.9

Mahalanobis Distance: 8.84

Mahalanobis Distance p-value: 0.506

Prediction: Positive if the Bayesian score is above the estimated best cutoff value from minimizing the false positive and false negative rate.
Probability: The esimated probability that the sample is in the positive category. This assumes that the Bayesian score follows a normal distribution and is different from the prediction using a cutoff.
Enrichment: An estimate of enrichment, that is, the increased likelihood (versus random) of this sample being in the category.
Bayesian Score: The standard Laplacian-modified Bayesian score.
Mahalanobis Distance: The Mahalanobis distance (MD) is the distance to the center of the training data. The larger the MD, the less trustworthy the prediction.
Mahalanobis Distance p-value: The p-value gives the fraction of training data with an MD greater than or equal to the one for the given sample, assuming normally distributed data. The smaller the p-value, the less trustworthy the prediciton. For highly non-normal X properties (e.g., fingerprints), the MD p-value is wildly inaccurate.

Structural Similar Compounds

Name	Anthraquinone, 1,2,4-trihydroxy-	8-Methylamino-4-hydroxy-2-naphthalene sulfonic acid	Benzenesulfonic acid, 2-anilino-5-nitro-
Structure	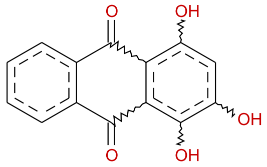	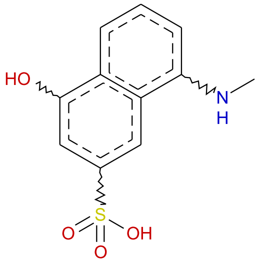	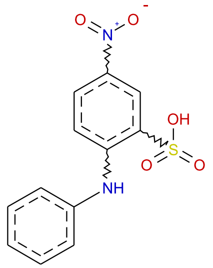
Actual Endpoint	Non-Irritant	Non-Irritant	Irritant
Predicted Endpoint	Non-Irritant	Non-Irritant	Non-Irritant
Distance	0.732	0.733	0.746
Reference	28ZPAK -,103,72	28ZPAK -,190,72	85JCAE "Prehled Prumyslove Toxikologie; Organicke Latky," Marhold, J., Prague , Czechoslovakia, Avicenum, 1986 Volume(issue)/page/year: -,1061,1986

Model Applicability

Unknown features are fingerprint features in the query molecule, but not found or appearing too infrequently in the training set.

1. All properties and OPS components are within expected ranges.

Feature Contribution

Top features for positive contribution				
Fingerprint	Bit/Smiles	Feature Structure	Score	Irritant in training set

FCFP_12	523826990	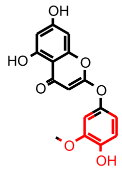 [*]O[c]1:[cH]:[*]:[cH]:[cH]:[c]:1O	0.0756	6 out of 6
FCFP_12	451847724	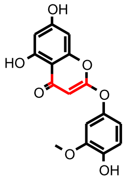 [*]C(=CC(=[*])[*])[*]	0.0737	270 out of 274
FCFP_12	301073077	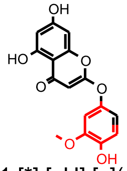 [*][c]1:[*]:[cH]:[c](O):[c](OC):[cH]:1	0.0734	5 out of 5
**Top Features for negative contribution**				
**Fingerprint**	**Bit/Smiles**	**Feature Structure**	**Score**	**Irritant in training set**
FCFP_12	946068634	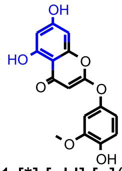 [*][c]1:[*]:[cH]:[c](O):[cH]:[c]:1O	-1.04	0 out of 2
FCFP_12	115228054	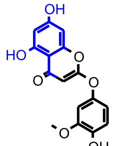 [*]C(=[*])[c]1:[c]([*]):[cH]:[c](O):[cH]:[c]:1O	-0.65	0 out of 1

FCFP_12	-1604301295	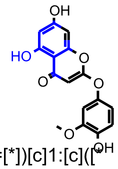 [*]C(=[*])[c]1:[c]([*]):[*]:[c]([*]):[cH]:[c]:1O	-0.18	22 out of 29

remdesivir

TOPKAT_Skin_Irritancy_None_vs_Irritant

AND Enantiomer

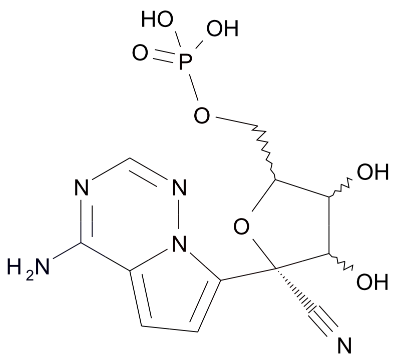

$C_{12}H_{14}N_5O_7P$

Molecular Weight: 371.24262

ALogP: -1.502

Rotatable Bonds: 4

Acceptors: 11

Donors: 5

Structural Similar Compounds

Name	1,3,6-Naphthalenetrisulfonic acid, 7-amino-	2,2'-Benzidine disulfonic acid	2,7-Anthracenedisulfonic acid, 9,10-dihydro-4,5-diamino-9,10-dioxo- 1-hydroxy-, disodium salt
Structure	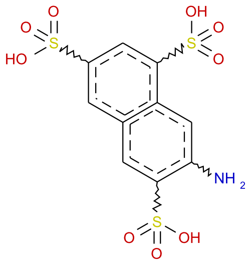	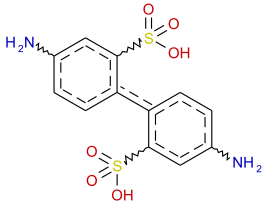	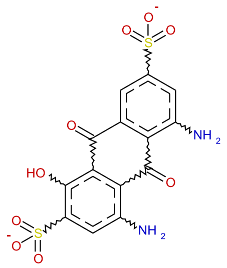
Actual Endpoint	Irritant	Non-Irritant	Irritant
Predicted Endpoint	Non-Irritant	Non-Irritant	Non-Irritant
Distance	0.755	0.896	1.025
Reference	85JCAE "Prehled Prumyslove Toxikologie; Organicke Latky," Marhold, J., Prague , Czechoslovakia, Avicenum, 1986 Volume(issue)/page/year: -,1058,1986	28ZPAK -,191,72	28ZPAK "Sbornik Vysledku Toxixologickeho Vysetreni Latek A Pripravku," Marhol d, J.V., Institut Pro Vychovu Vedoucicn Pracovniku Chemickeho Prumyclu Praha, Cz echoslovakia, 1972 Volume(issue)/page/year: -,239,1

Model Prediction

Prediction: Irritant

Probability: 0.976

Enrichment: 1.06

Bayesian Score: -0.492

Mahalanobis Distance: 13.2

Mahalanobis Distance p-value: 3.18e-008

Prediction: Positive if the Bayesian score is above the estimated best cutoff value from minimizing the false positive and false negative rate.
Probability: The esimated probability that the sample is in the positive category. This assumes that the Bayesian score follows a normal distribution and is different from the prediction using a cutoff.
Enrichment: An estimate of enrichment, that is, the increased likelihood (versus random) of this sample being in the category.
Bayesian Score: The standard Laplacian-modified Bayesian score.
Mahalanobis Distance: The Mahalanobis distance (MD) is the distance to the center of the training data. The larger the MD, the less trustworthy the prediction.
Mahalanobis Distance p-value: The p-value gives the fraction of training data with an MD greater than or equal to the one for the given sample, assuming normally distributed data. The smaller the p-value, the less trustworthy the prediciton. For highly non-normal X properties (e.g., fingerprints), the MD p-value is wildly inaccurate.

Model Applicability

Unknown features are fingerprint features in the query molecule, but not found or appearing too infrequently in the training set.

1. All properties and OPS components are within expected ranges.
2. Unknown FCFP_2 feature: 472180098: [*]OP(=O)(O)O
3. Unknown FCFP_2 feature: -332197802: [*][c]1:[*]:[*]:[c](:[*]):n:1:n:[*]

Feature Contribution

Top features for positive contribution				
**Fingerprint**	**Bit/Smiles**	**Feature Structure**	**Score**	**Irritant in training set**

FCFP_12	654335567	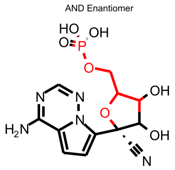 [*]C1[*][*]OC1COP(=[*])([*])[*]	0.0856	29 out of 29
FCFP_12	-1539132615	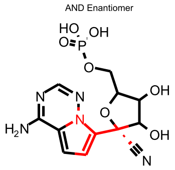 [*]C([*])([*])[c]1:[c H]:[*]:[*]:n:1:[*]	0.0795	9 out of 9
FCFP_12	-1280036918	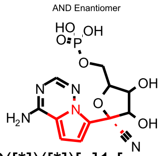 [*]C([*])([*])[c]1:[c H]:[cH]:[c](:[*]):n: 1:[*]	0.0772	7 out of 7

Top Features for negative contribution

**Fingerprint**	**Bit/Smiles**	**Feature Structure**	**Score**	**Irritant in training set**
FCFP_12	1069584379	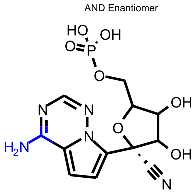 [*]:[c](:[*])N	-0.439	38 out of 65
FCFP_12	1618154665	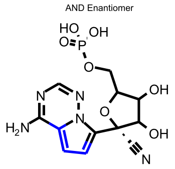 [*][c](:[*]):[cH]:[cH]:[*]	-0.0845	412 out of 490

FCFP_12	16	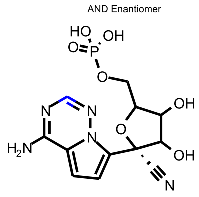 [*][c](:[*]):[*]	-0.0843	423 out of 503

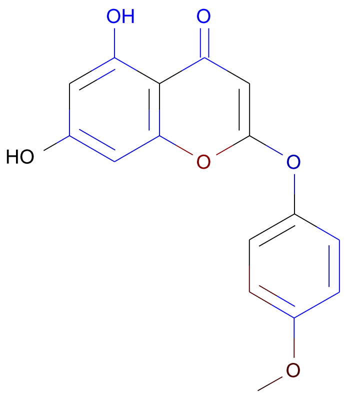

$C_{16}H_{12}O_6$

Molecular Weight: 300.26287

ALogP: 3.129

Rotatable Bonds: 3

Acceptors: 6

Donors: 2

Model Prediction

Prediction: 145

Unit: mg/kg_body_weight/day

Mahalanobis Distance: 10.7

Mahalanobis Distance p-value: 0.00377

Mahalanobis Distance: The Mahalanobis distance (MD) is a generalization of the Euclidean distance that accounts for correlations among the X properties. It is calculated as the distance to the center of the training data. The larger the MD, the less trustworthy the prediction.
Mahalanobis Distance p-value: The p-value gives the fraction of training data with an MD greater than or equal to the one for the given sample, assuming normally distributed data. The smaller the p-value, the less trustworthy the prediciton. For highly non-normal X properties (e.g., fingerprints), the MD p-value is wildly inaccurate.

Structural Similar Compounds

Name	C.I. pigment red 3	422	44
Structure	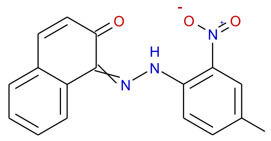	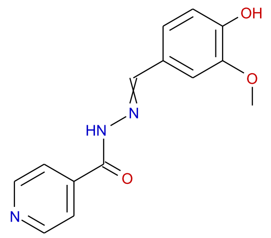	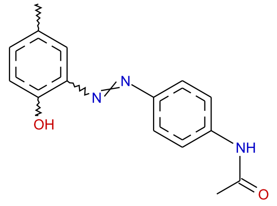
Actual Endpoint (-log C)	0.937339	3.99565	2.42163
Predicted Endpoint (-log C)	3.17837	3.22211	2.85113
Distance	0.615	0.632	0.641
Reference	CPDB	CPDB	CPDB

Model Applicability

Unknown features are fingerprint features in the query molecule, but not found or appearing too infrequently in the training set.

1. All properties and OPS components are within expected ranges.
2. Unknown ECFP_2 feature: 367973906: [*]OC(=C[*])O[*]

Feature Contribution

Top features for positive contribution

Fingerprint	Bit/Smiles	Feature Structure	Score
ECFP_6	683445015	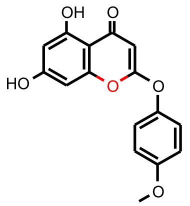 [*]O[*]	0.136

ECFP_6	-176455838	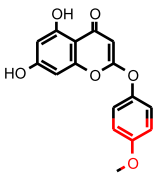 [*]O[c](:[cH]:[*]):[cH]:[*]	0.0818
ECFP_6	734603939	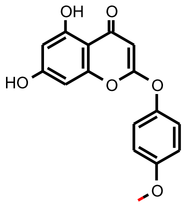 [*]C	0.0424
**Top Features for negative contribution**			
**Fingerprint**	**Bit/Smiles**	**Feature Structure**	**Score**
ECFP_6	2106656448	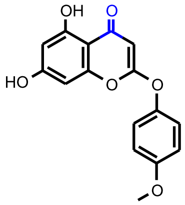 [*]C(=O)[*]	-0.275
ECFP_6	2019062761	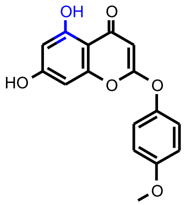 [*]:[c](:[*])O	-0.258
ECFP_6	1996767644	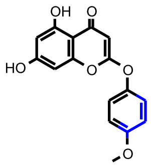 [*][c](:[*]):[cH]:[cH]:[*]	-0.251

Flavonoid-2 | TOPKAT_Carcinogenic_Potency_TD50_Mouse

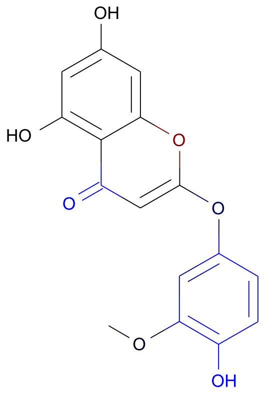

$C_{16}H_{12}O_7$

Molecular Weight: 316.26227

ALogP: 2.887

Rotatable Bonds: 3

Acceptors: 7

Donors: 3

Model Prediction

Prediction: 113

Unit: mg/kg_body_weight/day

Mahalanobis Distance: 10.1

Mahalanobis Distance p-value: 0.0254

Mahalanobis Distance: The Mahalanobis distance (MD) is a generalization of the Euclidean distance that accounts for correlations among the X properties. It is calculated as the distance to the center of the training data. The larger the MD, the less trustworthy the prediction.
Mahalanobis Distance p-value: The p-value gives the fraction of training data with an MD greater than or equal to the one for the given sample, assuming normally distributed data. The smaller the p-value, the less trustworthy the prediciton. For highly non-normal X properties (e.g., fingerprints), the MD p-value is wildly inaccurate.

Structural Similar Compounds

Name	542	Ochratoxin A	422
Structure	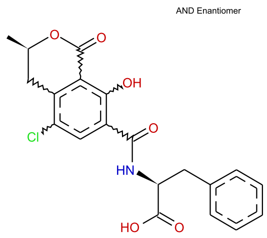	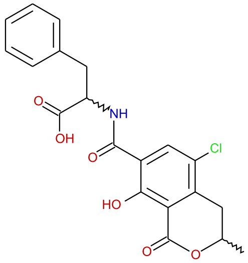	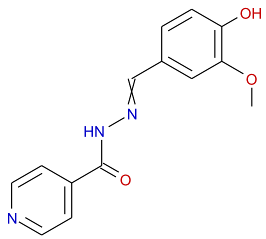
Actual Endpoint (-log C)	4.79932	4.79932	3.99565
Predicted Endpoint (-log C)	3.6353	3.6353	3.22211
Distance	0.621	0.621	0.684
Reference	CPDB	CPDB	CPDB

Model Applicability

Unknown features are fingerprint features in the query molecule, but not found or appearing too infrequently in the training set.

1. OPS PC13 out of range. Value: -3.1563. Training min, max, SD, explained variance: -3.068, 3.6909, 1.329, 0.0220.
2. Unknown ECFP_2 feature: 367973906: [*]OC(=C[*])O[*]

Feature Contribution

Top features for positive contribution

Fingerprint	Bit/Smiles	Feature Structure	Score
ECFP_6	683445015	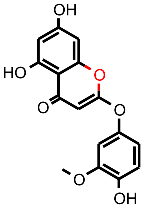 [*]O[*]	0.136

ECFP_6	-176455838	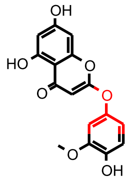<br>[*]O[c](:[cH]:[*]):[c<br>H]:[*]	0.0818
ECFP_6	734603939	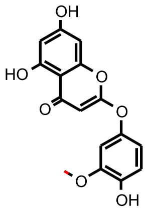<br>[*]C	0.0424
**Top Features for negative contribution**			
**Fingerprint**	**Bit/Smiles**	**Feature Structure**	**Score**
ECFP_6	2106656448	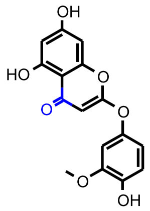<br>[*]C(=O)[*]	-0.275
ECFP_6	2019062761	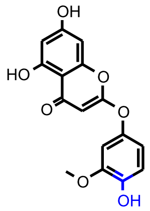<br>[*]:[c](:[*])O	-0.258
ECFP_6	1996767644	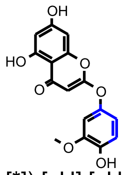<br>[*][c](:[*]):[cH]:[cH<br>]:[*]	-0.251

remdesivir

TOPKAT_Carcinogenic_Potency_TD50_Mouse

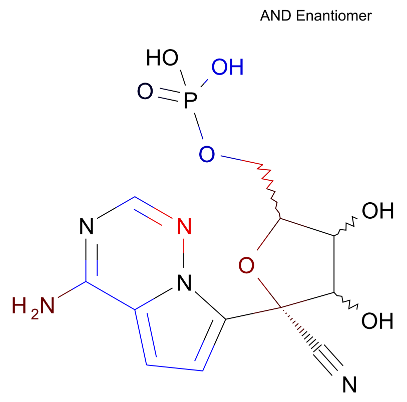

$C_{12}H_{14}N_5O_7P$

Molecular Weight: 371.24262

ALogP: -1.502

Rotatable Bonds: 4

Acceptors: 11

Donors: 5

Structural Similar Compounds

Name	377	(N-6)-(Methylnitroso)adenosine	338
Structure	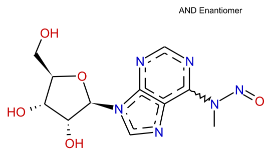	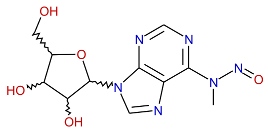	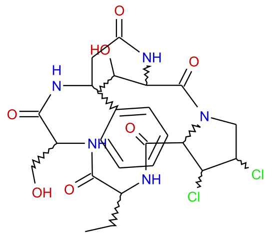
Actual Endpoint (-log C)	4.22928	4.22928	4.39533
Predicted Endpoint (-log C)	5.36013	5.36013	4.31268
Distance	0.852	0.852	0.919
Reference	CPDB	CPDB	CPDB

Model Prediction

Prediction: 9.25

Unit: mg/kg_body_weight/day

Mahalanobis Distance: 14

Mahalanobis Distance p-value: 2.59e-010

Mahalanobis Distance: The Mahalanobis distance (MD) is a generalization of the Euclidean distance that accounts for correlations among the X properties. It is calculated as the distance to the center of the training data. The larger the MD, the less trustworthy the prediction.
Mahalanobis Distance p-value: The p-value gives the fraction of training data with an MD greater than or equal to the one for the given sample, assuming normally distributed data. The smaller the p-value, the less trustworthy the prediciton. For highly non-normal X properties (e.g., fingerprints), the MD p-value is wildly inaccurate.

Model Applicability

Unknown features are fingerprint features in the query molecule, but not found or appearing too infrequently in the training set.

1. All properties and OPS components are within expected ranges.
2. Unknown ECFP_2 feature: 1126642748: [*]OP(=O)(O)O
3. Unknown ECFP_2 feature: 2024329577: [*]P(=[*])([*])O
4. Unknown ECFP_2 feature: -194719409: [*]C1[*][*]C([*])([*])O1
5. Unknown ECFP_2 feature: 1258791451: [*]C1[*][*]O[C@]1(C#[*])[c](:[*]):[*]
6. Unknown ECFP_2 feature: -264833661: [*]C([*])([*])C#N
7. Unknown ECFP_2 feature: -1507082173: [*][c]1:[*]:[*]:[c](:[*]):n:1:n:[*]
8. Unknown ECFP_2 feature: -676555381: [*]:[cH]:n:n(:[*]):[*]
9. Unknown ECFP_2 feature: -66263742: [*]C([*])([*])[c]1:[cH]:[*]:[*]:n:1:[*]

Feature Contribution

Top features for positive contribution			
**Fingerprint**	**Bit/Smiles**	**Feature Structure**	**Score**

ECFP_6	655739385	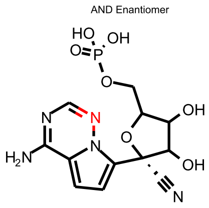 [*]:n:[*]	0.229
ECFP_6	1572579716	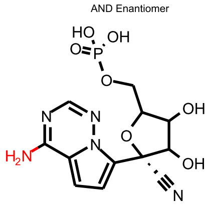 [*]N	0.225
ECFP_6	1559650422	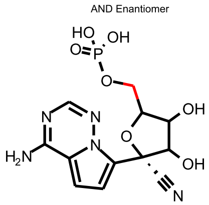 [*]C[*]	0.203
**Top Features for negative contribution**			
**Fingerprint**	**Bit/Smiles**	**Feature Structure**	**Score**
ECFP_6	1996767644	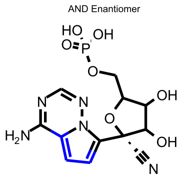 [*][c](:[*]):[cH]:[cH]:[*]	-0.251
ECFP_6	642810091	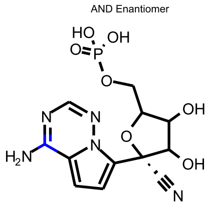 [*][c](:[*]):[*]	-0.247

ECFP_6	-182236392	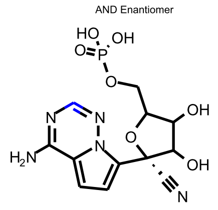 [*]:[cH]:[*]	-0.232

Flavonoid-1

TOPKAT_Carcinogenic_Potency_TD50_Rat

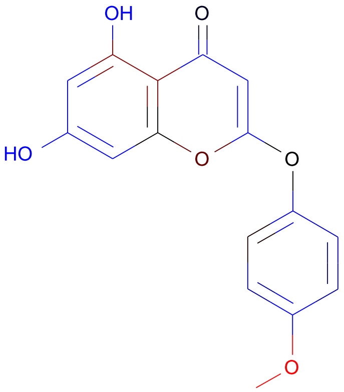

$C_{16}H_{12}O_6$

Molecular Weight: 300.26287

ALogP: 3.129

Rotatable Bonds: 3

Acceptors: 6

Donors: 2

Model Prediction

Prediction: 22.1

Unit: mg/kg_body_weight/day

Mahalanobis Distance: 12.2

Mahalanobis Distance p-value: 0.000206

Mahalanobis Distance: The Mahalanobis distance (MD) is a generalization of the Euclidean distance that accounts for correlations among the X properties. It is calculated as the distance to the center of the training data. The larger the MD, the less trustworthy the prediction.

Mahalanobis Distance p-value: The p-value gives the fraction of training data with an MD greater than or equal to the one for the given sample, assuming normally distributed data. The smaller the p-value, the less trustworthy the prediciton. For highly non-normal X properties (e.g., fingerprints), the MD p-value is wildly inaccurate.

Structural Similar Compounds

Name	44	C.I. pigment red 3	Chrysazin
Structure	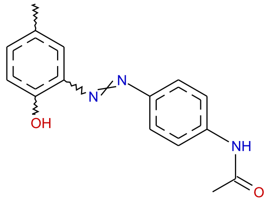	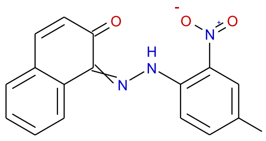	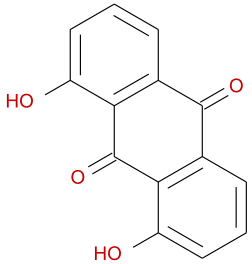
Actual Endpoint (-log C)	2.85045	2.41938	2.99143
Predicted Endpoint (-log C)	2.7768	4.26375	3.29868
Distance	0.591	0.597	0.627
Reference	CPDB	CPDB	CPDB

Model Applicability

Unknown features are fingerprint features in the query molecule, but not found or appearing too infrequently in the training set.

1. All properties and OPS components are within expected ranges.
2. Unknown FCFP_2 feature: -2115241127: [*]OC(=C[*])O[*]

Feature Contribution

Top features for positive contribution			
**Fingerprint**	**Bit/Smiles**	**Feature Structure**	**Score**
FCFP_6	136627117	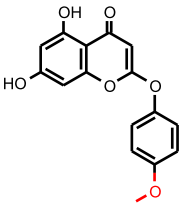 [*]OC	0.69

FCFP_6	1	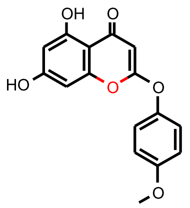<br>[*]O[*]	0.234
FCFP_6	203677720	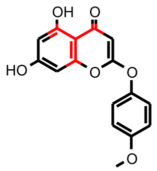<br>[*]C(=[*])[c](:[c]([*]):[*]):[c]([*]):[*]	0.137

Top Features for negative contribution

Fingerprint	Bit/Smiles	Feature Structure	Score
FCFP_6	451847724	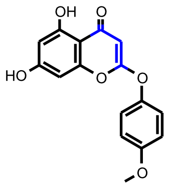<br>[*]C(=CC(=[*])[*])[*]	-0.436
FCFP_6	7	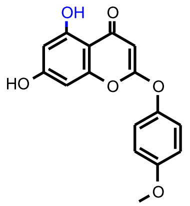<br>[*]O	-0.372
FCFP_6	16	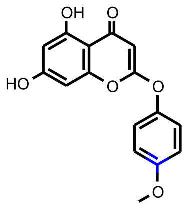<br>[*][c](:[*]):[*]	-0.354

Flavonoid-2

TOPKAT_Carcinogenic_Potency_TD50_Rat

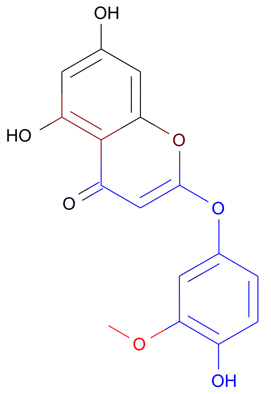

$C_{16}H_{12}O_7$

Molecular Weight: 316.26227

ALogP: 2.887

Rotatable Bonds: 3

Acceptors: 7

Donors: 3

Model Prediction

Prediction: 27.1

Unit: mg/kg_body_weight/day

Mahalanobis Distance: 10.5

Mahalanobis Distance p-value: 0.0688

Mahalanobis Distance: The Mahalanobis distance (MD) is a generalization of the Euclidean distance that accounts for correlations among the X properties. It is calculated as the distance to the center of the training data. The larger the MD, the less trustworthy the prediction.
Mahalanobis Distance p-value: The p-value gives the fraction of training data with an MD greater than or equal to the one for the given sample, assuming normally distributed data. The smaller the p-value, the less trustworthy the prediciton. For highly non-normal X properties (e.g., fingerprints), the MD p-value is wildly inaccurate.

Structural Similar Compounds

Name	542	Ochratoxin A	Quercetin
Structure	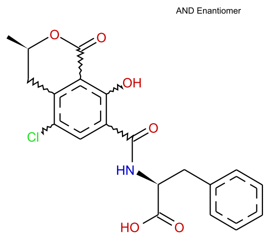	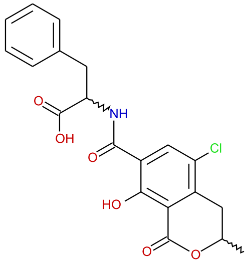	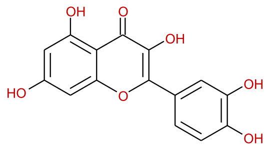
Actual Endpoint (-log C)	6.59334	6.47264	4.47602
Predicted Endpoint (-log C)	5.06501	5.06501	3.79194
Distance	0.616	0.616	0.627
Reference	CPDB	CPDB	CPDB

Model Applicability

Unknown features are fingerprint features in the query molecule, but not found or appearing too infrequently in the training set.

1. All properties and OPS components are within expected ranges.
2. Unknown FCFP_2 feature: -2115241127: [*]OC(=C[*])O[*]

Feature Contribution

Top features for positive contribution			
**Fingerprint**	**Bit/Smiles**	**Feature Structure**	**Score**
FCFP_6	136627117	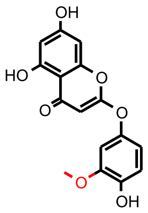 [*]OC	0.69

FCFP_6	1	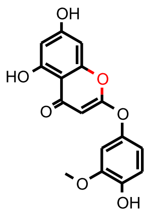 [*]O[*]	0.234
FCFP_6	203677720	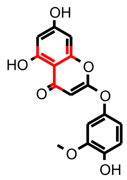 [*]C(=[*])[c](:[c]([*]):[*]):[c]([*]):[*]	0.137
**Top Features for negative contribution**			
**Fingerprint**	**Bit/Smiles**	**Feature Structure**	**Score**
FCFP_6	451847724	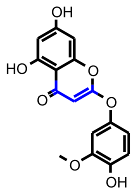 [*]C(=CC(=[*])[*])[*]	-0.436
FCFP_6	7	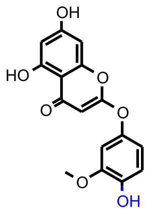 [*]O	-0.372
FCFP_6	16	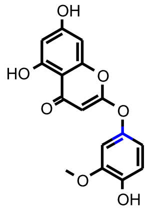 [*][c](:[*]):[*]	-0.354

remdesivir

TOPKAT_Carcinogenic_Potency_TD50_Rat

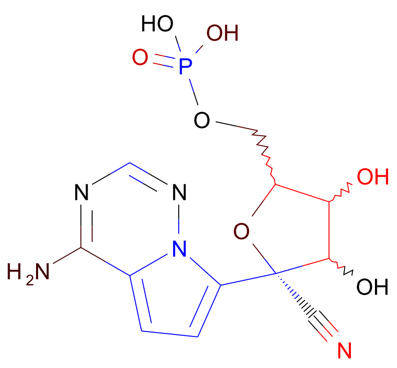

$C_{12}H_{14}N_5O_7P$
Molecular Weight: 371.24262
ALogP: -1.502
Rotatable Bonds: 4
Acceptors: 11
Donors: 5

Structural Similar Compounds

Name	b-Thioguanine deoxyriboside	Hexamethylmelamine	604
Structure	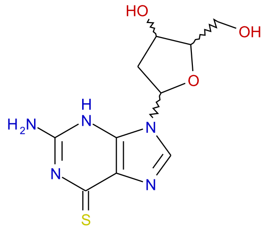	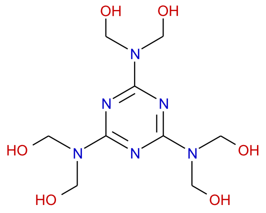	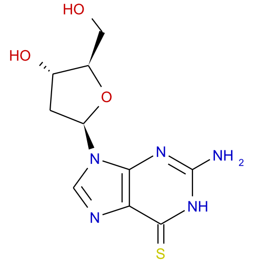
Actual Endpoint (-log C)	5.13004	4.47751	5.13004
Predicted Endpoint (-log C)	4.82552	3.76275	4.96687
Distance	0.805	0.832	0.835
Reference	CPDB	CPDB	CPDB

Model Prediction

Prediction: 1.01
Unit: mg/kg_body_weight/day
Mahalanobis Distance: 16.2
Mahalanobis Distance p-value: 4.38e-015

Mahalanobis Distance: The Mahalanobis distance (MD) is a generalization of the Euclidean distance that accounts for correlations among the X properties. It is calculated as the distance to the center of the training data. The larger the MD, the less trustworthy the prediction.
Mahalanobis Distance p-value: The p-value gives the fraction of training data with an MD greater than or equal to the one for the given sample, assuming normally distributed data. The smaller the p-value, the less trustworthy the prediciton. For highly non-normal X properties (e.g., fingerprints), the MD p-value is wildly inaccurate.

Model Applicability

Unknown features are fingerprint features in the query molecule, but not found or appearing too infrequently in the training set.

1. All properties and OPS components are within expected ranges.
2. Unknown FCFP_2 feature: 472180098: [*]OP(=O)(O)O
3. Unknown FCFP_2 feature: -836603894: [*]C1[*][*]O[C@]1(C#[*])[c](:[*]):[*]

Feature Contribution

Top features for positive contribution

Fingerprint	Bit/Smiles	Feature Structure	Score
FCFP_6	-1043250487	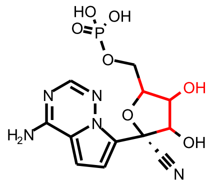 AND Enantiomer [*]C1[*][*]C([*])C1O	1.15

FCFP_6	9	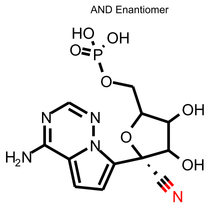 [*]#N	0.385
FCFP_6	1	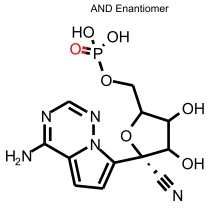 [*]O[*]	0.234
**Top Features for negative contribution**			
**Fingerprint**	**Bit/Smiles**	**Feature Structure**	**Score**
FCFP_6	-1280036918	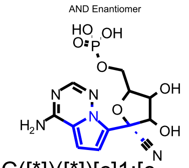 [*]C([*])([*])[c]1:[cH]:[cH]:[c](:[*]):n:1:[*]	-0.363
FCFP_6	16	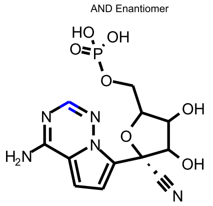 [*][c](:[*]):[*]	-0.354
FCFP_6	17	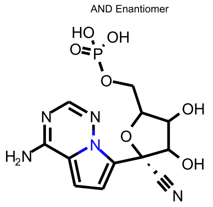 [*]:n(:[*]):[*]	-0.149

Flavonoid-1

TOPKAT_Chronic_LOAEL

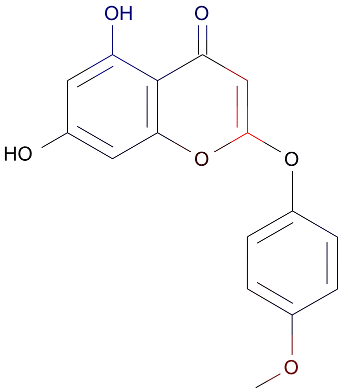

$C_{16}H_{12}O_6$
Molecular Weight: 300.26287
ALogP: 3.129
Rotatable Bonds: 3
Acceptors: 6
Donors: 2

Model Prediction

Prediction: 0.0222
Unit: g/kg_body_weight
Mahalanobis Distance: 27.3
Mahalanobis Distance p-value: 8.22e-020

Mahalanobis Distance: The Mahalanobis distance (MD) is a generalization of the Euclidean distance that accounts for correlations among the X properties. It is calculated as the distance to the center of the training data. The larger the MD, the less trustworthy the prediction.
Mahalanobis Distance p-value: The p-value gives the fraction of training data with an MD greater than or equal to the one for the given sample, assuming normally distributed data. The smaller the p-value, the less trustworthy the prediciton. For highly non-normal X properties (e.g., fingerprints), the MD p-value is wildly inaccurate.

Structural Similar Compounds

Name	HC BLUE 1	ZEARALENONE	3;3'-DIMETHOXYBENZIDINE .2HCL
Structure	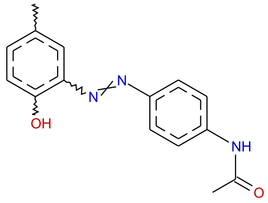	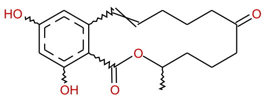	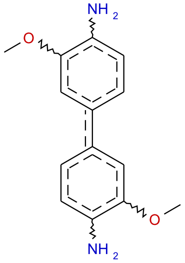
Actual Endpoint (-log C)	3.0323	5.40602	4.79463
Predicted Endpoint (-log C)	2.7171	3.57081	3.61371
Distance	0.562	0.585	0.603
Reference	NTP REPORT # 222	NTP REPORT # 235	NTP REPORT # 372

Model Applicability

Unknown features are fingerprint features in the query molecule, but not found or appearing too infrequently in the training set.

1. All properties and OPS components are within expected ranges.
2. Unknown FCFP_2 feature: -2115241127: [*]OC(=C[*])O[*]
3. Unknown ECFP_6 feature: 367973906: [*]OC(=C[*])O[*]
4. Unknown ECFP_6 feature: 464808839: [*]C(=CC(=[*])[*])[*]
5. Unknown ECFP_6 feature: -560785749: [*]C(=[*])O[c](:[*]):[*]
6. Unknown ECFP_6 feature: 1299558496: [*]=CC(=O)[c](:[*]):[*]
7. Unknown ECFP_6 feature: -570915357: [*]O[c](:[cH]:[*]):[c]([*]):[*]
8. Unknown ECFP_6 feature: -813997308: [*]C(=[*])[c](:[c]([*]):[*]):[c]([*]):[*]
9. Unknown ECFP_6 feature: -177786161: [*]:[cH]:[c](O):[cH]:[*]
10. Unknown ECFP_6 feature: 1305253718: [*]C(=[*])O[c](:[*]):[*]
11. Unknown ECFP_6 feature: 1307307440: [*]:[c](:[*])OC
12. Unknown ECFP_6 feature: 2019062761: [*]:[c](:[*])O

Feature Contribution

Top features for positive contribution			
**Fingerprint**	**Bit/Smiles**	**Feature Structure**	**Score**

FCFP_6	451847724	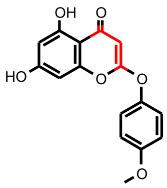<br>[*]C(=CC(=[*])[*])[*]	0.16
ECFP_6	-176455838	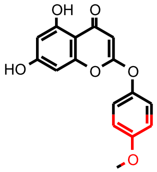<br>[*]O[c](:[cH]:[*]):[c<br>H]:[*]	0.106
ECFP_6	683445015	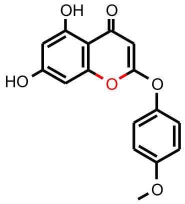<br>[*]O[*]	0.0734
**Top Features for negative contribution**			
**Fingerprint**	**Bit/Smiles**	**Feature Structure**	**Score**
ECFP_6	2106656448	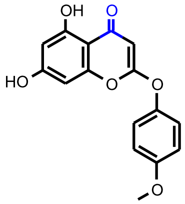<br>[*]C(=O)[*]	-0.11
FCFP_6	1	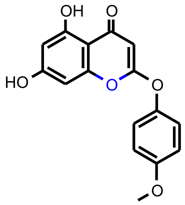<br>[*]O[*]	-0.102

FCFP_6	203677720	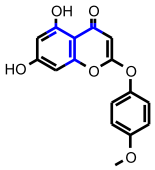 [*]C(=[*])[c](:[c]([*]):[*]):[c]([*]):[*]	-0.0713

Flavonoid-2

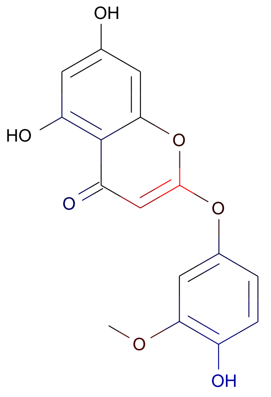

$C_{16}H_{12}O_7$

Molecular Weight: 316.26227

ALogP: 2.887

Rotatable Bonds: 3

Acceptors: 7

Donors: 3

Model Prediction

Prediction: 0.0362

Unit: g/kg_body_weight

Mahalanobis Distance: 27.9

Mahalanobis Distance p-value: 9.53e-021

Mahalanobis Distance: The Mahalanobis distance (MD) is a generalization of the Euclidean distance that accounts for correlations among the X properties. It is calculated as the distance to the center of the training data. The larger the MD, the less trustworthy the prediction.
Mahalanobis Distance p-value: The p-value gives the fraction of training data with an MD greater than or equal to the one for the given sample, assuming normally distributed data. The smaller the p-value, the less trustworthy the prediciton. For highly non-normal X properties (e.g., fingerprints), the MD p-value is wildly inaccurate.

Structural Similar Compounds

Name	QUERCETIN	OLSALAZINE.NA	CHLORSULFURON
Structure	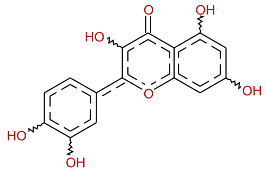	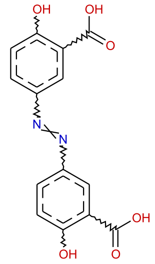	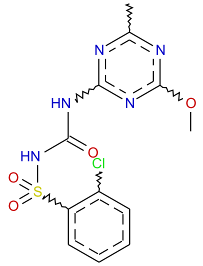
Actual Endpoint (-log C)	2.87829	3.17932	4.15566
Predicted Endpoint (-log C)	3.12498	2.89417	3.79771
Distance	0.596	0.604	0.623
Reference	NTP 409 79	NDA-19715	EPA COVER SHEET 0027;880301;(1)

Model Applicability

Unknown features are fingerprint features in the query molecule, but not found or appearing too infrequently in the training set.

1. All properties and OPS components are within expected ranges.
2. Unknown FCFP_2 feature: -2115241127: [*]OC(=C[*])O[*]
3. Unknown ECFP_6 feature: 2019062761: [*]:[c](:[*])O
4. Unknown ECFP_6 feature: -570915357: [*]O[c](:[cH]:[*]):[c]([*]):[*]
5. Unknown ECFP_6 feature: -813997308: [*]C(=[*])[c](:[c]([*]):[*]):[c]([*]):[*]
6. Unknown ECFP_6 feature: -177786161: [*]:[cH]:[c](O):[cH]:[*]
7. Unknown ECFP_6 feature: -560785749: [*]C(=[*])O[c](:[*]):[*]
8. Unknown ECFP_6 feature: 367973906: [*]OC(=C[*])O[*]
9. Unknown ECFP_6 feature: 1299558496: [*]=CC(=O)[c](:[*]):[*]
10. Unknown ECFP_6 feature: 464808839: [*]C(=CC(=[*])[*])[*]
11. Unknown ECFP_6 feature: 1305253718: [*]C(=[*])O[c](:[*]):[*]
12. Unknown ECFP_6 feature: 1307307440: [*]:[c](:[*])OC

Feature Contribution

Top features for positive contribution			
Fingerprint	Bit/Smiles	Feature Structure	Score

FCFP_6	451847724	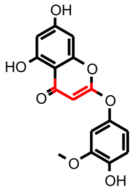 [*]C(=CC(=[*])[*])[*]	0.16
ECFP_6	-176455838	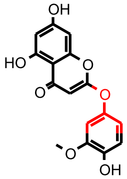 [*]O[c](:[cH]:[*]):[c H]:[*]	0.106
ECFP_6	683445015	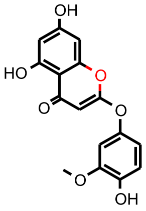 [*]O[*]	0.0734
**Top Features for negative contribution**			
**Fingerprint**	**Bit/Smiles**	**Feature Structure**	**Score**
ECFP_6	2106656448	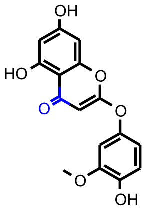 [*]C(=O)[*]	-0.11
FCFP_6	1	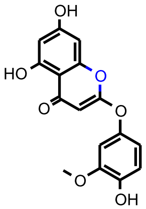 [*]O[*]	-0.102

FCFP_6	203677720	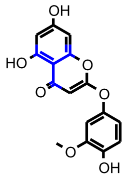 [*]C(=[*])[c](:[c]([*]):[*]):[c]([*]):[*]	-0.0713

remdesivir

TOPKAT_Chronic_LOAEL

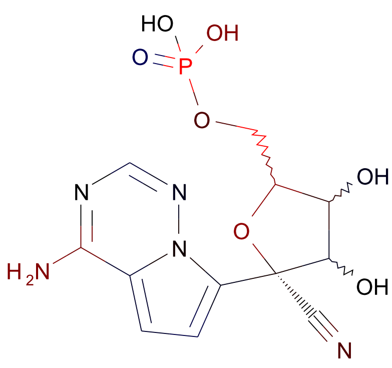

$C_{12}H_{14}N_5O_7P$

Molecular Weight: 371.24262

ALogP: -1.502

Rotatable Bonds: 4

Acceptors: 11

Donors: 5

Model Prediction

Prediction: 0.00379

Unit: g/kg_body_weight

Mahalanobis Distance: 47.7

Mahalanobis Distance p-value: 2.93e-054

Mahalanobis Distance: The Mahalanobis distance (MD) is a generalization of the Euclidean distance that accounts for correlations among the X properties. It is calculated as the distance to the center of the training data. The larger the MD, the less trustworthy the prediction.
Mahalanobis Distance p-value: The p-value gives the fraction of training data with an MD greater than or equal to the one for the given sample, assuming normally distributed data. The smaller the p-value, the less trustworthy the prediciton. For highly non-normal X properties (e.g., fingerprints), the MD p-value is wildly inaccurate.

Structural Similar Compounds

Name	TETRACYCLINE .HCL	4;4'-DIAMINO-2;2'-STILBENEDIS	OXYTETRACYCLINE .HCL
Structure	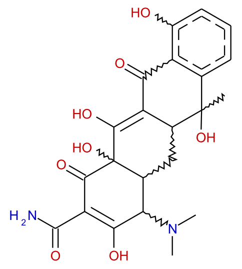	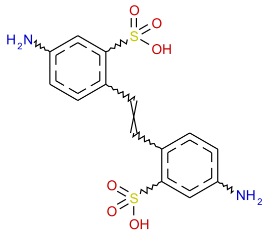	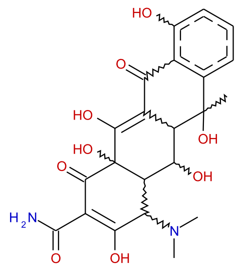
Actual Endpoint (-log C)	2.85193	2.47175	2.56626
Predicted Endpoint (-log C)	3.94748	3.53715	3.75581
Distance	0.746	0.746	0.802
Reference	NTP REPORT # 344	NTP 412 82	NTP REPORT # 315

Model Applicability

Unknown features are fingerprint features in the query molecule, but not found or appearing too infrequently in the training set.

1. All properties and OPS components are within expected ranges.
2. Unknown FCFP_2 feature: 472180098: [*]OP(=O)(O)O
3. Unknown FCFP_2 feature: -332197802: [*][c]1:[*]:[*]:[c](:[*]):n:1:n:[*]
4. Unknown ECFP_6 feature: -1114776580: [*]C#[*]
5. Unknown ECFP_6 feature: -1101847286: [*]#N
6. Unknown ECFP_6 feature: 672362763: [*]:n(:[*]):[*]
7. Unknown ECFP_6 feature: 1126642748: [*]OP(=O)(O)O
8. Unknown ECFP_6 feature: 2100964382: [*]P(=O)([*])[*]
9. Unknown ECFP_6 feature: 2024329577: [*]P(=[*])([*])O
10. Unknown ECFP_6 feature: -1250439909: [*]COP(=[*])([*])[*]
11. Unknown ECFP_6 feature: -1687549011: [*]OCC([*])[*]
12. Unknown ECFP_6 feature: -194719409: [*]C1[*][*]C([*])([*])O1
13. Unknown ECFP_6 feature: -553149446: [*]CC1O[*][*]C1[*]
14. Unknown ECFP_6 feature: 305695353: [*]C1[*][*]C([*])C1O
15. Unknown ECFP_6 feature: -521596699: [*]C1[*][*]C([*])([*])C1O
16. Unknown ECFP_6 feature: 1258791451: [*]C1[*][*]O[C@]1(C#[*])[c](:[*]):[*]
17. Unknown ECFP_6 feature: 2024749573: [*]C([*])O
18. Unknown ECFP_6 feature: -264833661: [*]C([*])([*])C#N
19. Unknown ECFP_6 feature: 1412053881: [*]C#N

20. Unknown ECFP_6 feature: -1507082173: [*][c]1:[*]:[*]:[c](:[*]):n:1:n:[*]
21. Unknown ECFP_6 feature: -676555381: [*]:[cH]:n:n(:[*]):[*]
22. Unknown ECFP_6 feature: -710237522: [*]:n:[cH]:n:[*]
23. Unknown ECFP_6 feature: -677309799: [*][c](:[*]):n:[cH]:[*]
24. Unknown ECFP_6 feature: -1734834311: [*]:n:[c](N):[c](:[*]):[*]
25. Unknown ECFP_6 feature: 1334415134: [*][c](:[*]):[c]1:[cH]:[*]:[*]:n:1:[*]
26. Unknown ECFP_6 feature: -66263742: [*]C([*])([*])[c]1:[cH]:[*]:[*]:n:1:[*]
27. Unknown ECFP_6 feature: -938530932: [*]:[c](:[*])N

Feature Contribution

Top features for positive contribution			
**Fingerprint**	**Bit/Smiles**	**Feature Structure**	**Score**
ECFP_6	-167460056	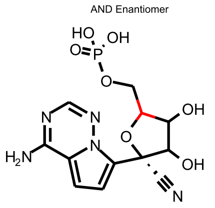<br>[*]C([*])[*]	0.136
FCFP_6	-1143715940	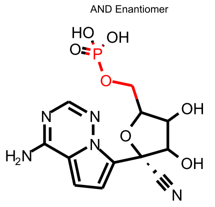<br>[*]COP(=[*])([*])[*]	0.13
ECFP_6	1559650422	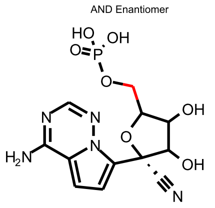<br>[*]C[*]	0.129

Top Features for negative contribution			
**Fingerprint**	**Bit/Smiles**	**Feature Structure**	**Score**

FCFP_6	1	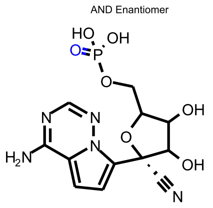 [*]O[*]	-0.102
ECFP_6	1996767644	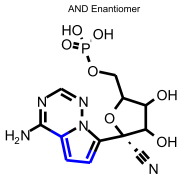 [*][c](:[*]):[cH]:[cH]:[*]	-0.0497
FCFP_6	16	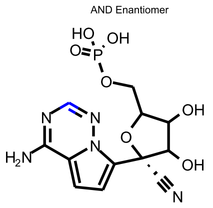 [*][c](:[*]):[*]	-0.0462

Flavonoid-1

TOPKAT_Rat_Maximum_Tolerated_Dose_Feed

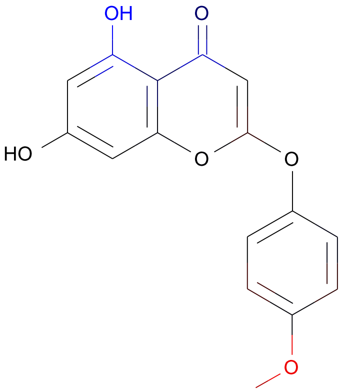

$C_{16}H_{12}O_6$

Molecular Weight: 300.26287

ALogP: 3.129

Rotatable Bonds: 3

Acceptors: 6

Donors: 2

Model Prediction

Prediction: 0.29

Unit: g/kg_body_weight

Mahalanobis Distance: 5.39

Mahalanobis Distance p-value: 0.879

Mahalanobis Distance: The Mahalanobis distance (MD) is a generalization of the Euclidean distance that accounts for correlations among the X properties. It is calculated as the distance to the center of the training data. The larger the MD, the less trustworthy the prediction.
Mahalanobis Distance p-value: The p-value gives the fraction of training data with an MD greater than or equal to the one for the given sample, assuming normally distributed data. The smaller the p-value, the less trustworthy the prediciton. For highly non-normal X properties (e.g., fingerprints), the MD p-value is wildly inaccurate.

Structural Similar Compounds

Name	DISPERSE YELLOW 3	BENZIDINE,3,3'-DIMETHOXY-	PHENOLPHTHALEIN
Structure	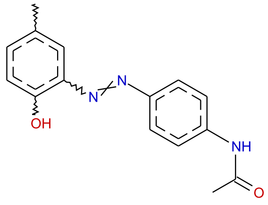	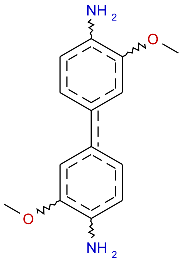	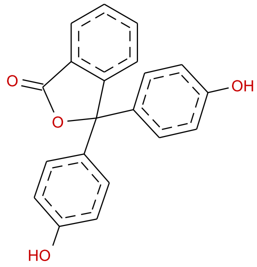
Actual Endpoint (-log C)	2.77703	4.06569	2.20184
Predicted Endpoint (-log C)	2.80195	3.57405	2.8857
Distance	0.461	0.545	0.546
Reference	NCI/NTP TR-222	NCI/NTP Report 10, Nov. 1987	NCI/NTP TR-465

Model Applicability

Unknown features are fingerprint features in the query molecule, but not found or appearing too infrequently in the training set.

1. All properties and OPS components are within expected ranges.
2. Unknown FCFP_2 feature: -2115241127: [*]OC(=C[*])O[*]

Feature Contribution

Top features for positive contribution			
Fingerprint	Bit/Smiles	Feature Structure	Score
FCFP_2	136627117	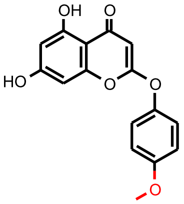 [*]OC	0.173

FCFP_2	1036089772	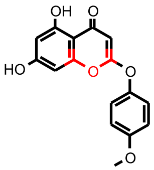 [*]C(=[*])O[c](:[*]):[*]	0.0749
FCFP_2	332760439	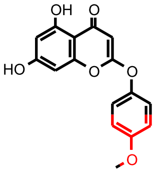 [*]O[c](:[cH]:[*]):[cH]:[*]	0.0611
**Top Features for negative contribution**			
**Fingerprint**	**Bit/Smiles**	**Feature Structure**	**Score**
FCFP_2	7	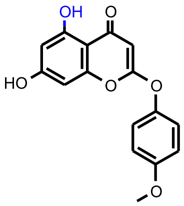 [*]O	-0.214
FCFP_2	-549108873	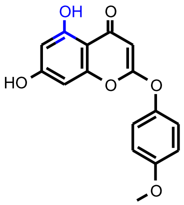 [*]:[c](:[*])O	-0.127
FCFP_2	1872154524	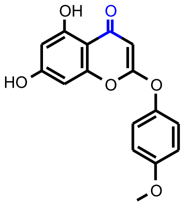 [*]C(=O)[*]	-0.105

Flavonoid-2

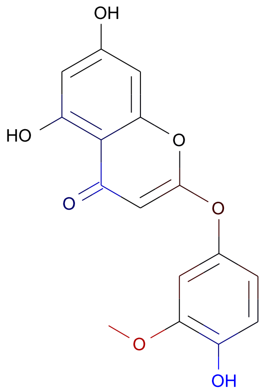

$C_{16}H_{12}O_7$
Molecular Weight: 316.26227
ALogP: 2.887
Rotatable Bonds: 3
Acceptors: 7
Donors: 3

Model Prediction

Prediction: 0.443
Unit: g/kg_body_weight
Mahalanobis Distance: 5.85
Mahalanobis Distance p-value: 0.709

Mahalanobis Distance: The Mahalanobis distance (MD) is a generalization of the Euclidean distance that accounts for correlations among the X properties. It is calculated as the distance to the center of the training data. The larger the MD, the less trustworthy the prediction.
Mahalanobis Distance p-value: The p-value gives the fraction of training data with an MD greater than or equal to the one for the given sample, assuming normally distributed data. The smaller the p-value, the less trustworthy the prediciton. For highly non-normal X properties (e.g., fingerprints), the MD p-value is wildly inaccurate.

Structural Similar Compounds

Name	QUERCETIN	PROPYL GALLATE	DISPERSE YELLOW 3
Structure	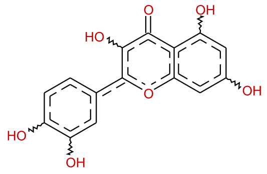	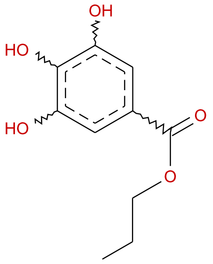	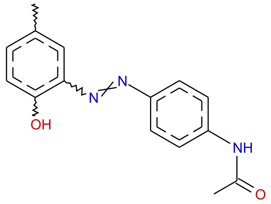
Actual Endpoint (-log C)	2.2016	2.59435	2.77703
Predicted Endpoint (-log C)	2.27782	2.18569	2.80195
Distance	0.548	0.621	0.635
Reference	NCI/NTP TR-409	NCI/NTP TR-240	NCI/NTP TR-222

Model Applicability

Unknown features are fingerprint features in the query molecule, but not found or appearing too infrequently in the training set.

1. All properties and OPS components are within expected ranges.
2. Unknown FCFP_2 feature: -2115241127: [*]OC(=C[*])O[*]

Feature Contribution

Top features for positive contribution			
**Fingerprint**	**Bit/Smiles**	**Feature Structure**	**Score**
FCFP_2	136627117	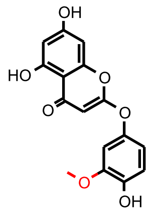 [*]OC	0.173

FCFP_2	1036089772	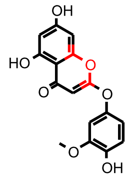[*]C(=[*])O[c](:[*]):[*]	0.0749
FCFP_2	332760439	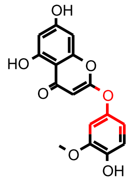[*]O[c](:[cH]:[*]):[cH]:[*]	0.0611
**Top Features for negative contribution**			
**Fingerprint**	**Bit/Smiles**	**Feature Structure**	**Score**
FCFP_2	7	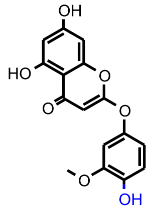[*]O	-0.214
FCFP_2	-549108873	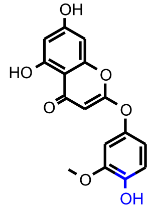[*]:[c](:[*])O	-0.127
FCFP_2	1872154524	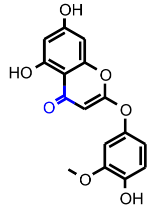[*]C(=O)[*]	-0.105

remdesivir

TOPKAT_Rat_Maximum_Tolerated_Dose_Feed

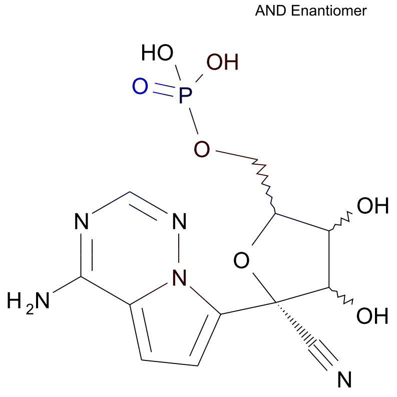

$C_{12}H_{14}N_5O_7P$

Molecular Weight: 371.24262

ALogP: -1.502

Rotatable Bonds: 4

Acceptors: 11

Donors: 5

Structural Similar Compounds

Name	4,4'-DIAMINO-2,2'-STILBENEDISULFONIC ACID.2NaSALT	OXYTETRACYCLINE	50%1,4,5,8-TETRAAMINOANTHRAQUIONONE + DERIVATIVES
Structure	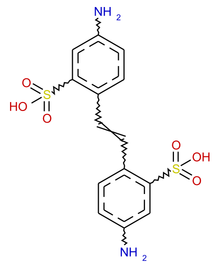	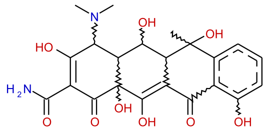	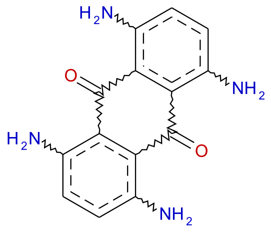
Actual Endpoint (-log C)	2.50759	2.36214	3.0764
Predicted Endpoint (-log C)	3.26068	2.77834	3.08142
Distance	0.743	0.818	0.989
Reference	NCI/NTP TR-412	NCI/NTP TR-315	NCI/NTP TR-299

Model Prediction

Prediction: 0.235

Unit: g/kg_body_weight

Mahalanobis Distance: 9.52

Mahalanobis Distance p-value: 0.000247

Mahalanobis Distance: The Mahalanobis distance (MD) is a generalization of the Euclidean distance that accounts for correlations among the X properties. It is calculated as the distance to the center of the training data. The larger the MD, the less trustworthy the prediction.
Mahalanobis Distance p-value: The p-value gives the fraction of training data with an MD greater than or equal to the one for the given sample, assuming normally distributed data. The smaller the p-value, the less trustworthy the prediciton. For highly non-normal X properties (e.g., fingerprints), the MD p-value is wildly inaccurate.

Model Applicability

Unknown features are fingerprint features in the query molecule, but not found or appearing too infrequently in the training set.

1. Molecular_PolarSurfaceArea out of range. Value: 206.26. Training min, max, mean, SD: 0, 201.84, 63.052, 40.7.
2. Unknown FCFP_2 feature: 472180098: [*]OP(=O)(O)O
3. Unknown FCFP_2 feature: -836603894: [*]C1[*][*]O[C@]1(C#[*])[c](:[*]):[*]
4. Unknown FCFP_2 feature: -1277879912: [*]C([*])([*])C#N
5. Unknown FCFP_2 feature: -332197802: [*][c]1:[*]:[*]:[c](:[*]):n:1:n:[*]
6. Unknown FCFP_2 feature: -124685461: [*]:n:[cH]:n:[*]

Feature Contribution

Top features for positive contribution

Fingerprint	Bit/Smiles	Feature Structure	Score

FCFP_2	-1143715940	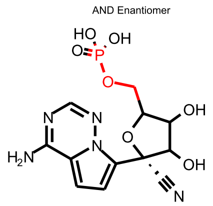<br>[*]COP(=[*])([*])[*]	0.095
FCFP_2	3	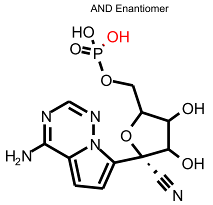<br>[*]O	0.0737
FCFP_2	17	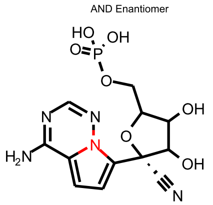<br>[*]:n(:[*]):[*]	0.0441
**Top Features for negative contribution**			
**Fingerprint**	**Bit/Smiles**	**Feature Structure**	**Score**
FCFP_2	1872154524	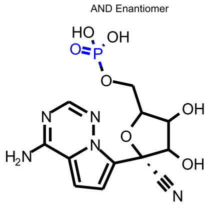<br>[*]C(=O)[*]	-0.105
FCFP_2	1	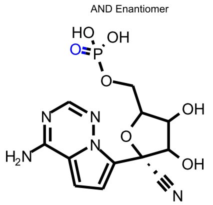<br>[*]O[*]	-0.0796

FCFP_2	16	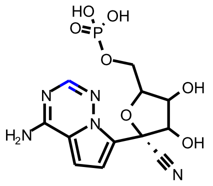 [*][c](:[*]):[*]	-0.0512

Flavonoid-1

TOPKAT_Rat_Maximum_Tolerated_Dose_Gavage

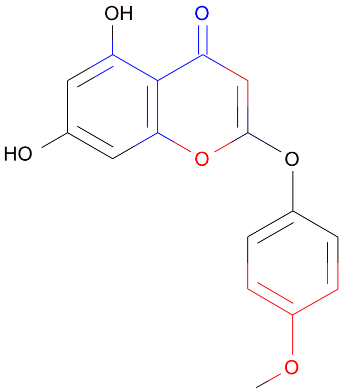

$C_{16}H_{12}O_6$

Molecular Weight: 300.26287

ALogP: 3.129

Rotatable Bonds: 3

Acceptors: 6

Donors: 2

Model Prediction

Prediction: 0.000241

Unit: g/kg_body_weight

Mahalanobis Distance: 8.74

Mahalanobis Distance p-value: 0.000264

Mahalanobis Distance: The Mahalanobis distance (MD) is a generalization of the Euclidean distance that accounts for correlations among the X properties. It is calculated as the distance to the center of the training data. The larger the MD, the less trustworthy the prediction.

Mahalanobis Distance p-value: The p-value gives the fraction of training data with an MD greater than or equal to the one for the given sample, assuming normally distributed data. The smaller the p-value, the less trustworthy the prediciton. For highly non-normal X properties (e.g., fingerprints), the MD p-value is wildly inaccurate.

Structural Similar Compounds

Name	OCHRATOXIN	SULFISOOXAZOLE	PENICILLIN VK
Structure	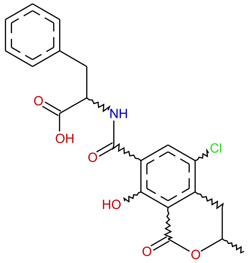	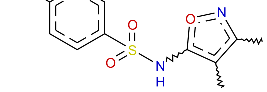	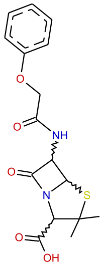
Actual Endpoint (-log C)	6.28396	2.82494	2.54455
Predicted Endpoint (-log C)	5.12358	3.0705	3.9702
Distance	0.752	0.765	0.821
Reference	NCI/NTP TR-358	NCI/NTP TR-138	NCI/NTP TR-336

Model Applicability

Unknown features are fingerprint features in the query molecule, but not found or appearing too infrequently in the training set.

1. OPS PC9 out of range. Value: 4.9977. Training min, max, SD, explained variance: -2.7086, 2.9267, 1.019, 0.0321.
2. Unknown FCFP_2 feature: -2115241127: [*]OC(=C[*])O[*]
3. Unknown FCFP_2 feature: -1549192822: [*]=CC(=O)[c](:[*]):[*]

Feature Contribution

Top features for positive contribution

Fingerprint	Bit/Smiles	Feature Structure	Score
FCFP_2	332760439	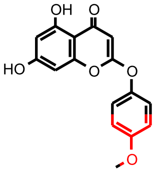 [*]O[c](:[cH]:[*]):[cH]:[*]	0.672

FCFP_2	1	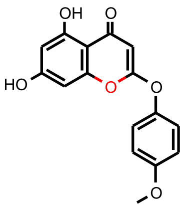 [*]O[*]	0.511
FCFP_2	451847724	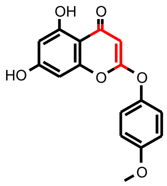 [*]C(=CC(=[*])[*])[*]	0.225

Top Features for negative contribution

Fingerprint	Bit/Smiles	Feature Structure	Score
FCFP_2	203677720	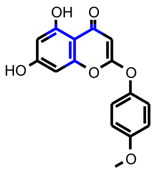 [*]C(=[*])[c](:[c]([*]):[*]):[c]([*]):[*]	-0.406
FCFP_2	1872154524	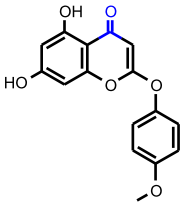 [*]C(=O)[*]	-0.307
FCFP_2	0	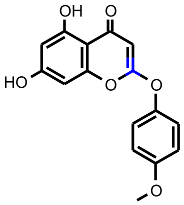 [*]C(=[*])[*]	-0.29

Flavonoid-2

TOPKAT_Rat_Maximum_Tolerated_Dose_Gavage

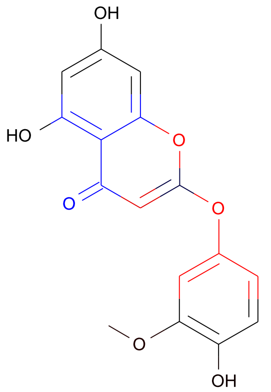

$C_{16}H_{12}O_7$
Molecular Weight: 316.26227
ALogP: 2.887
Rotatable Bonds: 3
Acceptors: 7
Donors: 3

Model Prediction

Prediction: 9.86e-005
Unit: g/kg_body_weight
Mahalanobis Distance: 11.1
Mahalanobis Distance p-value: 1.96e-007

Mahalanobis Distance: The Mahalanobis distance (MD) is a generalization of the Euclidean distance that accounts for correlations among the X properties. It is calculated as the distance to the center of the training data. The larger the MD, the less trustworthy the prediction.
Mahalanobis Distance p-value: The p-value gives the fraction of training data with an MD greater than or equal to the one for the given sample, assuming normally distributed data. The smaller the p-value, the less trustworthy the prediciton. For highly non-normal X properties (e.g., fingerprints), the MD p-value is wildly inaccurate.

Structural Similar Compounds

Name	OCHRATOXIN	PENICILLIN VK	SULFISOOXAZOLE
Structure	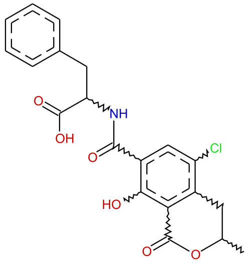	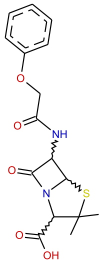	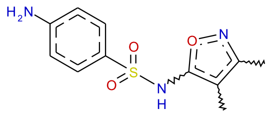
Actual Endpoint (-log C)	6.28396	2.54455	2.82494
Predicted Endpoint (-log C)	5.12358	3.9702	3.0705
Distance	0.609	0.807	0.837
Reference	NCI/NTP TR-358	NCI/NTP TR-336	NCI/NTP TR-138

Model Applicability

Unknown features are fingerprint features in the query molecule, but not found or appearing too infrequently in the training set.

1. Num_H_Acceptors out of range. Value: 7. Training min, max, mean, SD: 0, 6, 1.6146, 1.644.
2. OPS PC9 out of range. Value: 4.6683. Training min, max, SD, explained variance: -2.7086, 2.9267, 1.019, 0.0321.
3. Unknown FCFP_2 feature: -2115241127: [*]OC(=C[*])O[*]
4. Unknown FCFP_2 feature: -1549192822: [*]=CC(=O)[c](:[*]):[*]

Feature Contribution

Top features for positive contribution

Fingerprint	Bit/Smiles	Feature Structure	Score
FCFP_2	332760439	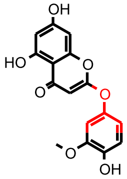 [*]O[c](:[cH]:[*]):[cH]:[*]	0.672

FCFP_2	1	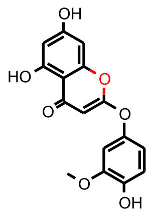 [*]O[*]	0.511
FCFP_2	451847724	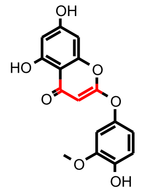 [*]C(=CC(=[*])[*])[*]	0.225
**Top Features for negative contribution**			
**Fingerprint**	**Bit/Smiles**	**Feature Structure**	**Score**
FCFP_2	203677720	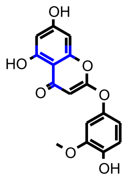 [*]C(=[*])[c](:[c]([*]):[*]):[c]([*]):[*]	-0.406
FCFP_2	1872154524	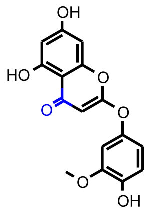 [*]C(=O)[*]	-0.307
FCFP_2	0	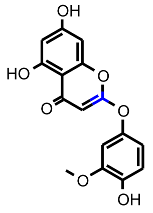 [*]C(=[*])[*]	-0.29

remdesivir

TOPKAT_Rat_Maximum_Tolerated_Dose_Gavage

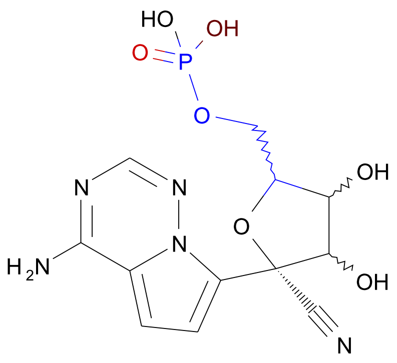

$C_{12}H_{14}N_5O_7P$

Molecular Weight: 371.24262

ALogP: -1.502

Rotatable Bonds: 4

Acceptors: 11

Donors: 5

Model Prediction

Prediction: 0.000298

Unit: g/kg_body_weight

Mahalanobis Distance: 17.2

Mahalanobis Distance p-value: 5.05e-016

Mahalanobis Distance: The Mahalanobis distance (MD) is a generalization of the Euclidean distance that accounts for correlations among the X properties. It is calculated as the distance to the center of the training data. The larger the MD, the less trustworthy the prediction.
Mahalanobis Distance p-value: The p-value gives the fraction of training data with an MD greater than or equal to the one for the given sample, assuming normally distributed data. The smaller the p-value, the less trustworthy the prediciton. For highly non-normal X properties (e.g., fingerprints), the MD p-value is wildly inaccurate.

Structural Similar Compounds

Name	AMPICILLIN TRIHYDRATE	OCHRATOXIN	PENICILLIN VK
Structure	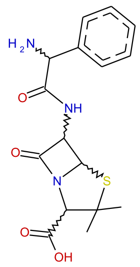	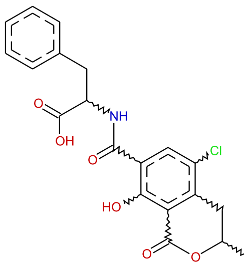	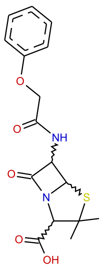
Actual Endpoint (-log C)	2.36724	6.28396	2.54455
Predicted Endpoint (-log C)	2.27651	5.12358	3.9702
Distance	1.255	1.482	1.498
Reference	NCI/NTP TR-318	NCI/NTP TR-358	NCI/NTP TR-336

Model Applicability

Unknown features are fingerprint features in the query molecule, but not found or appearing too infrequently in the training set.

1. Num_H_Donors out of range. Value: 5. Training min, max, mean, SD: 0, 3, 0.4375, 0.8311.
2. Num_H_Acceptors out of range. Value: 11. Training min, max, mean, SD: 0, 6, 1.6146, 1.644.
3. Molecular_PolarSASA out of range. Value: 321.97. Training min, max, mean, SD: 0, 223.97, 50.816, 55.15.
4. Molecular_PolarSurfaceArea out of range. Value: 206.26. Training min, max, mean, SD: 0, 138.03, 28.978, 32.1.
5. OPS PC1 out of range. Value: 9.0116. Training min, max, SD, explained variance: -4.0008, 7.9165, 2.861, 0.2531.
6. OPS PC5 out of range. Value: -4.1876. Training min, max, SD, explained variance: -3.4, 4.1587, 1.489, 0.0686.
7. OPS PC9 out of range. Value: -2.7276. Training min, max, SD, explained variance: -2.7086, 2.9267, 1.019, 0.0321.
8. Unknown FCFP_2 feature: 472180098: [*]OP(=O)(O)O
9. Unknown FCFP_2 feature: -836603894: [*]C1[*][*]O[C@]1(C#[*])[c](:[*]):[*]
10. Unknown FCFP_2 feature: -1277879912: [*]C([*])([*])C#N
11. Unknown FCFP_2 feature: -1362791977: [*]C#N
12. Unknown FCFP_2 feature: -332197802: [*][c]1:[*]:[*]:[c](:[*]):n:1:n:[*]
13. Unknown FCFP_2 feature: -124685461: [*]:n:[cH]:n:[*]

Feature Contribution

Top features for positive contribution

Fingerprint	Bit/Smiles	Feature Structure	Score
FCFP_2	1	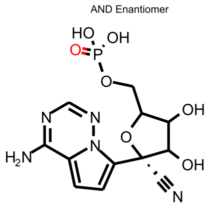 [*]O[*]	0.511
FCFP_2	3	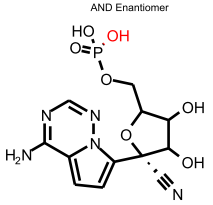 [*]O	0.104

Top Features for negative contribution

Fingerprint	Bit/Smiles	Feature Structure	Score
FCFP_2	1872154524	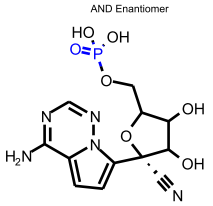 [*]C(=O)[*]	-0.307
FCFP_2	0	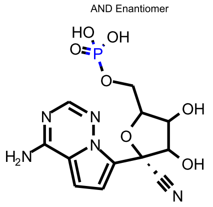 [*]C(=[*])[*]	-0.29

FCFP_2	-1272768868	AND Enantiomer 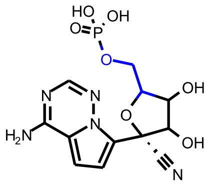 [*]OCC([*])[*]	-0.271

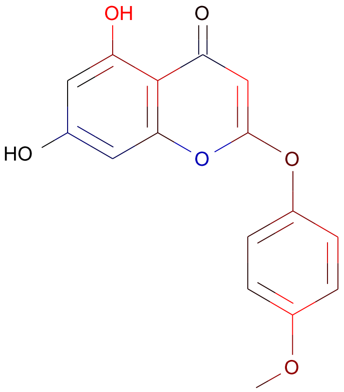

$C_{16}H_{12}O_6$

Molecular Weight: 300.26287

ALogP: 3.129

Rotatable Bonds: 3

Acceptors: 6

Donors: 2

Structural Similar Compounds

Name	FLUORESCEINE; SODIUM SALT (Na STRIPPED)	DICOUMAROL	2-(5-ISOPROPYL-5-METHYL-4-OXO-2-IMIDAZOLIN-2-YL)-3-QUINOLINECARBOXYLIC ACID
Structure	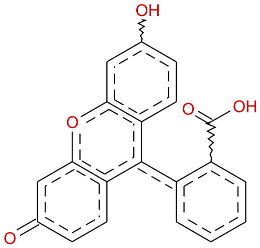	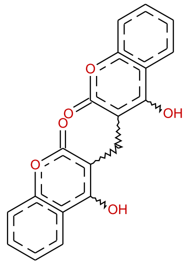	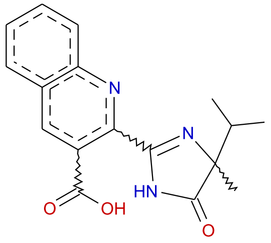
Actual Endpoint (-log C)	1.694	3.129	1.794
Predicted Endpoint (-log C)	2.71831	2.86156	1.66648
Distance	0.451	0.453	0.528
Reference	JOPRAJ 48;228;77	SMWOAS 83;471;53	FMCHA2 -;C257;89

Model Prediction

Prediction: 0.363

Unit: g/kg_body_weight

Mahalanobis Distance: 16.3

Mahalanobis Distance p-value: 0.158

Mahalanobis Distance: The Mahalanobis distance (MD) is a generalization of the Euclidean distance that accounts for correlations among the X properties. It is calculated as the distance to the center of the training data. The larger the MD, the less trustworthy the prediction.

Mahalanobis Distance p-value: The p-value gives the fraction of training data with an MD greater than or equal to the one for the given sample, assuming normally distributed data. The smaller the p-value, the less trustworthy the prediciton. For highly non-normal X properties (e.g., fingerprints), the MD p-value is wildly inaccurate.

Model Applicability

Unknown features are fingerprint features in the query molecule, but not found or appearing too infrequently in the training set.

1. All properties and OPS components are within expected ranges.
2. Unknown ECFP_2 feature: 367973906: [*]OC(=C[*])O[*]
3. Unknown FCFP_6 feature: 16: [*][c](:[*]):[*]
4. Unknown FCFP_6 feature: 1618154665: [*][c](:[*]):[cH]:[cH]:[*]
5. Unknown FCFP_6 feature: -2115241127: [*]OC(=C[*])O[*]
6. Unknown FCFP_6 feature: 74595001: [*]:[cH]:[c](O):[cH]:[*]
7. Unknown FCFP_6 feature: -549108873: [*]:[c](:[*])O

Feature Contribution

Top features for positive contribution			
Fingerprint	Bit/Smiles	Feature Structure	Score

ECFP_6	642810091	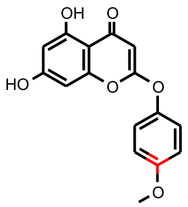<br>[*][c](:[*]):[*]	0.281
ECFP_6	-560785749	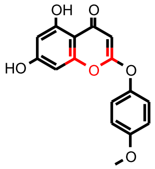<br>[*]C(=[*])O[c](:[*]):<br>[*]	0.259
FCFP_6	136627117	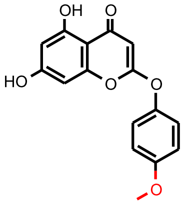<br>[*]OC	0.17
**Top Features for negative contribution**			
**Fingerprint**	**Bit/Smiles**	**Feature Structure**	**Score**
ECFP_6	2106656448	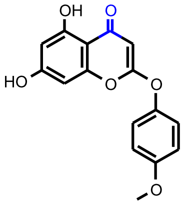<br>[*]C(=O)[*]	-0.352
ECFP_6	683445015	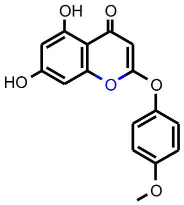<br>[*]O[*]	-0.266

ECFP_6	-176455838	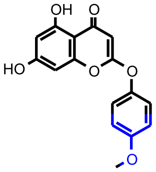<br>[*]O[c](:[cH]:[*]):[c<br>H]:[*]	-0.257

Flavonoid-2

TOPKAT_Rat_Oral_LD50

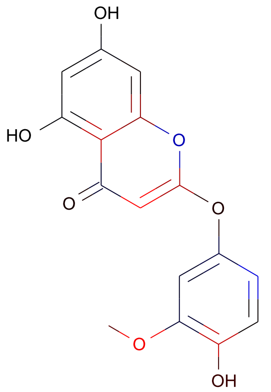

$C_{16}H_{12}O_7$

Molecular Weight: 316.26227

ALogP: 2.887

Rotatable Bonds: 3

Acceptors: 7

Donors: 3

Model Prediction

Prediction: 0.549

Unit: g/kg_body_weight

Mahalanobis Distance: 16.4

Mahalanobis Distance p-value: 0.133

Mahalanobis Distance: The Mahalanobis distance (MD) is a generalization of the Euclidean distance that accounts for correlations among the X properties. It is calculated as the distance to the center of the training data. The larger the MD, the less trustworthy the prediction.

Mahalanobis Distance p-value: The p-value gives the fraction of training data with an MD greater than or equal to the one for the given sample, assuming normally distributed data. The smaller the p-value, the less trustworthy the prediciton. For highly non-normal X properties (e.g., fingerprints), the MD p-value is wildly inaccurate.

Structural Similar Compounds

Name	DICOUMAROL	FLUORESCEINE; SODIUM SALT (Na STRIPPED)	OCHRATOXIN A
Structure	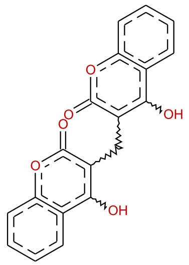	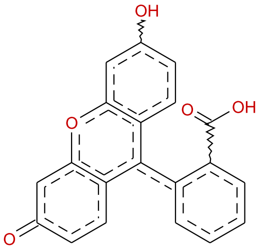	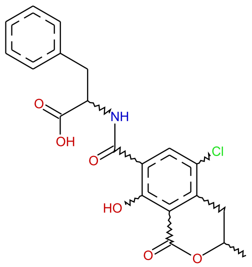
Actual Endpoint (-log C)	3.129	1.694	4.305
Predicted Endpoint (-log C)	2.86156	2.71831	3.03558
Distance	0.560	0.610	0.614
Reference	SMWOAS 83;471;53	JOPRAJ 48;228;77	FCTXAV 6;479;68

Model Applicability

Unknown features are fingerprint features in the query molecule, but not found or appearing too infrequently in the training set.

1. All properties and OPS components are within expected ranges.
2. Unknown ECFP_2 feature: 367973906: [*]OC(=C[*])O[*]
3. Unknown FCFP_6 feature: 16: [*][c](:[*]):[*]
4. Unknown FCFP_6 feature: 1618154665: [*][c](:[*]):[cH]:[cH]:[*]
5. Unknown FCFP_6 feature: 74595001: [*]:[cH]:[c](O):[cH]:[*]
6. Unknown FCFP_6 feature: -549108873: [*]:[c](:[*])O
7. Unknown FCFP_6 feature: -2115241127: [*]OC(=C[*])O[*]

Feature Contribution

Top features for positive contribution			
**Fingerprint**	**Bit/Smiles**	**Feature Structure**	**Score**

ECFP_6	642810091	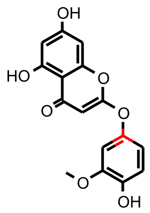 [*][c](:[*]):[*]	0.281
ECFP_6	-560785749	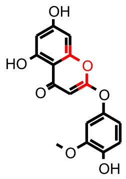 [*]C(=[*])O[c](:[*]):[*]	0.259
FCFP_6	136627117	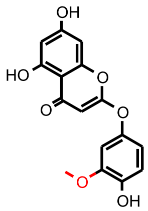 [*]OC	0.17

Top Features for negative contribution

Fingerprint	Bit/Smiles	Feature Structure	Score
ECFP_6	2106656448	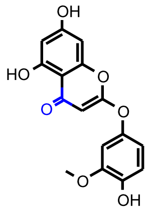 [*]C(=O)[*]	-0.352
ECFP_6	683445015	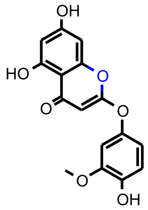 [*]O[*]	-0.266

ECFP_6	-176455838	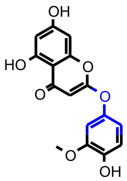<br>[*]O[c](:[cH]:[*]):[cH]:[*]	-0.257

remdesivir

TOPKAT_Rat_Oral_LD50

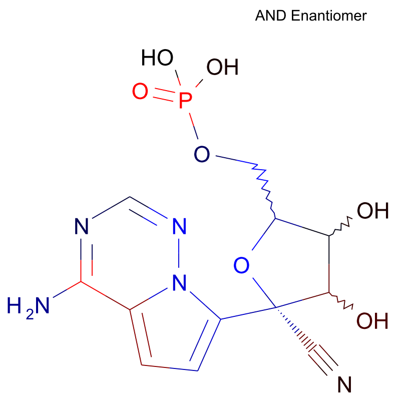

$C_{12}H_{14}N_5O_7P$

Molecular Weight: 371.24262

ALogP: -1.502

Rotatable Bonds: 4

Acceptors: 11

Donors: 5

Model Prediction

Prediction: 0.309

Unit: g/kg_body_weight

Mahalanobis Distance: 29.4

Mahalanobis Distance p-value: 1.72e-059

Mahalanobis Distance: The Mahalanobis distance (MD) is a generalization of the Euclidean distance that accounts for correlations among the X properties. It is calculated as the distance to the center of the training data. The larger the MD, the less trustworthy the prediction.
Mahalanobis Distance p-value: The p-value gives the fraction of training data with an MD greater than or equal to the one for the given sample, assuming normally distributed data. The smaller the p-value, the less trustworthy the prediciton. For highly non-normal X properties (e.g., fingerprints), the MD p-value is wildly inaccurate.

Structural Similar Compounds

Name	5'-ADENYLIC ACID; POTASSIUM SALT (K STRIPPED)	INOSINATE; DISODIUM SALT (Na STRIPPED)	INOSINE-5'-PHOSPHORIC ACID
Structure	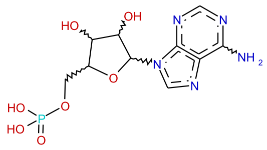	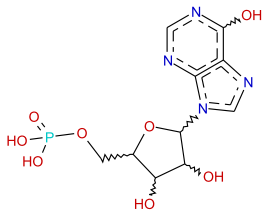	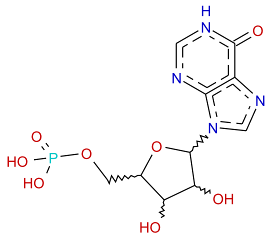
Actual Endpoint (-log C)	1.49	1.34	1.338
Predicted Endpoint (-log C)	2.45569	2.92201	1.35922
Distance	0.361	0.428	0.592
Reference	OYYAA2 4;689;70	AJINO* -;-;73	ARTODN 47;77;81

Model Applicability

Unknown features are fingerprint features in the query molecule, but not found or appearing too infrequently in the training set.

1. OPS PC10 out of range. Value: 15.526. Training min, max, SD, explained variance: -6.0395, 14.892, 2.468, 0.0220.
2. Unknown ECFP_2 feature: 1258791451: [*]C1[*][*]O[C@]1(C#[*])[c](:[*]):[*]
3. Unknown ECFP_2 feature: -264833661: [*]C([*])([*])C#N
4. Unknown ECFP_2 feature: -66263742: [*]C([*])([*])[c]1:[cH]:[*]:[*]:n:1:[*]
5. Unknown FCFP_6 feature: 16: [*][c](:[*]):[*]
6. Unknown FCFP_6 feature: 472180098: [*]OP(=O)(O)O
7. Unknown FCFP_6 feature: -836603894: [*]C1[*][*]O[C@]1(C#[*])[c](:[*]):[*]
8. Unknown FCFP_6 feature: -332197802: [*][c]1:[*]:[*]:[c](:[*]):n:1:n:[*]
9. Unknown FCFP_6 feature: 4427049: [*]:[cH]:n:n(:[*]):[*]
10. Unknown FCFP_6 feature: -124685461: [*]:n:[cH]:n:[*]
11. Unknown FCFP_6 feature: 1747237384: [*][c](:[*]):n:[cH]:[*]
12. Unknown FCFP_6 feature: -1151884458: [*]:n:[c](N):[c](:[*]):[*]
13. Unknown FCFP_6 feature: 1618154665: [*][c](:[*]):[cH]:[cH]:[*]
14. Unknown FCFP_6 feature: 1069584379: [*]:[c](:[*])N

Feature Contribution

Top features for positive contribution

Fingerprint	Bit/Smiles	Feature Structure	Score
ECFP_6	642810091	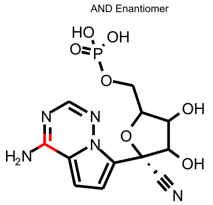 [*][c](:[*]):[*]	0.281
ECFP_6	-826638028	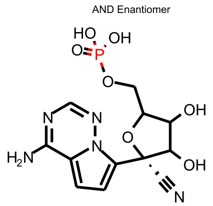 [*]P(=[*])([*])[*]	0.225
ECFP_6	2100964382	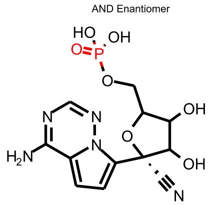 [*]P(=O)([*])[*]	0.166
**Top Features for negative contribution**			
**Fingerprint**	**Bit/Smiles**	**Feature Structure**	**Score**
ECFP_6	683445015	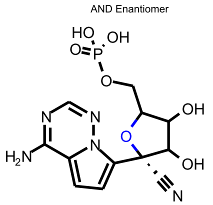 [*]O[*]	-0.266

ECFP_6	655739385	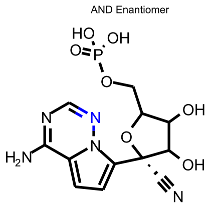<br>[*]:n:[*]	-0.239
FCFP_6	-1539132615	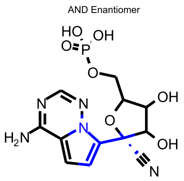<br>[*]C([*])([*])[c]1:[cH]:[*]:[*]:n:1:[*]	-0.2
